# Adaptive evolution of multiple-variable exons and structural diversity of drug-metabolizing enzymes

**DOI:** 10.1186/1471-2148-7-69

**Published:** 2007-05-02

**Authors:** Can Li, Qiang Wu

**Affiliations:** 1 Department of Human Genetics, University of Utah, Salt Lake City, Utah 84112, USA

## Abstract

**Background:**

The human genome contains a large number of gene clusters with multiple-variable-first exons, including the drug-metabolizing UDP glucuronosyltransferase (*UGT1*) and I-branching β-1,6-N-acetylglucosaminyltransferase (*GCNT2*, also known as IGNT) clusters, organized in a tandem array, similar to that of the protocadherin (*PCDH*), immunoglobulin (*IG*), and T-cell receptor (*TCR*) clusters. To gain insight into the evolutionary processes that may have shaped their diversity, we performed comprehensive comparative analyses for vertebrate multiple-variable-first-exon clusters.

**Results:**

We found that there are species-specific variable-exon duplications and mutations in the vertebrate *Ugt1*, *Gcnt2*, and *Ugt2a *clusters and that their variable and constant genomic organizations are conserved and vertebrate-specific. In addition, analyzing the complete repertoires of closely-related *Ugt2 *clusters in humans, mice, and rats revealed extensive lineage-specific duplications. In contrast to the *Pcdh *gene clusters, gene conversion does not play a predominant role in the evolution of the vertebrate *Ugt1, Gcnt2 *and *Ugt2 *gene clusters. Thus, their tremendous diversity is achieved through "birth-and-death" evolution. Comparative analyses and homologous modeling demonstrated that vertebrate UGT proteins have similar three-dimensional structures each with N-terminal and C-terminal Rossmann-fold domains binding acceptor and donor substrates, respectively. Molecular docking experiments identified key residues in donor and acceptor recognition and provided insight into the catalytic mechanism of UGT glucuronidation, suggesting the human UGT1A1 residue histidine 39 (H39) as a general base and the residue aspartic acid 151 (D151) as an important electron-transfer helper. In addition, we identified four hypervariable regions in the N-terminal Rossmann domain that form an acceptor-binding pocket. Finally, analyzing patterns of nonsynonymous and synonymous nucleotide substitutions identified codon sites that are subject to positive Darwinian selection at the molecular level. These diversified residues likely play an important role in recognition of myriad xenobiotics and endobiotics.

**Conclusion:**

Our results suggest that enormous diversity of vertebrate multiple variable first exons is achieved through birth-and-death evolution and that adaptive evolution of specific codon sites enhances vertebrate *UGT *diversity for defense against environmental agents. Our results also have interesting implications regarding the staggering molecular diversity required for chemical detoxification and drug clearance.

## Background

Alternative splicing is one of the most important mechanisms to generate molecular diversity in vertebrates. A large number of alternatively spliced genes that have multiple"variable" first exons have been identified in the human genome, including protocadherin (*PCDH*), UDP-glucuronosyltransferase (*UGT*), plectin (*PLEC1*), neuronal nitric oxide synthase (*NOS1*), and glucocorticoid receptor (*GR*) genes [1]. In particular, the closely-linked vertebrate *Pcdh α *and *γ *clusters have a striking genomic organization each containing more than a dozen variable first exons and three downstream "constant" exons [2-7]. Alternative splicing of each variable exon to the common set of constant exons generates diverse functional mRNA molecules that encode a large number of cadherin-like cell-surface proteins in the central nervous system (CNS). Comparative analyses suggest that gene duplication, gene conversion, and variable exon mutation play important roles in vertebrate *Pcdh *evolution [3-5]. In addition, adaptive selection of specific residues in the ectodomains enhances mammalian *Pcdh *diversity [5]. Combinatorial interactions between these Pcdh proteins contribute to the establishment and maintenance of trillions of diverse yet very specific neuronal connections in the vertebrate CNS.

In the vertebrate adaptive immune system, the immunoglobulin (*Ig*), T-cell receptor (*Tcr*), and major histocompatibility complex (*Mhc*) gene clusters provide the enormous diversity required for immune defense. The *Ig *and *Tcr *clusters are organized into variable and constant regions. Gene duplications and somatic DNA rearrangements generate tremendous diversity for mammalian Ig and Tcr molecules. Moreover, positive natural selection operates on the complementarity-determining regions (CDRs) of the IG and TCR proteins to increase their diversity [8-11]. The *Mhc *genes are also clustered. The encoded MHC proteins (both class I and II human HLA molecules) have a deep peptide-binding groove formed by a β-sheet bottom floor and two α-helix side walls [12,13]. Each MHC protein can bind a large set of different peptides. In addition, most of the polymorphic residues on the β-sheet floor and two α-helix side walls point towards the peptide-binding groove and serve as ligands for numerous processed antigens [14-16]. Finally, diversity-enhancing overdominant selection operates on the antigen-binding sites of both class I and II MHC proteins enabling them to recognize diverse processed antigens [11,17,18]. Vertebrate animals evolved these three gene families through birth-and-death evolution of repeated duplication and mutation, in conjunction with positive selection, to remove a staggering number of different foreign antigens in a highly specific fashion [11,19].

Vertebrate animals also remove hundreds of thousands xeno- and endobiotic lipophilic compounds from their bodies by converting them to water-soluble glucuronides through glucuronidation [20]. This detoxification pathway converts lipophilic aglycones to hydrophilic molecules and facilitates their excretion from the body. Glucuronidation is catalyzed by members of the UGT glucuronosyltransferase proteins in the endoplasmic reticulum (ER) [21,22]. Vertebrate UGT proteins belong to a large supergene family of ubiquitous glycosyltransferases (GT) [23] (currently >10,000, classified into 87 subfamilies). Diverse members of the GT superfamily are noted for their low sequence similarity but, surprisingly, belong to only two structural folds (GT-A and GT-B) [24].

Glucuronidation is also an important pathway for biotransformation and clearance of drugs, such as colorectal cancer drug irinotecan [25-27]. Genetic polymorphisms or mutations in human *UGT *genes have profound impacts on hyperbilirubinemia, drug metabolism, and cancer treatment [20,27-29]. For example, mutations of the human *UGT1A1 *gene cause genetic diseases with phenotypes ranging from mild jaundice to lethal kernicterus [20,28,30,31].

**Figure 1:**
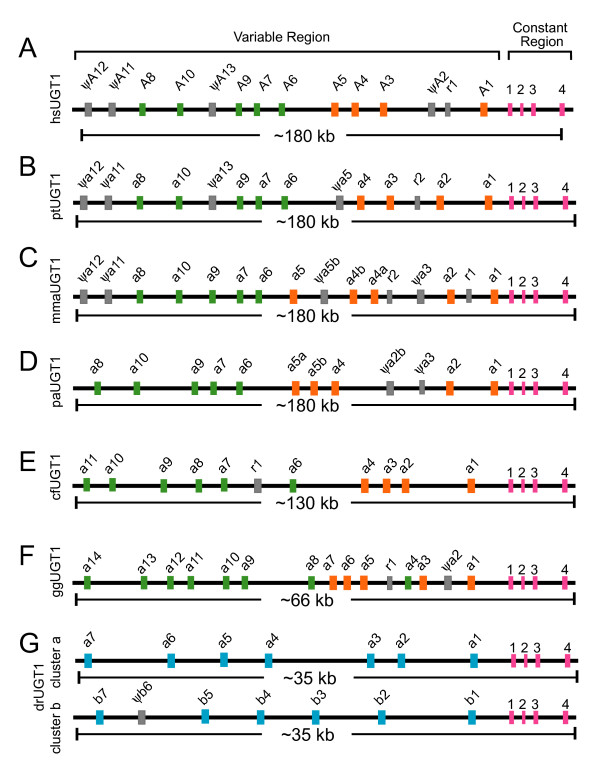
Comparison of the (**A**) human (*Homo sapiens *[hs]), (**B**) chimpanzee (*Pan troglodydes *[pt]), (**C**) rhesus monkey (*Macaca mulatta *[mma]), (**D**) baboon (*Papio anubis *[pa]), (**E**) dog (*Canis familiaris *[cf]), (**F**) chicken (*Gallus gallus *[gg]), and (**G**) zebrafish (*Danio rerio *[dr]) *Ugt1 *clusters. Each cluster contains multiple variable first exons arrayed in tandem and a common set of 4 downstream constant exons. These exons are indicated by vertical colored bars: (green) phenol-group variable exons; (orange) bilirubin-group variable exons; (blue) zebrafish variable exons; (gray) pseudogene (ψ) or relic (r); and (red) constant exons. The approximate length of each cluster is shown below the corresponding panels.

The human *UGT1 *cluster has an unusual genetic structure (Fig. 1A) which is strikingly similar to that of the *Pcdh *clusters [1]. Specifically, the human *UGT1 *cluster is organized into variable and constant regions [1,21,29,32,33]. About a dozen very similar human, mouse, and rat *UGT1 *variable exons (divided into bilirubin and phenol groups) are organized in a tandem array, which are followed by four constant exons (Fig. 1A) [1,33,34]. Splicing of each variable exon to the four constant exons generates diverse functional *UGT1 *mRNAs. Each variable exon encodes a signal peptide and the N-terminal aglycone-recognition domain of a UGT1 protein. The four constant exons encode the common C-terminal domain that binds the UDP glucuronic acid (UDPGA) donor substrate and an ER-anchoring transmembrane segment.

The role of the *UGT *genes in metabolizing myriad xeno- and endobiotic compounds suggests that natural selection may have played an important role in shaping their variation; however, the effects selection might have on such unusual genomic structures are unclear. To gain insight into the evolution of multiple variable first exons, we annotated the complete vertebrate *Ugt1*, *Gcnt2*, and *Ugt2a *repertoires and identified 65 (for mRNA and protein sequences see Additional file 1), 16 (Additional file 2), and 16 (Additional file 3) new genes, respectively. Phylogenetic analyses on these clusters revealed lineage-specific duplications of variable exons and conservation of constant exons. Our results suggest that functional diversity of these clusters is achieved through the birth-and-death evolution of variable exon duplication, divergence, and deletion, but conservation of the constant exons, which are essential in maintaining their basic functions. In addition, analyzing the complete repertoires of closely-related *Ugt2b *clusters in humans, mice, and rats identified a new rat *Ugt2b *gene (designated *Ugt2b39*; Additional file 3) and revealed extensive lineage-specific duplications.

To gain insight into the catalytic mechanisms of glucuronidation by diverse UGT glucuronosyltransferases, we sought evidence for structural features in donor and acceptor recognition by combined comparative analysis and homologous modeling [35]. We built the first three-dimensional (3D) structure model of the vertebrate UGT proteins based on sequence analyses of 91 UGT1 and 35 UGT2 GT-B proteins, and the known crystal structures of the non-vertebrate GT-B glycosyltransferases. Molecular docking of donor and acceptor ligands to the human UGT1A1 structure shed light on the specificity of the donor recognition and diversity of acceptor bindings. In particular, we identified four hypervariable regions within the N-terminal domain that form a potential acceptor-binding pocket. We also identified *Ugt *codon sites that may have been subject to Darwinian positive selection during vertebrate evolution by analyzing patterns of nucleotide (nt) substitutions at individual codon sites. Interestingly, the diversified residues in the four hypervariable regions map to an acceptor-binding pocket. These residues likely contribute to the required specificity for binding numerous hydrophobic small molecules. These results suggest that adaptive natural selection of specific codon sites plays an important role for enhancing UGT diversity. In summary, our results provide insight into the evolution of multiple variable exons and structural diversity of UGT proteins required for the removal of numerous xenobiotic compounds and endogenous metabolites.

## Results and Discussion

### The vertebrate *Ugt1 *gene cluster

We analyzed *Ugt1 *locus in a set of diverse vertebrate species including primates, non-primate mammals, birds, and fish (Fig. 1 and Additional file 1). Chimpanzees are the closest living relatives of humans, and their genomic sequences are highly similar to those of humans. We found that two *Ugt1 *genes differ between humans and chimpanzees (Fig. 1A and 1B) although these species only diverged as recently as several million years ago [36]. The chimpanzee *Ugt1a2 *has a complete open reading frame suggesting being a functional gene while the human *UGT1A2 *has a single-nt deletion at coding position 127 causing a frameshift. In contrast, the human *UGT1A5 *is a functional gene while the chimpanzee *Ugt1a5 *appears to be a pseudogene because its sequences have a single-nt deletion at coding position 704. This frameshift deletion is confirmed by more than 10 different sequence reads.

We also annotated the rhesus monkey and baboon *Ugt1 *clusters (Fig. 1C and 1D). The *Ugt1 *clusters in these two old-world-monkey species contain one more functional variable exon than the human and chimpanzee *Ugt1 *clusters. Specifically, the bilirubin group (*Ugt1 a1*-*a5*) is expanded in these two species. Compared with humans and chimpanzees, the *Ugt1a5 *appears to have been duplicated to *Ugt1a5a *and *Ugt1a5b *in both rhesus monkey and baboon (Fig. 1C and 1D). The functional duplication of *Ugt1a5 *in baboon has also been reported in a very recent publication [37]. However, the *Ugt1a5b *has been mutated to a pseudogene in rhesus monkey because its sequences have a single-nt insertion in the coding region (Fig. 1C). In addition, the *Ugt1a3 *has been mutated to a pseudogene in both rhesus monkey and baboon. Finally, the rhesus monkey *Ugt1a4 *variable exon appears to have been duplicated to *Ugt1a4a *and *Ugt1a4b *(Fig. 1C). Similarly, the baboon *Ugt1a2 *variable exon has also been duplicated; however, one duplicated copy has been mutated to a pseudogene *Ugt1a2b *(Fig. 1D). Interestingly, the rhesus monkey *Ugt1a7 *in the whole-genome-shotgun traces has no stop codon mutation; however, the *Ugt1a7 *in the finished BAC clone (Accession No. AC171066.4) has a stop codon at coding position 670. This observation suggests that *Ugt1a7 *has both functional and nonfunctional alleles segregating in the rhesus monkey population.

Dogs belong to the order Carnivora within the Laurasiatheria clade of mammals; while primates and rodents belong to the Euarchontoglire clade [38]. Dogs diverged from humans at about 94 million years ago while rodents diverged from primates at about 85 million years ago. The dog *Ugt1 *cluster contains 10 functional variable exons (Fig. 1E). Members of the dog *Ugt1 *cluster can also be divided into the bilirubin and phenol groups. Compared with the primate *Ugt1 *cluster, the genomic region of the dog *Ugt1 *cluster is about 50 kb smaller (Fig. 1E).

The chicken separated from mammals about 310 million years ago [39]. Similar to the mammalian *Ugt1 *clusters, the chicken *Ugt1 *cluster is also organized into variable and constant regions and it has 14 variable exons arrayed in tandem, including one with frameshift mutations (Fig. 1F). Members of the chicken *Ugt1 *cluster can also be separated into bilirubin and phenol groups. The genomic region of the chicken *Ugt1 *cluster is much smaller than that of mammals (Fig. 1F).

The zebrafish has supernumerary *Pcdh *genes organized into two duplicated clusters [4,5,7]. Consistent with whole genome duplications in the teleost fish species and similar to the duplication of the zebrafish *Pcdh *clusters [4,5,7], the zebrafish *Ugt1 *cluster has been duplicated into the *Ugt1 a *and *b *clusters each organized into variable and constant regions (Fig. 1G). In contrast to the vast expansion of the zebrafish *Pcdh *variable regions compared with mammals, the zebrafish *Ugt1a *and *Ugt1b *variable regions have not expanded. Specifically, compared with about a dozen *Ugt1 *variable exons in mammals, the zebrafish *Ugt1a *cluster only has seven functional variable exons, while the zebrafish *Ugt1b *cluster has only six functional variable exons (Fig. 1G). In total, we identified 13 novel zebrafish *Ugt1 *variable exons. Both zebrafish *Ugt1a *and *Ugt1b *clusters span a region of about 35 kb genomic sequences, much smaller than other vertebrate species analyzed.

The constant regions of mammalian, avian, and fish *Ugt1 *clusters are highly conserved and each contain 4 constant exons (Fig. 1). The length of each constant exon is identical among all vertebrate species except that the fourth constant exons are slightly smaller in frogs and zebrafish, encoding shorter polypeptides (Additional file 4). The two zebrafish constant sequences are highly similar with a 70% identity at the nt level and a 78% similarity at the polypeptide level. This observation strongly suggests that the two zebrafish *Ugt1 *clusters are duplicated from a single ancestral cluster. The polypeptides encoded by constant regions are highly conserved in vertebrates (Additional file 4).

### Evolutionary relationship among members of the vertebrate *Ugt1 *clusters

**Figure 2:**
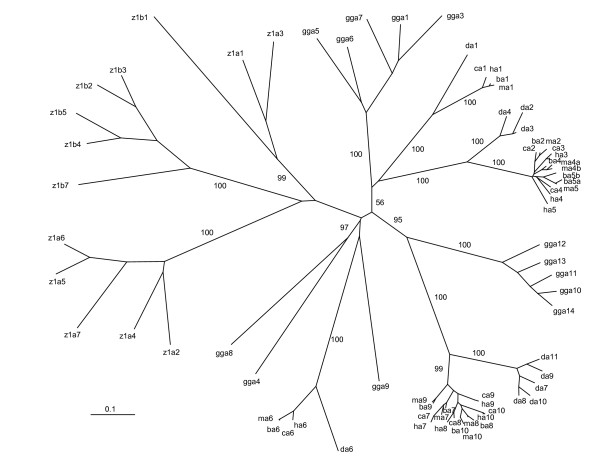
Phylogenetic tree of human (h), chimpanzee (c), rhesus monkey (m), baboon (b), dog (d), chicken (*Gallus gallus *[gg]), and zebrafish (z) *Ugt1 *clusters. The major tree branches are labeled with the percentage support (only when >50%) for that partition based on 1,000 bootstrap replicates. The scale bar equals a distance of 0.1.

Similar to the *Pcdh *clusters [40], the variable region of each vertebrate UGT1 protein is encoded by a single unusually large exon (Fig. 1). These *Ugt1 *variable exons are similar and are of almost identical length with the same reading frame. Each human, mouse, and rat *Ugt1 *variable exon is preceded by a distinct promoter [1,20,21,32,33]. Consistently, there is a highly-conserved sequence motif upstream from each vertebrate *Ugt1 *variable exon (Additional file 5), suggesting that these conserved promoter motif sequences play a role in tissue-specific *Ugt1 *gene regulation in vertebrates. The encoded variable polypeptides have the same predicted 3D domain structures (see below). Each variable polypeptide consists of a signal sequence followed by an aglycone-recognition domain. Their evolutionary relationships are shown as an unrooted phylogenetic tree (Fig. 2). In conjunction with the clustered genomic organization (Fig. 1), the tree demonstrates that variable exons of the *Ugt1 *clusters are duplicated in tandem. Some members are maintained, while others are inactivated by deleterious mutations in specific vertebrate lineages, suggesting that birth-and-death evolution has occurred in the *Ugt1 *cluster.

The mammalian and avian *Ugt1 *clusters can be divided into two major groups (constant-proximal bilirubin group and constant-distal phenol group) (Fig. 1). These groups each have a long major branch while members within each group have relatively shorter secondary branches in the phylogenetic tree, suggesting that members within each group were duplicated recently (Fig. 2). The human, chimpanzee, baboon, rhesus monkey, and dog *Ugt1a1 *is orthologous. However, there is no obvious orthologous *Ugt1a1 *in the chicken *Ugt1 *cluster, suggesting that the specialization of bilirubin glucuronidation by *Ugt1a1 *occurs after the divergence of mammals and birds. Interestingly, the mammalian *Ugt1a6 *is orthologous and is remotely similar to three avian *Ugt1 *variable exons (*a4*, *a8*, and *a9*). This observation indicates that *Ugt1a6 *is more ancient than other *Ugt1 *members.

Members of the zebrafish *Ugt1 *clusters do not display orthologous relationships to those of the mammalian and avian *Ugt1 *clusters. Instead, they display paralogous relationships in one major branch of the phylogenetic tree (Fig. 2). They can be divided into three subgroups: subgroup 1 includes *z1a1*, *z1a3*, and *z1b1*, subgroup 2 includes *z1b2-b7*, and subgroup 3 includes *z1a2 *and *z1a4-a7*. The zebrafish *Ugt1 a *and *b *variable exons and the corresponding constant exons seem to have resulted from a duplication of an ancestral one-variable *Ugt1 *gene. Subsequently, the variable exons in each cluster are duplicated multiple rounds. For example, the zebrafish *Ugt1 a1 *and *a2*, and *a3 *and *a4 *seem to be duplicated from an ancestral two-variable-exon unit because *a1 *and *a3*, and *a2 *and *a4 *share more sequence similarity, respectively. However, other zebrafish *Ugt1 a *and *b *variable exons seem to be duplicated in tandem because neighboring ones are more similar to each other.

Gene conversion plays an important role in the evolution of supergene families. Tandem gene arrays are often subject to sequence homogenization through gene conversion. For example, tandem arrayed *Pcdh *variable exons undergo strikingly predominant gene conversion events, especially among physically close exons [4]. To determine whether gene conversion played a similar prominent role in the evolution of vertebrate *Ugt1 *clusters, we used the Geneconv program [41] to search for gene conversion events among *Ugt1 *variable exons. Surprisingly, we did not find prevalent gene conversion events in the vertebrate *Ugt1 *clusters, except in the dog *Ugt1 *locus where gene conversion events have occurred in the phenol subgroup (*Ugt1 a7-a11*) (Additional file 6). Consistently, no gene conversion event was detected between any two functional baboon genes [37]. This observation suggests that, in striking contrast to the *Pcdh *clusters, concerted evolution does not play a predominant role in the evolvement of the vertebrate *Ugt1 *cluster.

### The organization and evolution of the vertebrate *Gcnt2 *cluster

**Figure 3:**
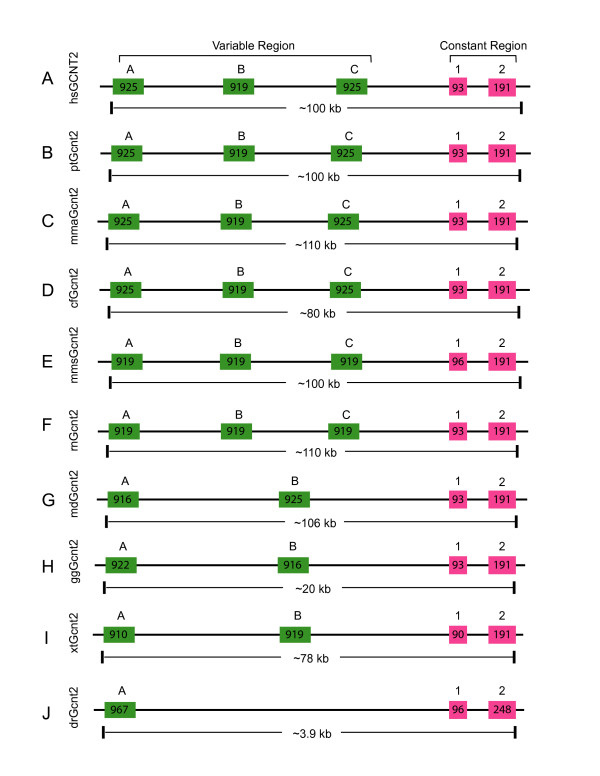
Comparison of the (**A**) human (*Homo sapiens *[hs]), (**B**) chimpanzee (*Pan troglodydes *[pt]), (**C**) rhesus monkey (*Macaca mulatta *[mma]), (**D**) dog (*Canis familiaris *[cf]), (**E**) mouse (*Mus musculus *[mms]), (**F**) rat (*Rattus norvegicus *[rn]), (**G**) opossum (*Monodelphis domestica *[md]), (**H**) chicken (*Gallus gallus *[gg]), (**I**) frog (*Xenopus tropicalis *[xt]), and (**J**) zebrafish (*Danio rerio *[dr]) *Gcnt2 *clusters. Each cluster contains multiple-variable and highly-similar first exons (green boxes) arrayed in tandem and a common set of two downstream constant exons (red boxes). Exon length is indicated within each box. The approximate length of each cluster is shown below the corresponding panels.

The human, mouse, and rat *Gcnt2 *(also known as *IGnT*) clusters each contain three highly similar variable exons and two constant exons [1] (Fig. 3A, 3E, and 3F). Each variable exon is preceded by a distinct promoter and is separately spliced to a set of two constant exons to generate functional *Gcnt2 *mRNA. We analyzed the vertebrate *Gcnt2 *clusters and found that the numbers of variable exons are different among mammals, birds, amphibians, and fishes (Fig. 3 and Additional file 2). The three *Gcnt2 *variable exons are conserved in chimpanzees and rhesus monkeys (Fig. 3B and 3C). They are also conserved in dogs, mice, and rats (Fig. 3D, 3E, and 3F). However, there are only two *Gcnt2 *variable exons in the opossum genome (Fig. 3G). Thus, the three *Gcnt2 *variable exons are conserved in primates, canids, and rodents but not opossums (Fig. 3A–G). There are only two *Gcnt2 *variable exons in the chicken and frog genomes (Fig. 3H and 3I). Finally, there is only one *Gcnt2 *variable exon in zebrafish (Fig. 3J). These results suggest that the tetrapod *Gcnt2 *variable exons were expanded by tandem duplication during vertebrate evolution.

The vertebrate *Gcnt2 *variable exons are about the same length and are very similar to each other. The encoded polypeptides are highly conserved (Additional file 7). Each *Gcnt2 *variable domain has a hydrophobic transmembrane segment close to the N-terminal (Additional file 7). They also contain six cysteine residues that are identical among all GCNT2 proteins (Additional file 7). An evolutionary tree was built according to the variable GCNT2 polypeptides (Additional file 8). The three *Gcnt2 *variable exons display orthologous relationships among all eutherian mammals. Interestingly, the two opossum *Gcnt2 *variable exons display a paralogous relationship and appear to be more similar to the eutherian *Gcnt2b *variable exons. The two chicken and frog *Gcnt2 *variable exons also display paralogous relationships and are divergent from the mammalian *Gcnt2 *variable exons. The single zebrafish *Gcnt2 *variable exon appears most closely related to the frog *Gcnt2 *variable exons. This result supports the hypothesis that the *Gcnt2 *variable exons have expanded in tetrapods through tandem duplications during vertebrate evolution. The genomic organization of the *Gcnt2 *constant region is highly conserved in vertebrates (Fig. 3). For example, the first constant exons of vertebrate *Gcnt2 *cluster are all 93 nts in length except in mice, frogs, and zebrafish, which are 96, 90, and 96 nts, respectively. The encoded constant protein sequences are conserved and have three identical cysteine residues (Additional file 9).

### The vertebrate *Ugt2 *cluster

We previously identified more than three thousand human genes with multiple first exons through a genome-wide computational analysis; however, only the first exons of the *PCDH *and *GCNT2 *clusters are highly similar [1]. We have noted that the genomic organization of the human *UGT2A *cluster [42] is also similar to that of the *UGT1*, *PCDH*, and *GCNT2 *clusters. In particular, the C-terminal domains of the human UGT2A proteins are identical and are encoded by a set of five constant exons; by contrast, the N-terminal domains are similar and each is encoded by a single variable exon. However, in contrast to the human *UGT1 *cluster, the human *UGT2A *cluster only contains two variable exons which share 64% nt sequence identity. The variable and constant organizations of the *Ugt2a *cluster are conserved in human, mouse, and rat genomes (Additional file 10, panels A, B, and C). For example, the human, mouse and rat *Ugt2a *variable exons are similar and of the same length. We annotated the *Ugt2a *clusters in several additional mammalian species (Additional file 3) and found that the *Ugt2a *organization of two variable exons and five constant exons is conserved.

In contrast to the expansion of the mammalian *Ugt1 *clusters compared with zebrafish (Fig. 1), we found that the variable region of the zebrafish *Ugt2a *cluster is expanded in comparison to the mammalian variable regions and contains 4 novel variable exons (Additional files 3 and 10). Phylogenetic analysis demonstrates that the mammalian *Ugt2 a1 *and *a2 *variable exons display a strict orthologous relationship (Additional file 11). However, there is no orthologous relationship between mammalian and zebrafish *Ugt2a *variable exons. The four zebrafish *Ugt2a *variable exons appear to be duplicated in tandem, with the *Ugt2 a3 *and *a4 *duplicated most recently (Additional file 11). Multiple sequence alignment demonstrates that all vertebrate *Ugt2a *variable protein sequences are highly similar (Additional file 12). Like the constant region of the mammalian *Ugt2a *cluster, the zebrafish *Ugt2a *constant region contains 5 exons, which are highly similar to those of mammals. In particular, the sizes of constant exons 1 to 4 are identical between zebrafish and mammals, respectively. The zebrafish *Ugt2a *constant exon 5 coding region is 18 nts longer than the corresponding mammalian *Ugt2a *constant exon (Additional file 10, panels D and E). The polypeptides encoded by *Ugt2a *constant region are highly conserved in vertebrates (Additional file 13).

We performed a comprehensive analysis of the closely-related human, mouse, and rat *Ugt2b* genes and found one novel rat *Ugt2b* gene, designated *Ugt2b39 *(Additional file 10, panel C). The human, mouse, and rat *Ugt2b* genes are also clustered and are located very close to the *Ugt2a* cluster [21,33]. However, the genomic organizations of the human, mouse, and rat *Ugt2b *genes are different from the *Ugt2a *genes in that the *Ugt2b *genes do not share common constant exons. Each member of the *Ugt2b *cluster is an independent gene and contains six exons (Additional file 10). All corresponding exons are highly similar among members of the *Ugt2b *cluster. Their exon lengths are also identical among different mammalian species. The encoded UGT2B proteins are highly conserved among humans, mice, and rats. The transcription directions are the same for all members of the rat *Ugt2b *cluster, and are also the same for members of the rat *Ugt2a* genes. However, the transcription directions for members of the *Ugt2b* cluster are not the same in the human and mouse genomes (Additional file 10).

The evolutionary relationships of the *Ugt2b *genes are shown as an unrooted phylogenetic tree (Additional file 14). The human *UGT2B *genes display paralogous relationships while members of the mouse and rat *Ugt2b *clusters display both paralogous and orthologous relationships. For example, the mouse *Ugt2b1 *and *Ugt2b34 *appears to be orthologous to the rat *Ugt2b1 *and *Ugt2b34*, respectively. However, the rat *Ugt2b39 *and *Ugt2b34 *genes appear to be duplicated from an ancestral gene because they are very similar and are also located next to each other (Additional file 10). The other mouse and rat *Ugt2b *genes do not have orthologous relationships. The phylogenetic tree suggests that most human, mouse, and rat *Ugt2b *genes are duplicated after speciation (Additional file 14).

In summary, we analyzed the *Ugt1 *loci in chimpanzee, rhesus monkey, baboon, dog, chicken, and zebrafish, and identified 65 new vertebrate *Ugt1 *genes (Additional file 1). Phylogenetic analysis demonstrated that the avian and mammalian *Ugt1 *variable regions are expanded compared to zebrafish (Figs. 1 and 2). We also performed a comprehensive analysis of the vertebrate *Gcnt2 *cluster and identified 16 new *Gcnt2 *genes (Additional file 2), and found that the variable region of the *Gcnt2 *cluster is also expanded during vertebrate evolution (Fig. 3). Finally, we analyzed the vertebrate *Ugt2 *repertoires and found that, in contrast to *Ugt1 *and *Gcnt2 *clusters, the zebrafish *Ugt2a *variable region has been expanded compared with mammals (Additional file 10). These results suggest that these vertebrate variable exons are subject to lineage-specific birth-and-death evolution.

### Structure modeling of the vertebrate UGT proteins

**Figure 4:**
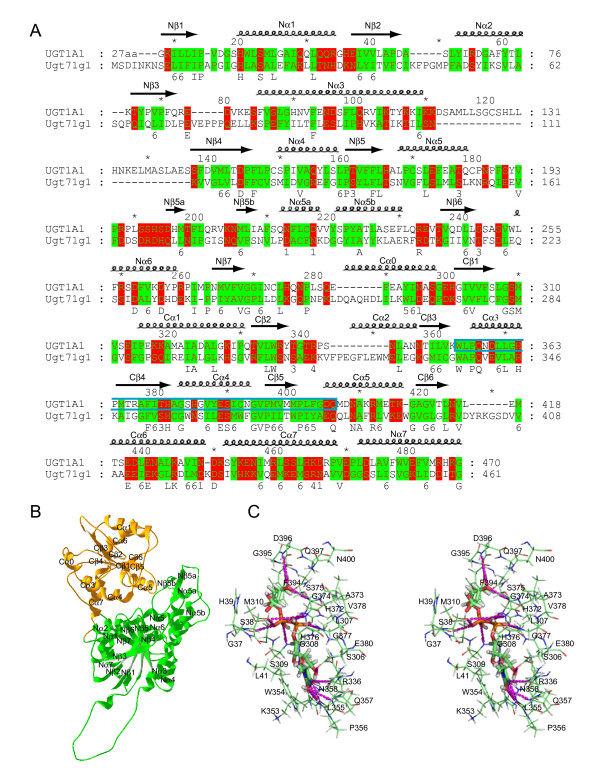
Modeling of the human UGT1A1 protein. (**A**) Structural alignment of the human UGT1A1 polypeptide with that of UGT71G1. The secondary structure elements are shown above the alignment. The 44-aa donor signature motif of UGT1A1 is enclosed by a cyan box. Broadly conserved hydrophilic and hydrophobic residues are highlighted with degree of conservation shown below the alignment. This panel was produced by the GeneDoc program [82]. (**B**) Ribbon diagram of the modeled 3D structure of the human UGT1A1. The N- and C-terminal domains are shown in green and orange, respectively. The α helices and β strands in the N- and C-terminal domains are labeled. This panel was made by Swiss-PdbViewer [78]. (**C**) Stereo diagram showing predicted interactions between the donor UDPGA and the UGT1A1 side chains. Hydrogen bonds are indicated by dashed lines. Figures 4C, 6B, 6C, and 8 were prepared with the Pymol [83].

The human UGT proteins allow our body to remove myriad endogenous metabolites and exogenous chemicals, such as steroids, bilirubin, bile acids, hormones, carcinogens, environmental toxicants, and therapeutic drugs [20,26]. Understanding their structures will shed light on the substrate specificity [22,26]. However, the 3D structure information, either based on X-ray data or molecular modeling, is not available to date. There are currently five crystal structures of the GT-B family members (MurG [43], GtfB [44], GtfA [45], GtfD [46], and UGT71G1 [47]). These structures are related although their primary sequences are divergent [24,47]. To comparatively model vertebrate UGT protein structures, we first aligned the bacterial and plant GT-B polypeptides based on their 3D structures. We then aligned this structure-based alignment to the human UGT1A1 sequence based on the predicted vertebrate UGT secondary structure profile. We also aligned 91 vertebrate UGT1 and 35 human, mouse, rat, and zebrafish UGT2 polypeptides. Each of these translated 126 polypeptides has a signal peptide at the N-terminal and a 17-amino-acid (aa) transmembrane segment close to the C-terminal with about 20 amino acids on the cytoplasmic side. The mature UGT proteins mostly reside in the lumen of the ER [22]. The structure of the human UGT1A1 within the ER lumen was modeled based on the alignment with UGT71G1 (Fig. 4A).

Our modeled 3D structure is consistent with that the vertebrate UGT proteins belong to the GT-B superfamily of the inverting glycosyltransferases [22,24]. Each modeled vertebrate UGT protein consists of two domains with similar core structure of Rossmann folds [48]. As an example, the modeled 3D structure of the human UGT1A1 protein is shown in Figure 4B. The N-terminal acceptor-binding domains of UGT1 proteins are each encoded by highly-similar variable exons in all vertebrate species (Fig. 1). The C-terminal donor-binding domains of UGT1 proteins are identical in each species and are encoded by four constant exons (Fig. 1). For UGT2 proteins, the acceptor-binding domains are encoded by first two exons which correspond to a single *Ugt1 *variable exon, and the donor-binding domains are encoded by the last four exons [21]. The C-terminal domains of all vertebrate UGT proteins are highly conserved and assumed to bind the donor UDPGA [22].

The N-terminal acceptor-binding domain of the modeled human UGT1A1 contains a central seven-parallel-strand β-pleated sheet with a topological arrangement of β3, β2, β1, β4, β5, β6, β7 (Fig. 4B). This core β sheet is flanked by 8 α helices. The first three β strands are connected by two α helices (arranged in α2 and α1 orientation) on the same side of the β sheet as the Nα7 helix, which is from the C-terminal sequences but is located below the last four β strands in the N-terminal domain. The other side of the core β sheet contains five helices with a topological arrangement of α3, α4, α5b, α5, α6. Similar to the structure of UGT71G1, there is a small two-stranded β sheet following the Nα5 helix. In contrast to the structure of UGT71G1, there is a flexible loop and a small predicted α helix following the Nα3 helix (Fig. 4B). This segment is predicted to have different conformations among different human UGT proteins.

The C-terminal donor-binding domain contains a central six-parallel-strand β-pleated sheet with a topology arrangement of β3, β2, β1, β4, β5, β6 (Fig. 4B). This β sheet core is flanked by 7 α helices with a topological arrangement of α0, α3, α4, α5 at one side of the β sheet and α1 and α6 at the other side of the β sheet, and α7 at the bottom. In contrast to UGT71G1, the human UGT1A1 does not appear to have the Cα2 helix. The last C-terminal α helix (Nα7) is located at the bottom of the N-terminal domain. The two loops between Nβ7 and Cα0 and between Cα7 and Nα7 connect the N-terminal and C-terminal domains (Fig. 4B).

### Interactions between UGT proteins and the donor substrate

The donor substrate UDPGA for vertebrate UGT enzymes is predicted in our 3D model to bind in a long narrow channel mainly in the C-terminal Rossmann-fold domain (Fig. 4B and 4C). In particular, the donor sits in a groove formed by the N-terminal half of the Cα3 and Cα4, and the C-terminal half of the Cβ4 and Cβ5. In the modeled donor-UGT1A1 complex, the uracil ring of UDPGA interacts with the side chain of R336 and the main chain of L355 and Q357 through hydrogen bonds, and also forms parallel stacking interaction with the indole ring of W354 of the human UGT1A1. The ribose ring of UDPGA interacts with the side chain of Q357, N358, and E380 through hydrogen bonds. The α-phosphate forms hydrogen bonds with the side chain of S38 and H372 and the main chain of H376 and G377, while the β-phosphate interacts with the side chain of S38 and H372 and the main chain of S309 and G37. Finally, the glucuronic acid moiety interacts with the side chain and main chain of D396, the main chain of S375, and the side chain of Q397 through hydrogen bonds (Fig. 4C). Overall, the donor binding mode is similar to that observed in the complex of donor substrates with the GtfB, GtfD, MurG, and UGT71G1 proteins [43,45-47]. In addition, this model of the donor recognition is consistent with the crucial role of the human UGT1A6 histidine, arginine, aspartic, and glutamic residues as demonstrated by chemical modification and site-directed mutagenesis experiments [22].

**Figure 5:**
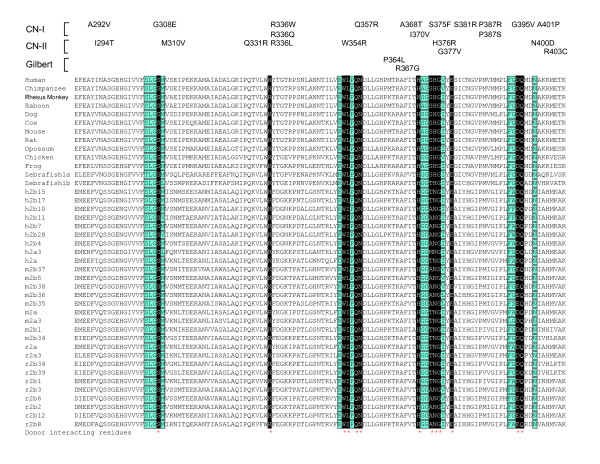
The donor-binding region of vertebrate UGT proteins. Shown is an alignment of the donor-binding region of the human, chimpanzee, rhesus monkey, baboon, dog, cow, mouse, rat, opossum, chicken, frog, and zebrafish UGT1 constant polypeptides and the corresponding donor-binding region of the human (h), mouse (m), and rat (r) UGT2A constant polypeptides and UGT2B proteins. Residues predicted to interact with the donor UDPGA are highlighted in white letters with black background and marked by red asterisks below. Residues close to the donor are highlighted with turquoise background and are also boxed. Missense mutations in the UGT1A1 that cause human CN-I, CN-II, or Gilbert syndromes are indicated above the alignment. For UGT1, constant polypeptides are shared by multiple UGT1A proteins in each species. For UGT2, individual protein sequence is shown except h2a, m2a, and r2a, which are the human, mouse, and rat UGT2A constant polypeptides, respectively.

The sequences of donor-binding region are highly conserved, especially for the donor-interacting residues. For example, the residues interacting with UDPGA are identical among all vertebrate UGT1 proteins and are almost identical among the UGT2 proteins (Fig. 5). In addition, the residues located very close to UDPGA are also almost identical among the UGT1 and UGT2 proteins (Fig. 5). Previous studies have predicted that UDPGA binds to this region of UGT proteins [22,49]. However, the D394 of UGT1A6 (corresponding to the D396 of UGT1A1) were predicted to interact with the uridyl moiety, an orientation different from the known GT-B donor complexes [43,45-47] and our modeled donor-UGT1A1 interactions (Fig. 4C).

UGT proteins use UDPGA as a specific donor substrate [20]. In our modeled UDPGA UGT1A1 complex, the side chains of D396 and Q397 interact with the glucuronic acid moiety. These two residues may play an important role in the specific recognition of donor molecule by the UGT proteins. Consistently, Q397 is conserved in all vertebrate UGT1 and UGT2 proteins (Fig. 5). Similarly, D396 is conserved in all vertebrate UGT1 proteins and all human UGT2 proteins. It is also conserved in mouse and rat UGT2 proteins with a few replaced by a glutamic residue.

Missense mutations of human UGT1A1 cause hyperbilirubinemia, including type I and II Crigler-Najjar syndromes (CN-I, OMIM no. 218800 and CN-II, OMIM no. 606785) and the Gilbert syndrome (OMIM no. 143500) [20,28,30]. Point mutations with amino acid substitutions A292V (referred as A291V in [50]), G308E [50], R336W [51], R336Q [52], Q357R [50], A368T [50], I370V [53], S375F (referred as S376F in [54]), S381R [50], P387R [55], P387S [52], G395V [52], or A401P [50] cause the CN-I disease (Fig. 5). Moreover, the missense substitutions I294T [51], M310V [56], Q331R [57], R336L [52], W354R [52], H376R (referred as H377R in [28]), G377V [28], N400D [58] or R403C [52] cause the CN-II disease (Fig. 5). Finally, point mutations of P364L [59], or R367G [60,61] cause the Gilbert syndrome (Fig. 5). These mutations are in the positions of highly conserved residues (with exception of only A292) in the donor-binding region (Figs. 4 and 5). In particular, the residues G308, M310, R336, W354, Q357, S375, H376, G377, S381, G395, N400, and A401 are located very close to the donor in the modeled 3D structure of human UGT1A1 protein (Figs. 4C and 5). Their mutations may interfere with the donor binding, thus abolishing or decreasing the bilirubin glucuronidation activity of the human UGT1A1 protein and cause hyperbilirubinemia.

### The UGT acceptor-binding site

**Figure 6:**
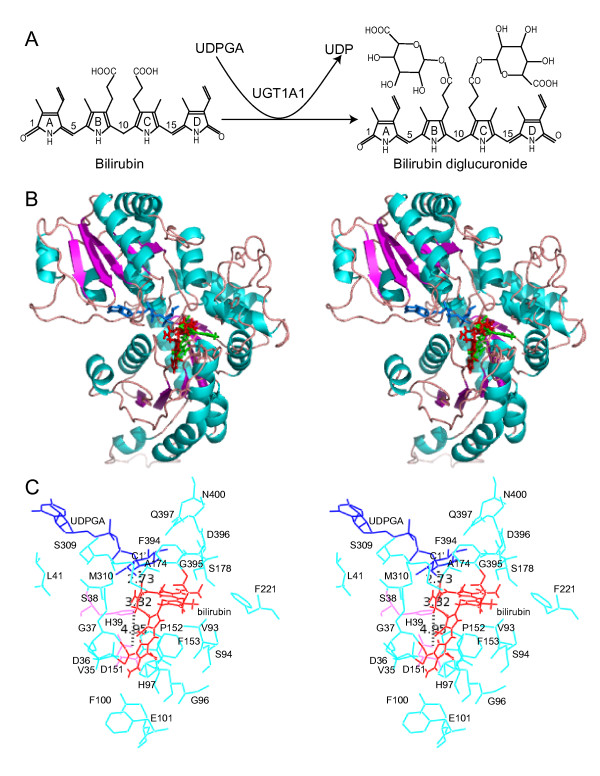
Molecular docking of bilirubin into the human UGT1A1 protein. (**A**) Diagram of the bilirubin glucuronidation reaction catalyzed by UGT1A1. (**B**) Stereo diagram showing positions of the modeled substrates. The UGT1A1 is shown in a ribbon diagram with the secondary structure highlighted. The donor UDP glucuronic acid (UDPGA) is shown as blue stick. The two docked conformations of bilirubin are shown as red and green sticks. (**C**) Stereo diagram showing bilirubin (red conformation) docked into the acceptor-binding pocket of UGT1A1. UDPGA (blue) and bilirubin (red) are shown as lines. Some residues in the acceptor-binding pocket are labeled and shown in cyan. Distances between the OH group of the bilirubin porphyrin C propionate and the C1' atom of the UDPGA or the NE2 atom of the residue histidine 39 (H39) (shown in pink), or between the NE2 atom of the residue H39 and the OD2 atom of the residue aspartic acid 151 (D151) (shown in pink) are indicated with dashed lines.

Bilirubin is highly lipophilic, unexcretable, and neurotoxic, and is known to have a stable ridge-tile conformation [62]. The human UGT1A1 is the only UGT1 enzyme for glucuronidating bilirubin acceptor substrate [31]. It catalyzes the transfer of the glucuronic acid moiety from UDPGA to the bilirubin C8 and C12 propionate groups (Fig. 6A). In the modeled human UGT1A1 structure, there is a deep kinked pocket adjacent to the donor-binding site (Fig. 6B). This pocket is formed mostly by the N-terminal domain. Specifically, the wall of the pocket is formed by the N1 and N2 turns following the Nβ1 and Nβ2 strands, the Nα3, Nα5b, Nα5, and Nα5a helices (Fig. 6B). The floor of the pocket is formed by the Nβ5 strand and the N4 turn following the Nβ4 strand (Fig. 6B). The location of the pocket is similar to that of the substrate binding sites observed in the complex of the GtfA or GtfD with their corresponding acceptor substrates, and in the modeled acceptor-binding site of UGT71G1 [45-47]. Therefore, this pocket is likely the acceptor-binding site of the human UGT1A1. The glucuronic acid moiety of the donor in the modeled complex is oriented toward the middle of this acceptor-binding pocket.

We modeled the bilirubin binding using the molecular docking software GOLD [63]. The ridge-tile conformation of bilirubin is docked into the hydrophobic pocket with the ridge apposing the donor molecule in the C-terminal domain, and the porphyrin ring A in the one end and the porphyrin ring D in the other end of the N-terminal acceptor pocket (Fig. 6B). The propionate side groups are in the middle and close to the glucuronic acid moiety of the donor molecules. Consistent with two glucuronidation sites in bilirubin through esterification of its two propionate side groups on the porphyrin rings B and C, there are two conformations that the bilirubin docked in the acceptor pocket (Fig. 6B), with each propionate side group docked close to the glucuronic acid moiety of the donor and the highly conserved catalytic residue H39. There is a small tilt between the two bilirubin docking conformations. The acceptor-binding pocket is much larger and longer than bilirubin, and bilirubin can be fit easily into the pocket with one of its two propionate OH groups located at about 3 angstrom (Å) from the NE2 atom of the H39 residue (Fig. 6C).

**Figure 7:**
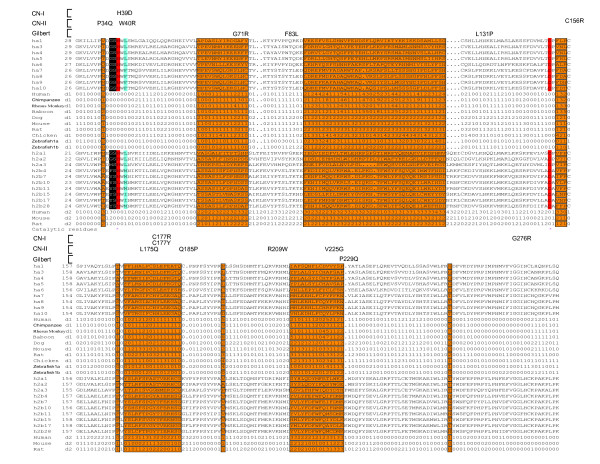
The acceptor-binding region of vertebrate UGT proteins. Shown is an alignment of the acceptor-binding region of the human UGT1 and UGT2 proteins. The diversity indexes (defined as K_A_/K_S _multiplied by 2) for the human, chimpanzee, rhesus monkey, baboon, dog, mouse, rat, chicken, and zebrafish UGT1 (d1) and the human, mouse, and rat UGT2 (d2) are also shown. The four hypervariable regions (with exceptions of two cysteines) and several other highly diversified residues are highlighted in orange background and are also boxed. A leucine residue (L41) close to the donor is highlighted in turquoise background. Residues predicted to interact with the donor molecule are highlighted with white letters in black background. Missense mutations in the human UGT1A1 that cause CN-I, CN-II, or Gilbert syndromes are indicated above the alignment. The two catalytic residues histidine 39 (H39) and aspartic acid 151 (D151) are highlighted in red background and with asterisks below.

The acceptor-binding pocket of the human UGT1A1 is surrounded by mostly hydrophobic residues in the N-terminal domain, including P34, V35, A64, L66, Y67, G71, F92, V93, G96, V99, F100, F153, F170, L172, A174, L175, F181, F221, V225, and A231. Interestingly, most of these hydrophobic residues are highly diversified among paralogous members of the vertebrate UGT proteins and are located within four major hypervariable regions in the N-terminal domain (Fig. 7). These four hypervariable regions form the wall and floor of the acceptor-binding pocket in the N-terminal domain (Figs. 6B and 7). Analogous to the recognition of antigens by hypervariable regions of the IG, TCR, and MHC proteins, we propose that these hypervariable regions play an important role in the recognition of numerous aglycone-acceptor substrates by vertebrate UGT1 and UGT2 proteins.

Missense mutations of P34Q [52], H39D [28], W40R [64], G71R [61], F83L [65], L131P (referred as L132P in [61]), C156R [66], L175Q [67], C177R [67], C177Y [66], Q185P [64], R209W [67,68], V225G (referred as V224G in [28,69]), P229Q [60,61], and G276R [67] in human UGT1A1 protein cause hyperbilirubinemia (Fig. 7). The P34, H39, and W40 residues are located in the acceptor-binding pocket and are also close to the donor substrate (Figs. 4, 6, and 7). The other mutated residues are mostly located in the four major hypervariable regions that form the acceptor-binding pocket. Therefore, these mutations may interfere with the binding of bilirubin to human UGT1A1 and abolish or decrease its bilirubin glucuronidating activity, consistent with the hyperbilirubinemia phenotypes. Interestingly, the UGT1A1 G71R mutation is almost exclusively found in Asians and has recently been shown to associate with severe cancer drug (i.e. irinotecan) toxicity [27], consistent with the altered acceptor recognition.

### Catalytic mechanism of vertebrate UGT glucuronidation

Vertebrate UGT proteins belong to the GT-B inverting glycosyltransferase supergene family (Fig. 4) [22,24]. However, little is known about their catalytic mechanisms. In our modeled human UGT1A1 structure with the donor UDPGA and acceptor bilirubin substrates, the NE2 atom of the H39 residue lies in the middle of the potential acceptor pocket and is close to both the OH group of the bilirubin propionate side group (~3.32 Å) and the C1' atom of UDPGA (~2.73 Å), suggesting a general SN2 catalytic mechanism for glucuronidation reactions (Fig. 6C).

We propose that the H39 of human UGT1A1 acts as a general base to abstract a proton from the OH group of the bilirubin propionate, based on the crystal structures of other GT-B enzymes [46,47]. A direct attack by the resulting nucleophilic oxyanion at the C1' atom of UDPGA would then displace the UDP moiety. Consistent with the essential role of H39, it is highly conserved in vertebrate UGT proteins (Fig. 7). In addition, an H39D mutation in human UGT1A1 gene causes CN-I disease [28], consistent with the complete abolishment of the catalytic activity for bilirubin glucuronidation (Fig. 7).

In the modeled human UGT1A1 structure, there is an acidic D151 residue close to H39 that may form an electron transfer chain to help H39 deprotonate the OH group of the acceptor molecule (Fig. 6C). This aspartic acid residue is also highly conserved in vertebrate UGT proteins in agreement with its essential role in catalysis (Fig. 7). Thus, our modeled human UGT1A1 3D structure is consistent with genetic mutation data and provides a foundation for understanding the catalytic mechanism of vertebrate glucuronidation.

### Diversifying selection of vertebrate *Ugt *clusters

Enormous molecular diversity is required for the immune and nervous system function. In the adaptive immune system, positive molecular selection operates to increase the diversity of the *Ig*, *Mhc*, and *Tcr *genes [8,9,11,17,18,70]. In the CNS, adaptive molecular selection also operates to enhance the diversity of the *Pcdh *and olfactory receptor gene clusters [5,11,71]. Human UGT proteins glucuronidate numerous endogenous substrates including steroids and bile acids, as well as diverse xenobiotic chemicals such as environmental carcinogens and therapeutic drugs [20]. Gene duplication and birth-and-death evolution are major sources of UGT diversity in the vertebrate evolution (Fig. 1). We hypothesize that positive selection may be an additional factor to enhance the diversity of vertebrate *Ugt *genes.

We searched for positively selected sites in *Ugt *genes for various vertebrate species using the maximum-likelihood codeml program [72]. We ran three pairs of nested codeml models on the human, chimpanzee, rhesus monkey, baboon, dog, mouse, rat, chicken, and zebrafish clusters *a *and *b Ugt1 *genes, as well as the human, mouse, and rat *Ugt2 *genes to infer positively selected codon sites. The parameter estimates for the *Ugt *genes are shown in the Additional file 15. The positively selected ω+ sites in each repertoire are shown in the Additional file 16. Different vertebrate species have overlapping but distinct ω+ site profiles for the *Ugt1 *and *Ugt2 *genes, even between very closely-related lineages such as mice and rats (Additional file 16), suggesting that these *Ugt *genes in different species are evolved through different chemical environments.

**Figure 8:**
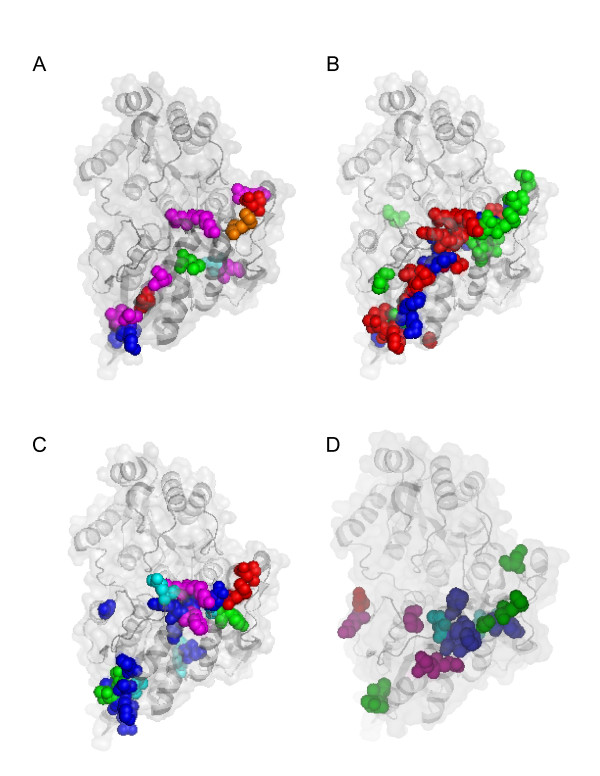
Site-specific K_A_/K_S _analysis of the vertebrate *Ugt1 *and *Ugt2 *clusters. Shown are positively selected ω+ sites mapped to the acceptor domain of a ribbon diagram of the UGT71G1 crystal structure [47]. (**A**) Positively selected sites in the primate and dog UGT1. The ω+ sites for the human only are highlighted in green; for the baboon only, in orange; for the rhesus monkey only, in cyan; for the dog only, in magenta; for the chimpanzee and dog only, in blue; and for all five species, in red. (**B**) Positively selected sites in the rodent UGT1. The ω+ sites for the mouse only are highlighted in blue; for the rat only, in green; and for both mouse and rat, in red. (**C**) Positively selected sites in the chicken and zebrafish UGT1. The ω+ sites for the chicken only are highlighted in blue; for the zebrafish cluster *Ugt1a *only, in magenta; for the zebrafish cluster *Ugt1b *only, in cyan; for both chicken and zebrafish cluster *Ugt1a*, in red; and for both zebrafish clusters *Ugt1a *and *Ugt1b*, in green. (**D**) Positively selected sites in the human, mouse, and rat UGT2. The ω+ sites for the human only are highlighted in magenta; for the mouse only, in cyan; for the rat only, in green; for the mouse and rat only, in blue; and for all three species, in red. The residues mapped are comparably numbered according to the sequence of UGT71G1 and are equivalent between UGT1 and UGT2, and among different vertebrate species.

Based on the structural alignment in the Figure 4A, we aligned the polypeptide sequence of UGT71G1 to those of the human (Additional file 17), chimpanzee (Additional file 18), rhesus monkey (Additional file 19), baboon (Additional file 20), dog (Additional file 21), mouse (Additional file 22), rat (Additional file 23), chicken (Additional file 24), and zebrafish clusters A (Additional file 25) and B (Additional file 26) UGT1 variable-exon-encoded polypeptides, as well as the corresponding human (Additional file 27), mouse (Additional file 28), and rat (Additional file 29) UGT2 proteins. We then mapped the positively-selected vertebrate UGT ω+ sites onto the crystal structure of UGT71G1 [47] on the basis of these alignments (Fig. 8). Interestingly, almost all positively selected sites are located within the four vertebrate UGT hypervariable regions and map to the acceptor-binding pocket of the UGT71G1 crystal structure. Thus, these residues may participate in the recognition of diverse acceptor molecules by vertebrate UGT proteins. This observation suggests that nature selection operates to increase the diversity of vertebrate UGT proteins.

### Evolution of multiple variable first exons and *UGT *diversity

We showed that the variable and constant organizations of *Ugt1*, *Gcnt2*, and *Ugt2a *clusters are vertebrate-specific (Figs. 1 and 3, and Additional file 10). In addition, these clusters are mainly subject to birth-and-death evolution instead of concerted evolution because there is no prevalent gene conversion (Additional file 6). Finally, nature selection at specific residues in four hypervariable regions in the UGT acceptor-binding domain increases their diversity for binding numerous environmental agents (Fig. 8 and Additional files 15 and 16). Interestingly, a recent human population genetic study found that diversified coding sites are more likely to be polymorphic than conserved sites [29].

In the vertebrate CNS, birth-and-death evolution of *Pcdh *variable exon arrays and positive selection on their specific ectodomain codons contribute to the staggering diversity required for neuronal connectivity [3-7]. In the vertebrate adaptive immune system, DNA rearrangement of variable and constant gene segments in the *Ig *and *Tcr *clusters, in conjunction with birth-and-death evolution and positive selection, generate unlimited diversity. Highly polymorphic *Mhc *genes also undergo birth-and-death evolution and overdominant selection [11,19]. In particular, positive selection at hypervariable regions or CDRs of IG, TCR, and MHC proteins enhances their diversity for binding numerous antigens [8-10,17,18]. In the vertebrate detoxification system, UGT proteins recognize a myriad of hydrophobic aglycone molecules and each UGT has distinct but broad overlapping substrate specificities [20,49]. Similar to the nervous and immune systems, two factors contribute to the diversity of UGT proteins for defense against small chemicals. The duplication of *Ugt1 *variable exons and the entire *Ugt2 *genes increases the number of distinct vertebrate UGT proteins (Fig. 1 and Additional file 10). In addition, the diversified residues in hypervariable regions through positive selection contribute to the binding specificity of each vertebrate UGT protein for a large set of distinct aglycones (Figs. 7 and 8; Additional files 15 and 16). Thus, our results reveal an intriguing similarity of diversification mechanisms between vertebrate nervous, immune, and chemical defense systems.

## Conclusion

The ability of UGT enzymes to glucuronidate numerous endobiotics and xenobiotics is conferred by their unusual genomic organization and structure diversity. Each *Ugt1 *variable exon is preceded by a distinct promoter. A highly conserved DNA motif located at about the same position upstream from each variable exon is likely to play an important role in regulating *Ugt1 *gene expression (Additional file 5). The combination of specific promoter activation and alternative *cis*-splicing of a variable exon to constant exons determines their tissue-specific expression. Comparative modeling of all UGT proteins suggests that each has di-domain Rossmann folds with a hydrophobic acceptor-binding pocket located within the N-terminal domain. Maximum-likelihood analysis of nt substitution patterns identified positively selected residues located in four hypervariable regions of the N-terminal domain (Fig. 7). Structural modeling suggests that these hypervariable regions form the hydrophobic acceptor-binding pocket (Fig. 6). Therefore, highly diversified residues in the acceptor-binding pocket could enable different UGT1 proteins to have distinct glucuronidation profiles for a large repertoire of environmental agents. Our comparative sequences analysis and homologous modeling shed light on the evolution of multiple variable exons and provide a framework for future structural and biochemical characterization of the vertebrate UGT proteins.

## Methods

### Comparative sequence and phylogenetic analyses

The vertebrate *Ugt1 *and *Gcnt2*, and mammalian *Ugt2 *genomic sequences were identified by iterative BLAST searches of the GenBank databases. The finished sequences were downloaded and analyzed as previously described [1,5]. The human gene nomenclature was following the recommendation of the HUGO committee. To ensure the accuracy, each nt was checked with the trace files from the TraceDB by using the Sequencher program. The sequences were analyzed for gene conversion by using the Geneconv program with default parameters [41]. Similar to previous convention [4], only sequence elements greater than 95 nt in length shared among paralogs are shown. The variable *Ugt1 *and *Gcnt2*, and the full-length *Ugt2 *coding sequences were translated and the resulting polypeptides were aligned by using the GCG package. The promoter motifs were identified by the Gibbs sampler [73] and the graphic representations were generated by the Weblogo [74]. Phylogenetic trees were reconstructed by using the neighbor-joining algorithm in the ClustalW package. Gaps in the alignment were treated as missing during the tree construction. The robustness of the tree partitions was evaluated by bootstrap analyses.

### Homologous modeling of UGT structures and molecular docking of substrates

We predicted the UGT1A1 secondary structure profile by using the neural network programs PSIPRED [75] and NNPREDICT [76], and aligned it to the structural alignment of known bacterial and plant GT-B crystal structures by using hidden Markov models (HMM) with manual adjustments [77]. We then modeled the structure of the human UGT1A1 by using the SWISS-MODEL [78]. The stereochemical quality of the structural model was evaluated with ANOLEA atomic mean force potential [79], GROMOS empirical force field energy, Verify3D profile [80], and the PROCHECK [81] programs. The modeled human UGT1A1 structure was refined by iterative modeling until there is no major difference in the active site between structural assessments of the model and the template [35]. In the final optimized UGT1A1 structure, dihedral angles of 331 residues were located in most favored regions of the Ramachandran plot, 53 residues in additional allowed regions, and 8 residues in generously allowed regions. We also modeled each of the 19 members of the human UGT1 and UGT2 families.

We modeled the UDPGA and bilirubin binding of UGT1A1 by using the molecular docking program GOLD (Genetic Optimization for Ligand Docking) [63] with default genetic algorithm parameters. The set up of the human UGT1A1 protein was according to the GOLD program manual. The UDPGA and bilirubin were downloaded from the PubChem Compound database. The UDPGA binding was modeled according to the cocrystal structure of UGT71G1 with the donor substrate. The bilirubin binding was modeled by seeding the atom NE2 of the residue H39 with a radius of 10 Å. GOLDscore was used to identify the lowest energy docking results. The hydrogen bonds and van der Waals interactions between ligands and UGT1A1 were analyzed to identify the optimal binding mode. The four hypervariable regions in the acceptor-binding domain were identified by multiple sequence alignment of all 91 vertebrate UGT1 variable polypeptides and the corresponding regions of 35 UGT2 proteins in conjunction with analyzing patterns of nt substitutions by the codeml program (see below).

### Site-specific *K*_A_/*K*_S _analysis

We used the maximum-likelihood codeml program of the PAML package (v3.15) [72] to predict codon sites under positive selection. The estimation of positively selected sites was performed as previously described [5]. Briefly, a set of 91 vertebrate *Ugt1 *variable exon sequences was translated and the resulting polypeptides were aligned with the N-terminal signal peptide removed. For the mammalian *Ugt2 *genes, a set of 31 full-length *Ugt2 *was aligned with both the N-terminal signal peptide and C-terminal transmembrane segment removed. The corresponding nt alignment was built by using RevTrans and separated into 10 *Ugt1 *(human, chimpanzee, rhesus monkey, baboon, dog, mouse, rat, chicken, and zebrafish clusters *a *and *b*) and 3 *Ugt2 *(human, mouse, and rat) groups. For each of these 13 groups, we first ran the model M0 of the codeml program with a nt neighbor-joining tree to obtain a K_S_-derived tree. By definition, the branches of the K_S _tree are about three times longer than those of the nt tree. However, almost all of the K_S _values are <1, suggesting that synonymous substitutions are not saturated among these UGT paralogs. We then used this tree to run three nested pairs of codeml random-sites models: M0 vs. M3; M1a vs. M2a; and M7 vs. M8. Because iterative estimations of ω values by both M2a and M8 are susceptible to local optima, we ran these models with three different initial ω values (0.03, 0.8, and 3.14) and presented only those results with the highest likelihood. We mapped the positively selected ω+ sites to the crystal structure of UGT71G1 (PDB accession code 2acv). The ω+ sites were defined as diversified residues estimated to be under positive selection with a posterior probability of >0.9 by one codeml model (M2a, M3, or M8), and >0.5 by at least one other model [5,71].

## Authors' contributions

CL and QW performed experiments and analyzed data. QW conceived of the study and wrote the manuscript. All authors read and approved the final manuscript.

## Supplementary Material

Additional file 1 Vertebrate *Ugt1 *genes. The mRNA and protein sequences for 65 new vertebrate *Ugt1 *genes are shown in the FASTA format. Click here for file

Additional file 2 Vertebrate *Gcnt2 *genes. The mRNA and protein sequences for 16 new and 9 known vertebrate *Gcnt2 *genes are shown in the FASTA format. Click here for file

Additional file 3 Vertebrate *Ugt2a *and the rat *Ugt2b39 *genes. The mRNA and protein sequences for 16 vertebrate *Ugt2a *and the rat *Ugt2b39 *genes are shown in the FASTA format. Click here for file

Additional file 4 Alignment of vertebrate UGT1 constant polypeptides with conserved residues highlighted. The predicted 17-aa transmembrane segment across the ER is marked by a line below. Identical conserved residues are shown in black box shade, similar conserved residues in grew shade, and nonidentical residues are left with a white background. Abbreviations for species: MMs, *Mus musculus*; RN, *Rattus norvegicus*; MMa, *Macaca mulatta*; PA, *Papio anubis*; HS, *Homo sapiens*; PT, *Pan troglodytes*; CF, *Canis familiaris*; BT, *Bos taurus*; MD, *Monodelphis domestica*; GG, *Gallus gallus*; XT, *Xenopus tropicalis*; and DR, *Danio rerio*, which has two highly similar constant polypeptides each is 6-aa shorter than in other vertebrate species. Click here for file

Additional file 5 Alignment of conserved sequence motifs upstream of the human (h), chimpanzee (c), rhesus monkey (*Macaca Mulatta *[mma]), baboon (b), dog (d), mouse (m), rat (r), chicken (*Gallus gallus *[gg]), and zebrafish (z) *Ugt1 *variable exons. Shown are the conserved sequence motifs identified by the Gibbs sampler [73] in the 500 nt regions upstream of the translation start codon (indicated by the negative numbers flanking the motifs) of each *Ugt1 *variable exon in the mammalian and avian bilirubin (**A**) and phenol (**B**) groups as well as in the zebrafish *Ugt1a *and *Ugt1b *clusters (**E**). The probability of the motif element is shown within parentheses on the right. The graphic sequence log representations [74] of the corresponding motifs are shown in panels (**C**), (**D**), and (**F**), respectively. The height of symbols corresponds to the relative frequency of each nt. Click here for file

Additional file 6 Gene conversion events in vertebrate multiple-variable-exon gene clusters. BC KA P-value: Bonferroni-corrected Karlin-Altschul P value. Only lengths > 95 nt are shown. Click here for file

Additional file 7 Alignment of vertebrate GCNT2 variable polypeptides (A, B, and C) with conserved residues highlighted. The predicted transmembrane segment is marked by a line below. The six identical cysteine residues are marked by asterisks below. Identical conserved residues are shown in black box shade, similar conserved residues in grew shade, and nonidentical residues are left with a white background. Abbreviations for species: GG, *Gallus gallus*; XT, *Xenopus tropicalis*; MMs, *Mus musculus*; RN, *Rattus norvegicus*; PT, *Pan troglodytes*; HS, *Homo sapiens*; MMa, *Macaca mulatta*; CF, *Canis familiaris*; MD, *Monodelphis domestica*; and DR, *Danio rerio*. Click here for file

Additional file 8 Phylogenetic tree of the human (h), chimpanzee (c), rhesus monkey (*Macaca mulatta *[mma]), dog (d), mouse (m), rat (r), opossum (o), chicken (*Gallus gallus *[gg]), frog (f), and zebrafish (*Danio rerio *[dr]) *Gcnt2 *clusters. The tree branches are labeled with the percentage support for that partition based on 1,000 bootstrap replicates. Only bootstrap values of >50% on major branches are shown. The scale bar equals a distance of 0.1. Click here for file

Additional file 9 Alignment of the vertebrate GCNT2 constant polypeptides with conserved residues highlighted. The three identical cysteine residues are marked by asterisks below. Identical conserved residues are shown in black box shade, similar conserved residues in grew shade, and nonidentical residues are left with a white background. Abbreviations for species: HS, *Homo sapiens*; PT, *Pan troglodytes*; MMa, *Macaca mulatta*; CF, *Canis familiaris*; MMs, *Mus musculus*; RN, *Rattus norvegicus*; MD, *Monodelphis domestica*; GG, *Gallus gallus*; XT, *Xenopus tropicalis*; and DR, *Danio rerio*. Click here for file

Additional file 10 Comparison of the human (h) (**A**), mouse (m) (**B**), and rat (r) (**C**) *Ugt2 *clusters with transcription direction marked by an arrow above each functional gene (pseudogenes are not shown), and their conserved genomic organization (**D**) (representing both *Ugt2a *and *Ugt2b*) with exon length indicated. (**E**) The organization of the zebrafish (z) *Ugt2a *gene cluster with exon length indicated. The exons of the *Ugt2a *are represented by vertical colored bars: (green) variable exon; (magenta) first constant exon; (purple) second constant exon; (teal) third constant exon; (blue) fourth constant exon; and (light blue) fifth constant exon. The corresponding six exons in individual *Ugt2b *genes are similarly colored. The approximate length of each cluster is shown below the corresponding panels. The chromosomal location is indicated on the right for each cluster. Var, variable; Con, constant; and kb, kilobase pairs. Click here for file

Additional file 11 Phylogenetic tree of the human (h), chimpanzee (c), rhesus macaque (*Macaca mulatta *[mma]), dog (d), mouse (m), rat (r), and zebrafish (z) *Ugt2a *clusters. The major branches of the tree are labeled with the percentage support for that partition based on 1,000 bootstrap replicates. Only bootstrap values of >50% on major branches are shown. The scale bar equals a distance of 0.1. Click here for file

Additional file 12 Alignment of the vertebrate UGT2A variable polypeptides with conserved residues highlighted. The predicted signal peptides are indicated by a line below. Identical conserved residues are shown in black box shade, similar conserved residues in grew shade, and nonidentical residues are left with a white background. Abbreviations for species: DR, *Danio rerio*; MMs, *Mus musculus*; RN, *Rattus norvegicus*; PT, *Pan troglodytes*; HS, *Homo sapiens*; MMa, *Macaca mulatta*; and CF, *Canis familiaris*. Click here for file

Additional file 13 Alignment of the vertebrate UGT2A constant polypeptides with conserved residues highlighted. The predicted 17-aa transmembrane segment across the ER is marked by a line below. Identical conserved residues are shown in black box shade, similar conserved residues in grew shade, and nonidentical residues are left with a white background. Abbreviations for species: MMs, *Mus musculus*; RN, *Rattus norvegicus*; CF, *Canis familiaris*; PT, *Pan troglodytes*; HS, *Homo sapiens*; MMa, *Macaca mulatta*; and DR, *Danio rerio*. Click here for file

Additional file 14 Phylogenetic tree of the human (h), mouse (m), and rat (r) *Ugt2b *clusters. The major tree branches are labeled with the percentage support for that partition based on 1,000 bootstrap replicates. The scale bar equals a distance of 0.1. Click here for file

Additional file 15 Log-likelihood values and parameter estimates for human, chimpanzee, rhesus monkey, baboon, dog, mouse, rat, chicken, and zebrafish *Ugt1 *groups, and human, mouse, and rat *Ugt2 *groups. **Model^1 ^**Maximum-likelihood models implemented in the codeml program of the PAML package. M0, one-ratio; M1a, neutral; M2a, selection; M3, discrete; M7, β; M8, β+ω. **ℓ^2 ^**Estimated log-likelihood values by the codeml program. **κ^3 ^**Estimated transition/transversion rate ratio by the codeml program. **Estimation of Parameters^4 ^**ω = K_A_/K_S _nonsynonymous/synonymous rate ratio; p = proportion of sites for each site class. M0: one estimated ω for all sites; M1a: estimate p_0 _= proportion of sites with ω_0 _= 0, p_1 _= 1 - p_0_, proportion of sites with ω_1 _= 1; M2a: estimate p_0 _(ω_0 _= 0), p_1 _(ω_0 _= 1), and ω_2_, p_2 _= 1 - p_0 _- p_1_. M3: estimate p_0_, p_1_, ω_0_, ω_1_, and ω_2_; p_2 _= 1 - p_0 _- p_1_. M7: estimates p and q (parameters of β distribution of ω between 0 and 1). M8: same as M7 except additional site class where an estimated ω is allowed. **LRT**(2Δℓ)^5 ^Statistical likelihood ratio test; comparing the test statistic (2Δℓ) calculated from paired codeml models (M1a vs M2a; M0 vs M3; and M7 vs M8) with the critical value of chi-square asymptotic distribution with appropriate degrees of freedom (i.e. 2 d.f., 4 d.f., and 2 d.f., respectively). 2Δℓ and level of significance are shown for M2a, M3, and M8 models. **Positively Selected Sites^6 ^**Codon positions predicted to be under positive selection with a posterior probability >0.90 by one codeml model (M2a, M3, or M8), and >0.50 by at least one other model. Click here for file

Additional file 16 Summary information for *Ugt1 *and *Ugt2 *groups analyzed. **Tree length^1 ^**Measured as the number of nt substitutions along the tree per codon by the codeml program. **The ω+ sites^2 ^**Codon positions predicted to be under positive selection with a posterior probability >0.90 by one codeml model (M2a, M3, or M8), and >0.50 by at least one other model. For *Ugt1 *sequences, we used only the variable exons. For *Ugt2 *sequences, we used full-length sequences but excluded *Ugt2a2 *because it shares five constant exons with *Ugt2a1*. Click here for file

Additional file 17 Alignment of the protein sequences of UGT71G1 to those of the human (Additional file 17), chimpanzee (Additional file 18), rhesus monkey (Additional file 19), baboon (Additional file 20), dog (Additional file 21), mouse (Additional file 22), rat (Additional file 23), chicken (Additional file 24), and zebrafish clusters A (Additional file 25) and B (Additional file 26) variable UGT1 sequences, as well as the corresponding human (Additional file 27), mouse (Additional file 28), and rat (Additional file 29) UGT2 proteins. Each vertebrate UGT group was aligned by ClustalW and then aligned with UGT71G1 according to the structural alignment shown in the Figure 4A. The ω+ sites predicted to be subject to positive selection with a posterior probability >0.90 by one model and >0.5 by at least one other model are highlighted in red. Click here for file

Additional file 18 Alignment of the protein sequences of UGT71G1 to those of the human (Additional file 17), chimpanzee (Additional file 18), rhesus monkey (Additional file 19), baboon (Additional file 20), dog (Additional file 21), mouse (Additional file 22), rat (Additional file 23), chicken (Additional file 24), and zebrafish clusters A (Additional file 25) and B (Additional file 26) variable UGT1 sequences, as well as the corresponding human (Additional file 27), mouse (Additional file 28), and rat (Additional file 29) UGT2 proteins. Each vertebrate UGT group was aligned by ClustalW and then aligned with UGT71G1 according to the structural alignment shown in the Figure 4A. The ω+ sites predicted to be subject to positive selection with a posterior probability >0.90 by one model and >0.5 by at least one other model are highlighted in red. Click here for file

Additional file 19 Alignment of the protein sequences of UGT71G1 to those of the human (Additional file 17), chimpanzee (Additional file 18), rhesus monkey (Additional file 19), baboon (Additional file 20), dog (Additional file 21), mouse (Additional file 22), rat (Additional file 23), chicken (Additional file 24), and zebrafish clusters A (Additional file 25) and B (Additional file 26) variable UGT1 sequences, as well as the corresponding human (Additional file 27), mouse (Additional file 28), and rat (Additional file 29) UGT2 proteins. Each vertebrate UGT group was aligned by ClustalW and then aligned with UGT71G1 according to the structural alignment shown in the Figure 4A. The ω+ sites predicted to be subject to positive selection with a posterior probability >0.90 by one model and >0.5 by at least one other model are highlighted in red. Click here for file

Additional file 20 Alignment of the protein sequences of UGT71G1 to those of the human (Additional file 17), chimpanzee (Additional file 18), rhesus monkey (Additional file 19), baboon (Additional file 20), dog (Additional file 21), mouse (Additional file 22), rat (Additional file 23), chicken (Additional file 24), and zebrafish clusters A (Additional file 25) and B (Additional file 26) variable UGT1 sequences, as well as the corresponding human (Additional file 27), mouse (Additional file 28), and rat (Additional file 29) UGT2 proteins. Each vertebrate UGT group was aligned by ClustalW and then aligned with UGT71G1 according to the structural alignment shown in the Figure 4A. The ω+ sites predicted to be subject to positive selection with a posterior probability >0.90 by one model and >0.5 by at least one other model are highlighted in red. Click here for file

Additional file 21 Alignment of the protein sequences of UGT71G1 to those of the human (Additional file 17), chimpanzee (Additional file 18), rhesus monkey (Additional file 19), baboon (Additional file 20), dog (Additional file 21), mouse (Additional file 22), rat (Additional file 23), chicken (Additional file 24), and zebrafish clusters A (Additional file 25) and B (Additional file 26) variable UGT1 sequences, as well as the corresponding human (Additional file 27), mouse (Additional file 28), and rat (Additional file 29) UGT2 proteins. Each vertebrate UGT group was aligned by ClustalW and then aligned with UGT71G1 according to the structural alignment shown in the Figure 4A. The ω+ sites predicted to be subject to positive selection with a posterior probability >0.90 by one model and >0.5 by at least one other model are highlighted in red. Click here for file

Additional file 22 Alignment of the protein sequences of UGT71G1 to those of the human (Additional file 17), chimpanzee (Additional file 18), rhesus monkey (Additional file 19), baboon (Additional file 20), dog (Additional file 21), mouse (Additional file 22), rat (Additional file 23), chicken (Additional file 24), and zebrafish clusters A (Additional file 25) and B (Additional file 26) variable UGT1 sequences, as well as the corresponding human (Additional file 27), mouse (Additional file 28), and rat (Additional file 29) UGT2 proteins. Each vertebrate UGT group was aligned by ClustalW and then aligned with UGT71G1 according to the structural alignment shown in the Figure 4A. The ω+ sites predicted to be subject to positive selection with a posterior probability >0.90 by one model and >0.5 by at least one other model are highlighted in red. Click here for file

Additional file 23 Alignment of the protein sequences of UGT71G1 to those of the human (Additional file 17), chimpanzee (Additional file 18), rhesus monkey (Additional file 19), baboon (Additional file 20), dog (Additional file 21), mouse (Additional file 22), rat (Additional file 23), chicken (Additional file 24), and zebrafish clusters A (Additional file 25) and B (Additional file 26) variable UGT1 sequences, as well as the corresponding human (Additional file 27), mouse (Additional file 28), and rat (Additional file 29) UGT2 proteins. Each vertebrate UGT group was aligned by ClustalW and then aligned with UGT71G1 according to the structural alignment shown in the Figure 4A. The ω+ sites predicted to be subject to positive selection with a posterior probability >0.90 by one model and >0.5 by at least one other model are highlighted in red. Click here for file

Additional file 24 Alignment of the protein sequences of UGT71G1 to those of the human (Additional file 17), chimpanzee (Additional file 18), rhesus monkey (Additional file 19), baboon (Additional file 20), dog (Additional file 21), mouse (Additional file 22), rat (Additional file 23), chicken (Additional file 24), and zebrafish clusters A (Additional file 25) and B (Additional file 26) variable UGT1 sequences, as well as the corresponding human (Additional file 27), mouse (Additional file 28), and rat (Additional file 29) UGT2 proteins. Each vertebrate UGT group was aligned by ClustalW and then aligned with UGT71G1 according to the structural alignment shown in the Figure 4A. The ω+ sites predicted to be subject to positive selection with a posterior probability >0.90 by one model and >0.5 by at least one other model are highlighted in red. Click here for file

Additional file 25 Alignment of the protein sequences of UGT71G1 to those of the human (Additional file 17), chimpanzee (Additional file 18), rhesus monkey (Additional file 19), baboon (Additional file 20), dog (Additional file 21), mouse (Additional file 22), rat (Additional file 23), chicken (Additional file 24), and zebrafish clusters A (Additional file 25) and B (Additional file 26) variable UGT1 sequences, as well as the corresponding human (Additional file 27), mouse (Additional file 28), and rat (Additional file 29) UGT2 proteins. Each vertebrate UGT group was aligned by ClustalW and then aligned with UGT71G1 according to the structural alignment shown in the Figure 4A. The ω+ sites predicted to be subject to positive selection with a posterior probability >0.90 by one model and >0.5 by at least one other model are highlighted in red. Click here for file

Additional file 26 Alignment of the protein sequences of UGT71G1 to those of the human (Additional file 17), chimpanzee (Additional file 18), rhesus monkey (Additional file 19), baboon (Additional file 20), dog (Additional file 21), mouse (Additional file 22), rat (Additional file 23), chicken (Additional file 24), and zebrafish clusters A (Additional file 25) and B (Additional file 26) variable UGT1 sequences, as well as the corresponding human (Additional file 27), mouse (Additional file 28), and rat (Additional file 29) UGT2 proteins. Each vertebrate UGT group was aligned by ClustalW and then aligned with UGT71G1 according to the structural alignment shown in the Figure 4A. The ω+ sites predicted to be subject to positive selection with a posterior probability >0.90 by one model and >0.5 by at least one other model are highlighted in red. Click here for file

Additional file 27 Alignment of the protein sequences of UGT71G1 to those of the human (Additional file 17), chimpanzee (Additional file 18), rhesus monkey (Additional file 19), baboon (Additional file 20), dog (Additional file 21), mouse (Additional file 22), rat (Additional file 23), chicken (Additional file 24), and zebrafish clusters A (Additional file 25) and B (Additional file 26) variable UGT1 sequences, as well as the corresponding human (Additional file 27), mouse (Additional file 28), and rat (Additional file 29) UGT2 proteins. Each vertebrate UGT group was aligned by ClustalW and then aligned with UGT71G1 according to the structural alignment shown in the Figure 4A. The ω+ sites predicted to be subject to positive selection with a posterior probability >0.90 by one model and >0.5 by at least one other model are highlighted in red. Click here for file

Additional file 28 Alignment of the protein sequences of UGT71G1 to those of the human (Additional file 17), chimpanzee (Additional file 18), rhesus monkey (Additional file 19), baboon (Additional file 20), dog (Additional file 21), mouse (Additional file 22), rat (Additional file 23), chicken (Additional file 24), and zebrafish clusters A (Additional file 25) and B (Additional file 26) variable UGT1 sequences, as well as the corresponding human (Additional file 27), mouse (Additional file 28), and rat (Additional file 29) UGT2 proteins. Each vertebrate UGT group was aligned by ClustalW and then aligned with UGT71G1 according to the structural alignment shown in the Figure 4A. The ω+ sites predicted to be subject to positive selection with a posterior probability >0.90 by one model and >0.5 by at least one other model are highlighted in red. Click here for file

Additional file 29 Alignment of the protein sequences of UGT71G1 to those of the human (Additional file 17), chimpanzee (Additional file 18), rhesus monkey (Additional file 19), baboon (Additional file 20), dog (Additional file 21), mouse (Additional file 22), rat (Additional file 23), chicken (Additional file 24), and zebrafish clusters A (Additional file 25) and B (Additional file 26) variable UGT1 sequences, as well as the corresponding human (Additional file 27), mouse (Additional file 28), and rat (Additional file 29) UGT2 proteins. Each vertebrate UGT group was aligned by ClustalW and then aligned with UGT71G1 according to the structural alignment shown in the Figure 4A. The ω+ sites predicted to be subject to positive selection with a posterior probability >0.90 by one model and >0.5 by at least one other model are highlighted in red. Click here for file
