# Supplementary material for: Adaptive evolution of multiple-variable exons and structural diversity of drug-metabolizing enzymes
Source: BMC Evol Biol. 2007 May 2;7:69. doi: 10.1186/1471-2148-7-69 (PMC1885805; doi:10.1186/1471-2148-7-69)
Supplement: Additional file 4 — Alignment of vertebrate UGT1 constant polypeptides with conserved residues highlighted. The predicted 17-aa transmembrane segment across the ER is marked by a line below. Identical conserved residues are shown in black box shade, similar conserved residues in grew shade, and nonidentical residues are left with a white background. Abbreviations for species: MMs, Mus musculus; RN, Rattus norvegicus; MMa, Macaca mulatta; PA, Papio anubis; HS, Homo sapiens; PT, Pan troglodytes; CF, Canis familiaris; BT, Bos taurus; MD, Monodelphis domestica; GG, Gallus gallus; XT, Xenopus tropicalis; and DR, Danio rerio, which has two highly similar constant polypeptides each is 6-aa shorter than in other vertebrate species. [file 1471-2148-7-69-S4.pdf]

```
MMs    1 EFEAYVNASGEHGIVVFSLGSMVSEIPEKKAMEIAEALGRIPQTVLWRYTGTRPSNLAKN
RN     1 EFEAYVNASGEHGIVVFSLGSMVSEIPEKKAMEIAEALGRIPQTVLWRYTGTRPSNLAKN
MMa    1 EFEAYINASGEHGIVVFSLGSMVAEIPEKKAMAIADALGKIPQTVLWRYTGTPPSNLANN
PA     1 EFEAYINASGEHGIVVFSLGSMVAEIPEKKAMAIADALGKIPQTVLWRYTGTPPSNLANN
HS     1 EFEAYINASGEHGIVVFSLGSMVSEIPEKKAMAIADALGKIPQTVLWRYTGTRPSNLANN
PT     1 EFEAYINASGEHGIVVFSLGSMVSEIPEKKAMAIADALGKIPQTVLWRYTGTRPSNLANN
CF     1 EFEAYVNASGEHGIVVFSLGSMVSDIPEKKAMEIADALGKIPQTVLWRYTGTPPPNLSKN
BT     1 EFEAYVNASGEHGIVVFSLGSMVSEIPEQKAMEIADALGKIPQTVLWRYTGTPPPNLAKN
MD     1 EFEAYVNASGEHGVVVFSLGSMVSEIPMAKAMEIAEALGTIPQTVLWRYTGKPPSNLAKN
GG     1 EFEAIVNASGEHGIVVFSLGSMVSEIPMKKAMEIADALGSVPQTVLWRYTGEVPPNLPKN
XT     1 EFEKLVNSSGEHGFVVFSLGSMVSEIPMNKAMDIAEALKSIPQKVFWRYTGKAPPNLGEN
DR1a   1 ELEEFVNGSGEHGFVVFTLGSMVSQLPEAKAREFFEAFRQIPQRVLWRYTGPVPENAPKN
DR1b   1 EVEEFVNGSGEHGIVVFSLGSLVSSMPKEKADIFFKAFSMIPQRVLWRYTGEIPNNVPEN

MMs   61 TILVKWLPQNDLLGHPKTRAFITHSGSHGIYEGICNGVPMVMMPLFGDQMDNAKRMETRG
RN    61 TILVKWLPQNDLLGHPKARAFITHSGSHGIYEGICNGVPMVMMPLFGDQMDNAKRMETRG
MMa   61 TILVKWLPQNDLLGHPMTRAFITHAGSHGIYEGICNGVPMVMMPLFGDQMDNAKRMETKG
PA    61 TILVKWLPQNDLLGHPMTRAFITHAGSHGIYEGICNGVPMVMMPLFGDQMDNAKRMETKG
HS    61 TILVKWLPQNDLLGHPMTRAFITHAGSHGVYESICNGVPMVMMPLFGDQMDNAKRMETKG
PT    61 TILVKWLPQNDLLGHPMTRAFITHAGSHGVYESICNGVPMVMMPLFGDQMDNAKRMETKG
CF    61 TILVKWLPQNDLLGHPKARAFITHSGSHGIYEGICNGVPMVMLPLFGDQMDNAKRMETRG
BT    61 TKLVKWLPQNDLLGHPKTRAFITHSGSHGIYEGICNGVPMVMMPLFGDQMDNAKRMETRG
MD    61 TKLVKWLPQNDLLAHPKARAFITHAGSHGIYEGICNGVPMVLMPLFGDQMDNAKRMESRG
GG    61 VKLVKWLPQNDLLAHPKTRAFITHGGSHGVYEGICNAVPMVLMPLFGDQMDNAKRVESRG
XT    61 THLVKWLPQNDLLAHPKARAFITHAGSHGIYEGICNAVPMVMMPLFGDQMDNAKRIESRG
DR1a  61 VKLMKWLPQNDLLGHPKVRAFVTHGGSHGIYEGICNGVPMVMLPLFGDQGDNAQRLVSRG
DR1b  61 VKLMKWLPQNDLLGHPKARAFITHGGTHGIYEGICHGVPMVMLPLFGDQADNVHRVATRG

MMs  121 AGVTLNVLEMTADDLENALKTVINNKSYKENIMRLSSLHKDRPIEPLDLAVFWVEYVMRH
RN   121 AGVTLNVLEMTADDLENALKTVINNKSYKENIMRLSSLHKDRPIEPLDLAVFWVEYVMRH
MMa  121 AGVTLNVLEMTSEDLENALKAVINDKSYKENIMHLSSLHKDRPVEPLDLAVFWVEFVMRH
PA   121 AGVTLNVLEMTSEDLENALKAVINDKSYKENIMHLSSLHKDRPVEPLDLAVFWVEFVMRH
HS   121 AGVTLNVLEMTSEDLENALKAVINDKSYKENIMRLSSLHKDRPVEPLDLAVFWVEFVMRH
PT   121 AGVTLNVLEMTSEDLENALKAVINDKSYKENIMRLSSLHKDRPVEPLDLAVFWVEFVMRH
CF   121 AGVTLNVLEMTSADLANALKAVINDKSYKENIMHLSRLHKDRPIEPLDLAVFWVEFVMRH
BT   121 AGVTLNVLEMSSEDLEKALKAVINEKTYKENIMRLSRLHKDRPIEPLDLAVFWVEFVMRH
MD   121 AGVILNVLDMTSSDLSKALKTVINDKSYKENIMRLSALHKDRPIDPLNLAVFWVEFVMRH
GG   121 AGLTLNILEMTSKDISDALKAVINDKKYKENIQRLSDLHLDRPIHPLDLAVHWVEFVMRH
XT   121 AGLTLNVLHMTPEDLSNAVRAVINNPVYKENIQRMSSLHLDRPIHPLDLAVHWVEFVMRH
DR1a 121 VAESLTIYDVTSEKLLVALKKVINDKSYKEKMMKLSAIHRDRPIEPLDLAVFWTEFVMRH
DR1b 121 VGVILSIHDITVETLLDALNSVINNSSYKQKMQKLSAIHNDRPIQPLDLAVFWTEFVMRH

MMs  181 KGAPHLRPAAHDLTWYQYHSLDVIGFLLAIVLTVVFIVFKCCAYGCRKCFGGKGRVKKSHKSKTH
RN   181 KGAPHLRPAAHDLTWYQYHSLDVIGFLLAIVLTVVFIVYKSCAYGCRKCFGGKGRVKKSHKSKTH
MMa  181 KGAPHLRPAAHDLTWYQYHSLDVIGFLLAIVLTVAFIAFKCCAYGYRKCFGKKGRVKKAHKSKTH
PA   181 KGAPHLRPAAHDLTWYQYHSLDVIGFLLAIVLTVAFIAFKCCAYGYRKCFGKKGRVKKAHKSKTH
HS   181 KGAPHLRPAAHDLTWYQYHSLDVIGFLLAVVLTVAFITFKCCAYGYRKCLGKKGRVKKAHKSKTH
PT   181 KGAPHLRPAAHDLTWYQYHSLDVIGFLLAVVLTVAFITFKCCAYGYRKCFGKKGRVKKAHKSKTH
CF   181 KGAPHLRPAAHDLTWYQYHSLDVIGFLLAVVLGVVFITYKCCAFGCRKCFGKKGRVKKPHKSKAH
BT   181 KGASHLRPAAHDLTWYQYHSLDVIGFLLAVTLTVIFITFKACAFAFRKCFGKKERVKKSHKSKTH
MD   181 KGAPHLRAAAHDLNWIQYHSLDVIAFLLAIVLTVVLIIVKSCKFCFRKCFGKKGKAKKSTKPKSH
GG   181 KGAPHLRPAAHDLNWIQYHSLDVFAFLLAVVLLSLFISVKCCMFCCRRCCFKKGRKSKSGKSKTH
XT   181 KGAPHLRPAAHDLNWFQYYSLDVIGFLLAVLLTALFISLKCCTFVFRR.CFKRNSK.KQRKSKSQ
DR1a 181 KGAEHLRPAAHDLNWIQYHSLDVIGFLLLILLTVIFVTVKSCMFCFRKCFKKSQKKKKA~~~~~~
DR1b 181 KGADHLRPAAHELNWLQYHSLDVIGFMLLIVLIVTLAMLKCCSLCWRRCCRKTQKRKED~~~~~~
Transmembrane segment          -----------------
```
