# Supplementary material for: Adaptive evolution of multiple-variable exons and structural diversity of drug-metabolizing enzymes
Source: BMC Evol Biol. 2007 May 2;7:69. doi: 10.1186/1471-2148-7-69 (PMC1885805; doi:10.1186/1471-2148-7-69)

A

ha1	-231	agaat GTGATTTGAGTATGAAATTCC agcca	-211	(1.00)
ha3	-314	gtgac ATGAAATGGGTGTTCACAGCC ttgtg	-294	(1.00)
ha4	-314	gtgac ATGAAATGGGTGTCCACAGCC ttgtg	-294	(1.00)
ha5	-313	gtgac ATGAAATGGGGGTTCACAGCC ttgtg	-293	(1.00)
ca1	-229	agaat GTGATTTGAGTATGAAATTCC agcca	-209	(1.00)
ca2	-315	gtgac ATGAAATGGGTTTCCACAGGC ctttg	-295	(1.00)
ca3	-314	gtgac ATGAAATGGGTGTTCACAGCG ttgtg	-294	(1.00)
ca4	-314	gtgac ATGAAATGGGTGTCCACAGCC ttgtg	-294	(1.00)
mmaa1	-225	agaat GTGATTTAAGTATGAAATTCC agcca	-205	(1.00)
mmaa2	-314	gtgac ATGAAATGGGTGTTCACAGCC ttgta	-294	(1.00)
mmaa4a	-314	gtgac ATGAAATGGTTGTTCACAGCC ttgta	-294	(1.00)
mmaa4b	-318	gtgac ATGAAATGGGTGTTCACAGCC ttgtg	-298	(1.00)
mmaa5	-316	gtgac ATGAAATGGGTGTTCACAGCC ttgtg	-296	(1.00)
ba1	-225	agaat GTGATTTGAGTATGAAATTCC agcca	-205	(1.00)
ba2	-314	gtgac ATGAAATGGGTGTTCACAGCC ttgtg	-294	(1.00)
ba4	-318	gtgac ATGAAATGGGTGTTCACAGCC ttgtg	-298	(1.00)
ba5a	-317	gtgac ATGAAATGGGTGTTCACAGCC ttgtg	-297	(1.00)
ba5b	-312	gtgac ATGAAATGGGTGTTCACAGCC ttgtg	-292	(1.00)
da1	-81	tgagt ATGGATTGGTTCTTGCAACAC gaagt	-61	(1.00)
da2	-322	atcca GTGAATTGGGCATCCACAGCC ctgtt	-302	(1.00)
da3	-324	atcca GTGAATTGGGCATCCACAGCC ctgtt	-304	(1.00)
da4	-324	atcca GTGAATTGGGCATCCACAGCC ctgtt	-304	(1.00)
ma1	-353	gtgtt ATGCTTTGGCACTTTACGGTC ctttt	-333	(1.00)
ma2	-446	gcaga ATGACACTGATATGGAGAGAC agaat	-426	(1.00)
ma5	-292	atgga ATGAAATGGGTGTGCACAGCC atagt	-272	(1.00)
ra1	-348	atctt ATGCCTTGGCACTTTACAGTC ccttt	-328	(1.00)
ra2	-281	tggga AAGAAATGGGTGTTCAGCGCC ttatt	-261	(1.00)
ra4	-258	gcctt ATGAAATGAACTCTGACGGTC tctag	-238	(1.00)
ra6	-296	acagg ATGAAATGGGTGTACACAGCC ttgtt	-276	(1.00)
gga1	-107	atcaa AAGCTATGGTGTAGCACAGAT tgctg	-87	(1.00)
gga3	-107	atcaa AAGCTATGGTATAGCACAGAC tgctg	-87	(1.00)
gga5	-397	aaatc ATTCTAGAAGTCTTAACAGGC ctgag	-377	(1.00)
gga6	-87	ctcag ATTGCTTAAAAGTGCAGAGCT ggtgc	-67	(1.00)
gga7	-405	ctcct GTCAATTGGCACTTAATATTC tggtt	-385	(1.00)

B

ha6	-342	ttcgg GATAGAGAATTGGCAGGGGGT cctca	-322	(1.00)
ha7	-152	ttatg AGTAAATCATTGGCAGTGAAT gtgaa	-132	(1.00)
ha8	-155	ttatg AGTAAATCATTGGCAGTGAGT gtgat	-135	(1.00)
ha9	-150	ttttg GGTAAATCATTGTCAGTGACT gattt	-130	(1.00)
ha10	-154	ttatg AGTAAATCATTGGCAGTGAGT gtgat	-134	(1.00)
ca6	-342	ttcgg GATAGAGAATTGGCAGGGGGT cctca	-322	(1.00)
ca7	-151	ttatg AGTAAATCATTGGCAGTGAAT gtgaa	-131	(1.00)
ca8	-156	ttatg AGTAAATCATTGGCAGTGAGT gtgat	-136	(1.00)
ca9	-149	ttttg GGTAAATCATTGTCAGTGACT gattt	-129	(1.00)
ca10	-154	ttatg AGTAAATCATTGGCAGTGAGT gtgat	-134	(1.00)
mmaa6	-343	ttcgg GATAGAGAATTGGCAGGGGGT ctcca	-323	(1.00)
mmaa7	-155	ttatg AGTAAATCACTGGCAGTGAAT gtgca	-135	(1.00)
mmaa8	-163	ttagg AGTAAATCATTGGCAGTGAGT atgat	-143	(1.00)
mmaa9	-156	ttttg AGTAAATCATTGTCAGTGACT gattt	-136	(1.00)
mmaa10	-143	ttatg AGTAAATCATTGGCAGTGAGT gtgat	-123	(1.00)
ba6	-343	ttcgg GATAGAGAATTGGCAGGGGGT ctcca	-323	(1.00)
ba7	-157	ttatg AGTAAATCACTGGCAGTGAAT gtgca	-137	(1.00)
ba8	-156	ttagg AGTAAATCATTGGCAGTGAGT atgat	-136	(1.00)
ba9	-165	ttttg AGTAAATCATTGTCAGTGACT gattt	-145	(1.00)
ba10	-143	ttatg AGTAAATCATTGGCAGTGAGT gtgat	-123	(1.00)
da6	-211	gataa GGGACCTCATTGGCTCGAGAT ggcta	-191	(1.00)
da7	-164	ttagg AGGGCATCACTGGCAATGAGT gtagt	-144	(1.00)
da8	-161	ttagg AGTGCATCACTGGCAATGAGT gtagt	-141	(1.00)
da9	-161	ttagg AGTGCATCACTGGCAATGAGT gtagt	-141	(1.00)
da10	-162	ttagg AGGGCATCACTGGCAATGAGT gtagt	-142	(1.00)
da11	-164	ttagg AGGGCATCACTGGCAATAGGT gtagt	-144	(1.00)
ma7	-350	aaagt GTCAATGCATTGACAGAGAGC cattt	-330	(1.00)
ma9	-348	aaagt GTCAGTGCATTGACAGAGAGC cattt	-328	(1.00)
ma10	-122	tcctc AGTAAATCATTCTCCTAAACG gctgt	-102	(1.00)
ma11	-154	tcaaa AGTCAGTCATTGTCAGGGAGT gtact	-134	(1.00)
ma12	-150	tttta AGTAAATCATTAACCTGAATT taacg	-130	(1.00)
ma13	-150	tcttt TGTGAATAATTAACCTGAATT taaca	-130	(1.00)
ra7	-356	aaagt GTCAGAGCACTGACTGAGGGT cattt	-336	(1.00)
ra8	-137	tccca AGTAAATCATTGCCAGGAACT gctat	-117	(1.00)
ra9	-152	tcaaa AGTCAGTCATTGTCAGGGAGT gtact	-132	(1.00)
ra11	-151	ttttc AGTAAATCATTACCACTGAAT ttaac	-131	(1.00)
gga4	-457	tgcct ATCAAATCTCTGTCAATCATT acttc	-437	(1.00)
gga8	-92	gggca AAGGTATCATTAAAATGCAGT gccaa	-72	(1.00)
gga9	-412	ttgaa TGCAACTCTTTGAATGTAACT gtcca	-392	(1.00)
gga10	-255	taggc AGTAAATCATTAACTAGAGAA taatc	-235	(1.00)
gga11	-299	aaggc AGTAAATCATTAACTAGAACA taatc	-279	(1.00)
gga12	-305	aaggc AGTAAATCATTAACTAGAAGA taatc	-285	(1.00)
gga13	-287	agggc AGTAAATCATTAACTAGAAGA taatc	-267	(1.00)
gga14	-137	tgagc AGTAAAACATTAACTAGAATA taacc	-117	(1.00)

C

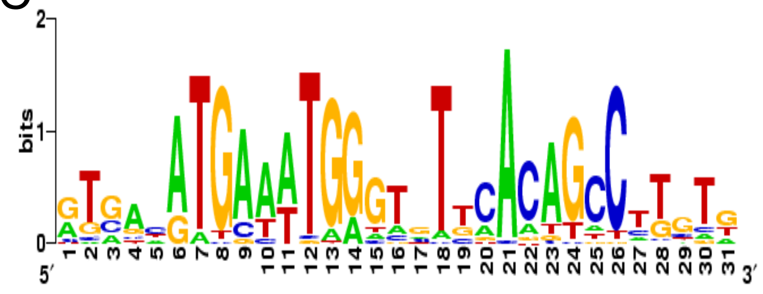

D

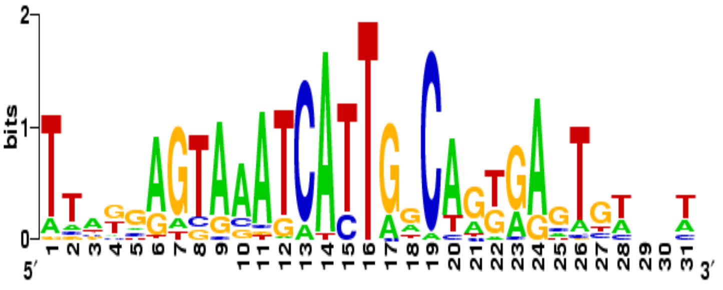

E

z1a1	-53	acatt ACACTACTGTTAATGTACTTC tgctc	-33	(1.00)
z1a2	-308	atggc TCCCTGCTGTTAGTTTATTGT tcatt	-288	(1.00)
z1a3	-380	gttta TAAATGCTGTAAAAGCATTGT tagta	-360	(1.00)
z1a4	-351	ttaaa TAAAGTCCTTAAATTAAATGT catgc	-331	(1.00)
z1a5	-378	acatt TCATTGCAGTAGATGAATTTC attaa	-358	(1.00)
z1a6	-357	gctct GTTTGTCTCATAATTCATTGT ctgca	-337	(1.00)
z1a7	-168	cagag ATTATTCTTTTGATGCCCTGC cttct	-148	(1.00)
z1b1	-310	tttgt GCATTTGTGTTAATGCATTTC attaa	-290	(1.00)
z1b2	-106	gcctt TTCTATCTGATAGTGAATTGT aaaag	-86	(1.00)
z1b3	-110	gtgga TTTTGTCTGATAGTGAATTGC gaaag	-90	(1.00)
z1b4	-110	ctgga TTTTGTCTGATAGTGAATTGC gaaag	-90	(1.00)
z1b5	-111	cgggg TTTTGTCTGATAATGAATTGC gaaag	-91	(1.00)
z1b7	-368	tacac TTTCGTCACTTGATTATCTGC atttt	-348	(1.00)

F

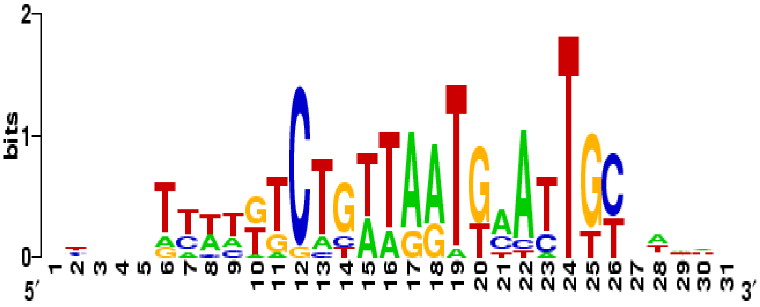

Supplement: Additional file 5 — Alignment of conserved sequence motifs upstream of the human (h), chimpanzee (c), rhesus monkey (Macaca Mulatta [mma]), baboon (b), dog (d), mouse (m), rat (r), chicken (Gallus gallus [gg]), and zebrafish (z) Ugt1 variable exons. Shown are the conserved sequence motifs identified by the Gibbs sampler [73] in the 500 nt regions upstream of the translation start codon (indicated by the negative numbers flanking the motifs) of each Ugt1 variable exon in the mammalian and avian bilirubin (A) and phenol (B) groups as well as in the zebrafish Ugt1a and Ugt1b clusters (E). The probability of the motif element is shown within parentheses on the right. The graphic sequence log representations [74] of the corresponding motifs are shown in panels (C), (D), and (F), respectively. The height of symbols corresponds to the relative frequency of each nt. [file 1471-2148-7-69-S5.pdf]
