# Supplementary material for: Adaptive evolution of multiple-variable exons and structural diversity of drug-metabolizing enzymes
Source: BMC Evol Biol. 2007 May 2;7:69. doi: 10.1186/1471-2148-7-69 (PMC1885805; doi:10.1186/1471-2148-7-69)
Supplement: Additional file 6 — Gene conversion events in vertebrate multiple-variable-exon gene clusters. BC KA P-value: Bonferroni-corrected Karlin-Altschul P value. Only lengths > 95 nt are shown. [file 1471-2148-7-69-S6.pdf]

Additional file 6. Gene conversion events in vertebrate multiple-variable-exon gene clusters

Species	Number of *Ugt1* sequences	Number of gene conversion events	Sequence name	Begin	End	Length	BC KA P-value
human	9	1	Ugt1a8;Ugt1a9	519	656	138	0.13491
chimpanzee	9	0					
rhesus monkey	10	2	Ugt1a7;Ugt1a8	130	256	127	0.10182
			Ugt1a8;Ugt1a9	515	621	107	0.11193
baboon	10	0					
dog	10	11	Ugt1a8;Ugt1a9	1	236	236	0.00001
			Ugt1a7;Ugt1a8	22	494	473	0.00003
			Ugt1a11;Ugt1a8	1	265	265	0.00006
			Ugt1a10;Ugt1a7	22	282	261	0.00019
			Ugt1a7;Ugt1a9	22	236	215	0.00103
			Ugt1a11;Ugt1a7	22	265	244	0.00133
			Ugt1a10;Ugt1a11	1	265	265	0.00165
			Ugt1a10;Ugt1a9	1	236	236	0.00278
			Ugt1a10;Ugt1a8	350	677	328	0.05561
			Ugt1a11;Ugt1a8	350	488	139	0.14857
			Ugt1a10;Ugt1a7	350	494	145	0.19117
mouse	9	0					
rat	8	1	Ugt1a4;Ugt1a6	54	245	192	< 0.00001
chicken	13	3	Ugt1a11;Ugt1a12	53	276	224	< 0.00001
			Ugt1a1;Ugt1a3	1	110	110	0.05196
			Ugt1a10;Ugt1a14	53	257	205	0.07249
zebrafish1a	7	0					
zebrafish1b	6	0					

Species	Number of *Gcnt2* sequences	Number of gene conversion events	Sequence name	Begin	End	Length	BC KA P-value
human	3	0					
chimpanzee	3	0					
rhesus monkey	3	0					
dog	3	0					
mouse	3	0					
rat	3	1	Gcnt2a;Gcnt2c	454	595	142	< 0.00001
opossum	2	0					
chicken	2	0					
frog	2	0					
zebrafish	1	0					

Species	Number of *Ugt2* sequences	Number of gene conversion events	Sequence name	Begin	End	Length	BC KA P-value
human	10	0					
mouse	10	0					
rat	11	2	Ugt2b34;Ugt2b39	879	1127	249	0.00006
			Ugt2b34;Ugt2b39	396	564	169	0.0047
zebrafish	4	1	Ugt2a3;Ugt2a4	14	117	104	< 0.00001

BC KA P-value: Bonferroni-corrected Karlin-Altschul P value.

Only lengths > 95 nt are shown.
