# Supplementary material for: Adaptive evolution of multiple-variable exons and structural diversity of drug-metabolizing enzymes
Source: BMC Evol Biol. 2007 May 2;7:69. doi: 10.1186/1471-2148-7-69 (PMC1885805; doi:10.1186/1471-2148-7-69)
Supplement: Additional file 7 — Alignment of vertebrate GCNT2 variable polypeptides (A, B, and C) with conserved residues highlighted. The predicted transmembrane segment is marked by a line below. The six identical cysteine residues are marked by asterisks below. Identical conserved residues are shown in black box shade, similar conserved residues in grew shade, and nonidentical residues are left with a white background. Abbreviations for species: GG, Gallus gallus; XT, Xenopus tropicalis; MMs, Mus musculus; RN, Rattus norvegicus; PT, Pan troglodytes; HS, Homo sapiens; MMa, Macaca mulatta; CF, Canis familiaris; MD, Monodelphis domestica; and DR, Danio rerio. [file 1471-2148-7-69-S7.pdf]

```
GGA       1 ~~~~MSVLRYCFFAVLILSISLPFVFYAINIHAQ....KSLRRLKFSVSSTLL..E.DCK
GGB       1 ~~~~~~MRRRRVAVTLTVAVVLLGAAMLLRRHG.....GSGAGLGGSAESMAL..ERACG
XTA       1 ~~~~MIYPKQLFFIFLALGMVILLNSILSQ........SQIKKALVFSQQISL.LEEACQ
XTB       1 ~~~~MPSWVRFIFPIPVLSVTVLLLYYNNSLLKL....AGLHRYNTTEEKLQ..FAETCE
MMsC      1 ~~~~MSL.RGKVFAVSALSVVIFVVFYHSQLSLP....NLYQQLNSSSER..T.SVTICD
RNC       1 ~~~~MNL.CMKFFAFTALSVVIFVVFYHSQLSLP....NLYQRFNSSSER..T.SVIACD
PTC       1 ~~~~MNFWRYCFFAFTLLSVVIFVRFYSSQLSPP....KSYEKLNSSSERY.F.RKTACN
HSC       1 ~~~~MNFWRYCFFAFTLLSVVIFVRFYSSQLSPP....KSYEKLNSSSERY.F.RKTACN
MMaC      1 ~~~~MNFWRYCFFAFILLSVVIFVRFYSSQLSPS....KSYEKLNSSSERY.F.RKTACN
CFC       1 ~~~~MNFWRCCLFALALLSALILLMVYHSQLHLP....RSLGKPSGPGPRH.V.RLRACE
MDB       1 ~~~~MNFGRYYRFVIPLLSLVLFFVLYNHHIYDS....KSYHRFNVSNERY.S.LYESCN
MMsA      1 ~~~~MGSWKYSLFSLSLIAALMLMFMYDRKLWKN....YHFPRA.VSNISV.L.A.EVCL
RNA       1 ~~~~MGSWKYSLFSLALIAALTFMFISNNKLWEN....NHFPRA.IPNSSV.L.A.EVCF
PTA       1 ~~~MMGSWKHCLFSVSLISALIFVFVYNTELWEN....KRFLRAALSNASL.L.A.EACH
HSA       1 ~~~MMGSWKHCLFSASLISALIFVFVYNTELWEN....KRFLRAALSNASL.L.A.EACH
MMaA      1 ~~~MMGSWKHCLFSVSLISALIFVFVYNTELWEN....KRFLRAALSNASL.L.A.EACH
CFA       1 ~~~MMVSWKHYLFTVSLITALVFVFVYNHKLWEE....KHFLRASLSNASV.L.T.EVCQ
PTB       1 ~~~~MPLSMRYLFIISVSSVIIFIVFSVFNFGGD....PSFQRLNISDPLM.L.T.QVCT
HSB       1 ~~~~MPLSMRYLFIISVSSVIIFIVFSVFNFGGD....PSFQRLNISDPLR.L.T.QVCT
MMaB      1 ~~~~MPLSMRYLFVISVSSVIIFIVFSVFNFGGD....QSFQRLNISDPLM.L.T.QVCT
MMsB      1 ~~~~MPPSVRYFFIVSVTTVIVFIVLYVLSFGGD....QSYQKLNISDSVM.L.A.QVCS
RNB       1 ~~~~MPPSMRHFFIVSVTTVIVFIVLYVLSFGGD....QSYQKLNISDSAM.L.A.QVCT
CFB       1 ~~~~MPSSTRYLFIASVSCVVVFIVCYMLGPGGE....QSFQKLNHSEALM.L.T.QVCT
MDA       1 ~~~~MPSLMHYLFVVPIFTVVFFILVYMFNIYSD....QSLPRLNITNTLV.L.A.KVCN
DR        1 MVQLENTKCSFLFCLGICTLICSAIYLKAKVASDPLPINDLTTLSPTPPPLPCPPSNTCD
Transmembrane segment  ________________                               *

GGA      50 ALIEDKVSFLKE....NALKTSFG.KFNCTEYITQNHYITRVLSAEEAAFPLAYIITMHK
GGB      48 MLLAGQAPTLRD....GGLRPAPR.DSSCSKYVSHSRYITRVLSAEEAAFPLAYIITMHK
XTA      48 ALAKTKKSFFWN....HLPKTYSH.SSNCKDFILKNNYITLPLSEEEAAFPLAYILTVHK
XTB      51 SFLNGQKSFAWE....STLLTAFE.KSSCPEYVRQNHFIMSPLSQEEAAFPLAYILTVHK
MMsC     49 YGLQNHTFFTTG....DTSPHPLE.RLSCPQYRIQSHYITSPLSEEEAAFPLAYIMVIHK
RNC      49 YGLQNHSVFVPG....ITSPHPLE.KLSCSQYQILSHYITSPLSEEEADFPLAYVMVIYK
PTC      51 HALEKTPVFLWE....NILPSPLR.SVPCKDYLTQNHYITSPLSEEEAAFPLAYVMVIHK
HSC      51 HALEKMPVFLWE....NILPSPLR.SVPCKDYLTQNHYITSPLSEEEAAFPLAYVMVIHK
MMaC     51 HALEKKPVFLWE....NILPSPLR.SVPCKDYLTQNHYITSPLSEEEVAFPLAYVTVIHK
CFC      51 VALEGKAAFLWA....NTLPSPVG.SVPCKDYLLQSHYITSPLSEEEVAFPLAYVMVIHK
MDB      51 AVVEGRPVFEWE....KVLVPSFG.RVSCEQYLLQSHYITAPLSKEEAQFPLAYVMVVHK
MMsA     49 QMFSGESFYTAD....SARKTTLE.NFTCPEYKIQNHYITETLSEEEARFPLAFTLTIHK
RNA      49 QMFSEESFYAVD....STRKTTLE.KFTCSEYRVQNHYITEALSEEEARFPLAFTLTIHK
PTA      51 QIFEGKVFYPTE....NALKTTLD.EATCYEYMVQSHYVTETLSEEEAGFPLAYTVTIHK
HSA      51 QIFEGKVFYPTE....NALKTTLD.EATCYEYMVRSHYVTETLSEEEAGFPLAYTVTIHK
MMaA     51 QIFEGKVFYPTE....NALKTTLD.EATCYEYMVRSHYVTETLSEEEAGFPLAYTVTIHK
CFA      51 QIFKGRVFYPTG....NALKTTLS.DSTCYEYMAQSHYITQTLSEEEAGFPLAYTVTIHK
PTB      50 SFINGKTRFLWK....NKLMIH.E.KSSCKEYLTQSHYITAPLSKEEADFPLAYIMVIHH
HSB      50 SFINGKTRFLWK....NKLMIH.E.KSSCKEYLTQSHYITAPLSKEEADFPLAYIMVIHH
MMaB     50 SFINGKTRFLWK....NKLMIH.E.KSSCKEYLTHSHYITAPLSKEEDDFPLAYIMVIHH
MMsB     50 SFIDGKSRFLWR....NKLMIH.E.KPSCTEYVTQSHYITAPLSQEEVDFPLAYVMVIHH
RNB      50 SFTNGKNSFLWR....NKLMIH.E.KPSCTEYVTQSHYITAPLSQEEVEFPLAYVMVIHH
CFB      50 SFIKGKAPFPWR....NKLMIH.Q.RTSCRDYLTRSHYITAPLSKEEADFALAYTMVIHH
MDA      50 SFIKGKTAFPLE....NTLTVS.G.K.SCKEYLLQSHYITTTLSKEEAEFPLAYAMVIHK
DR       61 ILPPATPGFKWQRKDCEKISYHIQPDTNCDLLLSQLHFITAPLSKEEEDYPLAFIITIHK
                                        *

GGA     105 EFETFERLFRAVYMPQNVYCIHVDGKAPAALKQAVRRLVDCFPNAFLASRTERVVYGGVS
GGB     103 EFETFERLFRAVYMPQNVYCIHVDGKAPAALKQAVRRLVDCFPNAFLASRTERVVYGGVS
XTA     103 EFETFERLFRAIYMPQNIYCVHVDEKASADFMQAVDSLVQCFPNTFLASKMEPVVYGGIS
XTB     106 EFETFERLFRAIYMPQNIYCVHVDEKASADFMQAVDSLVQCFPNTFLASKMEPVVYGGIS
MMsC    104 DFDTFERLFRAIYMPQNVYCVHVDSKATDTFKEAVRQLLSCFPNAFLASKVEQVVYGGFS
RNC     104 DFDTFERLFRAIYMPQNVYCVHVDSKAAETFKEAVRHLLSCFPNAFLASRMERVVYGGFS
PTC     106 DFDTFERLFRAIYMPQNVYCVHVDEKAPAEYKESVRQLLSCFQNAFIASKTESVVYAGIS
HSC     106 DFDTFERLFRAIYMPQNVYCVHVDEKAPAEYKESVRQLLSCFQNAFIASKTESVVYAGIS
MMaC    106 DFDTFERLFRAIYMPQNAYCVHVDEKAPAEFKESVRQLLSCFQNAFIASKTESVVYAGIS
CFC     106 NFETFERLFRAVYMPQNVYCVHVDEKAAAKFKESVRQLLSCFPNAFLASRMEPVVYGGIS
MDB     106 DFETFERLFRAVYMPQNVYCIHMDEKATTEFKDAVEWLVSCFSNVFLASKMEPVVYGGIS
MMsA    104 DYDTFERLFRAIYMPQNVYCVHVDSKATDTFKEEVRQLLSCFPNAFLASRMEPVVYGGFS
RNA     104 DYDTFERLFRAIYMPQNVYCVHVDSKAAETFKEAVRQLLSCFPNAFLASRMERVVYGGFS
PTA     106 DFGTFERLFRAIYMPQNVYCVHLDQKATDAFKGAVKQLLSCFPNAFLASKKESVVYGGIS
HSA     106 DFGTFERLFRAIYMPQNVYCVHLDQKATDAFKGAVKQLLSCFPNAFLASKKESVVYGGIS
MMaA    106 DFGTFERLFRAIYMPQNVYCVHLDQKATDAFKGAVKQLLGCFPNAFLASKKESVVYGGIS
CFA     106 DFDTFERLFRAIYMPQNVYCVHVDEKATDTFKNAVKQLLSCFPNAFLASKMEPVVYGGIS
PTB     104 HFDTFARLFRAIYMPQNIYCVHVDEKATTEFKDAVEQLLSCFPNAFLASKMEPVVYGGIS
HSB     104 HFDTFARLFRAIYMPQNIYCVHVDEKATTEFKDAVEQLLSCFPNAFLASKMEPVVYGGIS
MMaB    104 HFDTFARLFRAIYMPQNIYCIHVDEKATTEFKDAVEQLLSCFPNAFLASKMEPVVYGGIS
MMsB    104 NFDTFARLFRAIFMPQNIYCVHVDEKATAEFKGAVEQLVSCFPNAFLASKMEPVVYGGIS
RNB     104 NFDTFARLFRAIFMPQNVYCVHVDEKATAEFKGAVEQLVNCFPNAFLASKTEPVVYGGIS
CFB     104 HFETFARLFRAIYMPQNVYCVHVDEKATAEFKDAVEQLLSCFPNAFLASRMEPVVYGGIS
MDA     103 DFGTFERLLRAVYMPQNVYCVHVDEKATAEFKDAVGRLVSCFPNAFLASKMEPVVYGGIS
DR      121 ELATFVRLLRAIYAPQNVYCIHIDQKASEKYKSSVRNLSRCFPNVFLSSVNVKVTYAGFS
                               *                    *
```

```
GGA     165 RLRADLHCMRDLLASAVPWHYLLNACGQDFPLKTNWEIIQHLKA..YRGKNITPGVLPPA
GGB     163 RLRADLHCMRDLLASAVPWHYLLNACGQDFPLKTNWEIIQRLKA..YRGKNITPGVLPPA
XTA     163 RLQADLNCMKDLLASDVQWKYVINLCGQDFPLKTNREIIHHIKS..FKGKNITPGVLPPA
XTB     166 RLQADLNCMKDLLASDVQWKYVINLCGQDFPLKTNKEIIHHIKS..FKGKNITPGVLPPA
MMsC    164 RLQADLNCMKDLVASKVPWKYVLNTCGQDFPLKTNKEIINHLKR..FKGKNITPGVLPPA
RNC     164 RLQADLNCMRDLVASKVPWKYVINTCGQDFPLKTNKEIVQYLKG..FKGKNLTPGVLPPE
PTC     166 RLQADLNCLKDLVVSEVPWKYVINTCGQDFPLKTNREIVQHLKG..FKGKNITPGVLPPD
HSC     166 RLQADLNCLKDLVASEVPWKYVINTCGQDFPLKTNREIVQHLKG..FKGKNITPGVLPPD
MMaC    166 RLQADLNCLKDLVASEVPWKYVINTCGQDFPLKTNREIVQHLKG..FKGKNITPGVLPPD
CFC     166 RLQADLNCLKDLAASQVPWKYAINTCGQDFPLKTNKEIVRHLKG..FKGKNITPGVLPPS
MDB     166 RLQADLNCMKDLVASQIQWKYLINTCGQDFPLKTNKEIIQHLKG..FKGKNITPGVLPPA
MMsA    164 RLQADLNCMKDLVASKIPWKYVLNTCGQDFPLKTNKEIVQYLKR..FIGKNLTPGVLPPA
RNA     164 RLQADLNCMRDLVASKVPWKYVINTCGQDFPLKTNREIIQYLKG..FLGKNLTPGVLPPA
PTA     166 RLQADLNCLEDLVASEVPWKYVINTCGQDFPLKTNREIVQYLKG..FKGKNITPGVLPPD
HSA     166 RLQADLNCLEDLVASEVPWKYVINTCGQDFPLKTNREIVQYLKG..FKGKNITPGVLPPD
MMaA    166 RLQADLNCLEELVASEVPWKYVINTCGQDFPLKTNREIVQYLKR..FKGRNITPGVLPPD
CFA     166 RLQADLNCLKDLGASEVPWKYAINTCGQDFPLKTNKEIVRYLKG..FKGKNITPGVLPPA
PTB     164 RLQADLNCIRDLSAFEVSWKYVINTCGQDFPLKTNKEIVQYLKG..FKGKNITPGVLPPA
HSB     164 RLQADLNCIRDLSAFEVSWKYVINTCGQDFPLKTNKEIVQYLKG..FKGKNITPGVLPPA
MMaB    164 RLQADLNCIKDLSALEVSWKYVINTCGQDFPLKTNKEIVQYLKG..FKGKNITPGVLPPA
MMsB    164 RLQADLNCIKDLSTSEVPWKYAINTCGQDFPLKTNKEIVQYLKG..LKGKNLTPGVLPPA
RNB     164 RLQADLNCIRDLSTSEVPWKYAINTCGQDFPLKTNKEIVQYLKG..LKGKNLTPGVLPPA
CFB     164 RLQADLNCLKDLAASQVPWKYAINTCGQDFPLKTNKEIVRYLKG..YRGKNITPGVLPPS
MDA     163 RLQADLNCMKDLVASQIQWKYLINTCGQDFPLRTNKEIIQHLKG..FKGKNITPGVLPPA
DR      181 RLQADINCMKDLVESPIQWKKVINLCGQDYPIQTNLELVRYMQTPEWKDRNMTPGIKQPP
                   *                 *

GGA     223 HVTARTKYVHREQLYSLFSFMLPMFVHKAPPPHNLTLYFGSAYIAVTRPFAEFVLQDPRA
GGB     221 HVTMRTRFMHLEQGGSNVSELVTPQVHKAPPPHNLTLYFGSAYIAVTRPFAEFVLQDPRA
XTA     221 HAIPRTKYVHRE..DIVNSRVVRTRVLKPPPPHNITIYFGSAYVALTREFTRFILEDQRA
XTB     224 HAIPRTKYVHRE..DIVNSRVVRTRVLKPPPPHNITIYFGSAYVALTREFTRFILEDQRA
MMsC    222 YIVVRTKYVHQERKGKDGYFMHKTNILKTPPPHQLIIYFGTAYVALTRDFVNFILNDERA
RNC     222 HVITRTKYVYKERKGRDGYFMQNTNTLKTPPPHKLVIYFGTAYVALTRDFVNFILNDKRA
PTC     224 HAIKRTKYVHQEHTDKGGFFVKNTNILKTSPPHQLTIYFGTAYVALTREFVDFVLRDQRA
HSC     224 HAIKRTKYVHQEHTDKGGFFVKNTNILKTSPPHQLTIYFGTAYVALTRDFVDFVLRDQRA
MMaC    224 HAIKRTKYVHQEHTDKGGSFVKTTNILKTSPPHQLTIYFGTAYVALTREFVNFVLHDKKA
CFC     224 HAVKRTKFVHREHIGKDGSFVKNTNVLKTSPPHQMTIYFGTAYVALTREFVDFIFHDKRA
MDB     224 HAIERTKYVFREYMSQKASYMEKTKILKSSPPHKLVIYFGSAYVALTKEFVNFVFQDHRA
MMsA    222 HAVGRTKYVHQELLDHKNPYVHNTARLKAPPPHNLTIYFGTAYVALTREFANFVLKDQRS
RNA     222 HAVGRTKYVHRELLDLKNPYVHNTARLKTPPPHNLTIYFGTAYVALTREFANFVLTDQRS
PTA     224 HAVGRTKYVHQELLDHKNSYVIKTTKLKTPPPHDMVIYFGTAYVALTRDFANFVLQDQLA
HSA     224 HAVGRTKYVHQELLNHKNSYVIKTTKLKTPPPHDMVIYFGTAYVALTRDFANFVLQDQLA
MMaA    224 HAVGRTKYVHQELLDHKNSYVIKTTKLKTPPPHDMVIYFGTAYVALTRDFANFVLQDQLA
CFA     224 HAIGRTKYVHRELLSKKNSYMLKTTQLKTPPPHNMTIYFGTAYVALTREFANFVLQDQHA
PTB     222 HAIGRTKYVHQEHLGKELSYVIRTTAMKPPPPHNLTIYFGSAYVALSREFANFVLHDPRA
HSB     222 HAIGRTKYVHQEHLGKELSYVIRTTALKPPPPHNLTIYFGSAYVALSREFANFVLHDPRA
MMaB    222 HAIGRTKYVHQEHLGKELSYVIRTTALKPPPPHNLTIYFGSAYVALSREFANFVLHDPRA
MMsB    222 HAIGRTRYVHREHLSKELSYVIRTTALKPPPPHNLTIYFGSAYVALSREFANFVLRDPRA
RNB     222 HAIGRTRYVHREHLSKEFSYVIRTAALKPPPPHNLTIYFGSAYVALSREFANFVLHDPRA
CFB     222 HAIGRTKYVHREHLGKELSYVIRTTALKPPPPHNLTIYFGSAYVALSREFTDFVLHDPRA
MDA     221 HAIERTKYIHREHLGLEASYVINTQALKSPPPHNLTIYFGSAYVALTREFINFVLQDQRA
DR      241 SMRYRTAF...QYVEVKNTHVAQTGRKKGPPPHNLKIYFGTAYYSLTRPFVEYVLDNPVA

GGA     283 IDLLAWSEDTYSPDEHFWVTLNRIP
GGB     281 IDLLAWSEDTYSPDEHFWVTLNRIP
XTA     279 TNLLLWSKDTYSPDEHYWVTLNRIA
XTB     282 TNLLLWSKDTYSPDEHYWVTLNRIA
MMsC    282 IALLEWSKDTYSPDEHFWVTLNRIP
RNC     282 IDLLEWSKDTYSPDEHFWVTLNRIP
PTC     284 IDLLRWSKDTYSPDEHFWVTLNRVS
HSC     284 IDLLQWSKDTYSPDEHFWVTLNRVS
MMaC    284 IDLLQWSKDTYSPDEHFWVTLNRVS
CFC     284 IDLLHWSKDTYSPDEHFWVTLNRIP
MDB     284 IDLLQWSRDTYSPDEHFWVTLNRLT
MMsA    282 VDLISWSKDTYSPDEHFWVTLNRIP
RNA     282 LDLISWSKDTYSPDEHFWVTLNRIP
PTA     284 LDLLSWSKDTYSPDEHFWVTLNRIP
HSA     284 LDLLSWSKDTYSPDEHFWVTLNRIP
MMaA    284 LDLLSWSKDTYSPDEHFWVTLNRIP
CFA     284 LDLLSWSKDTYSPDEHFWVTLNRIP
PTB     282 VDLLQWSKDTFSPDEHFWVTLNRIP
HSB     282 VDLLQWSKDTFSPDEHFWVTLNRIP
MMaB    282 VALLQWSKDTFSPDEHFWVTLNRIP
MMsB    282 VDLLHWSKDTFSPDEHFWVTLNRIP
RNB     282 VDLLHWSKDTFSPDEHFWVTLNRIP
CFB     282 VDLLQWSKDTFSPDEHFWVTLNRIP
MDA     281 IDFLQWSKDTYSPDEHFWVTLNRIP
DR      298 KDLLSWSKDSYSPDEHYWVTLNHIK
```
