# Supplementary figures and images for: Adaptive evolution of multiple-variable exons and structural diversity of drug-metabolizing enzymes
Source: BMC Evol Biol. 2007 May 2;7:69. doi: 10.1186/1471-2148-7-69 (PMC1885805; doi:10.1186/1471-2148-7-69)

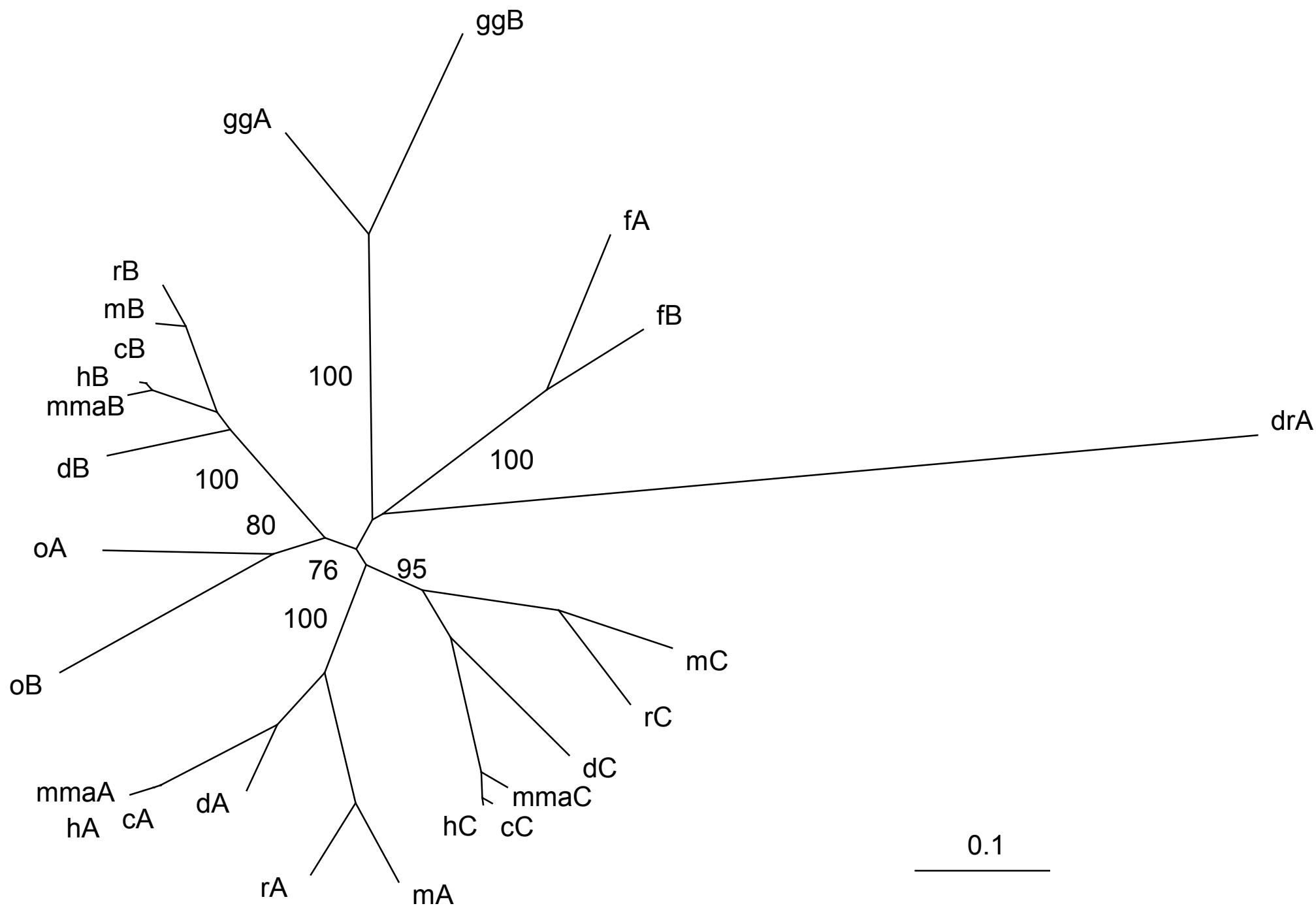

Supplement: Additional file 8 — Phylogenetic tree of the human (h), chimpanzee (c), rhesus monkey (Macaca mulatta [mma]), dog (d), mouse (m), rat (r), opossum (o), chicken (Gallus gallus [gg]), frog (f), and zebrafish (Danio rerio [dr]) Gcnt2 clusters. The tree branches are labeled with the percentage support for that partition based on 1,000 bootstrap replicates. Only bootstrap values of >50% on major branches are shown. The scale bar equals a distance of 0.1. [file 1471-2148-7-69-S8.pdf]

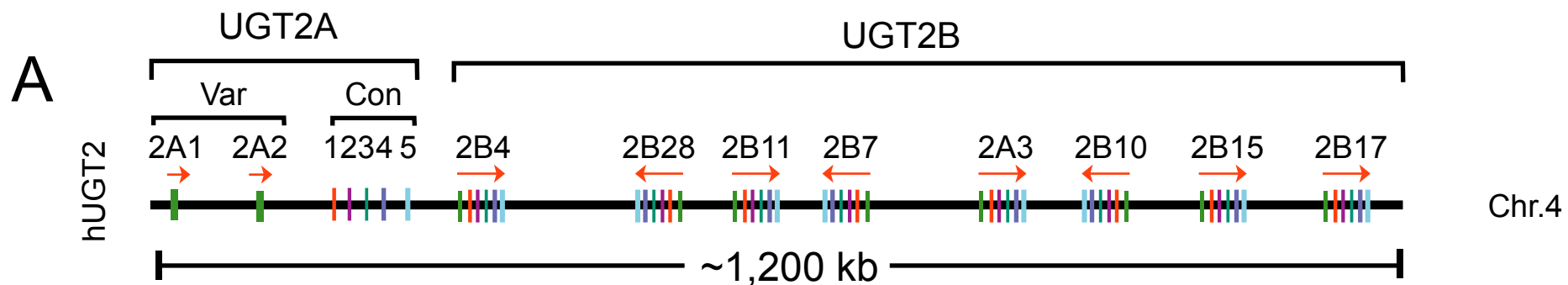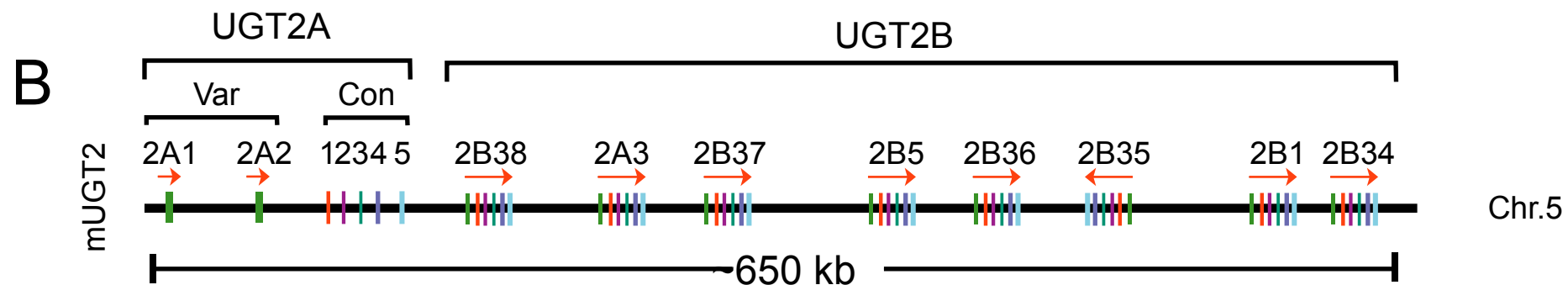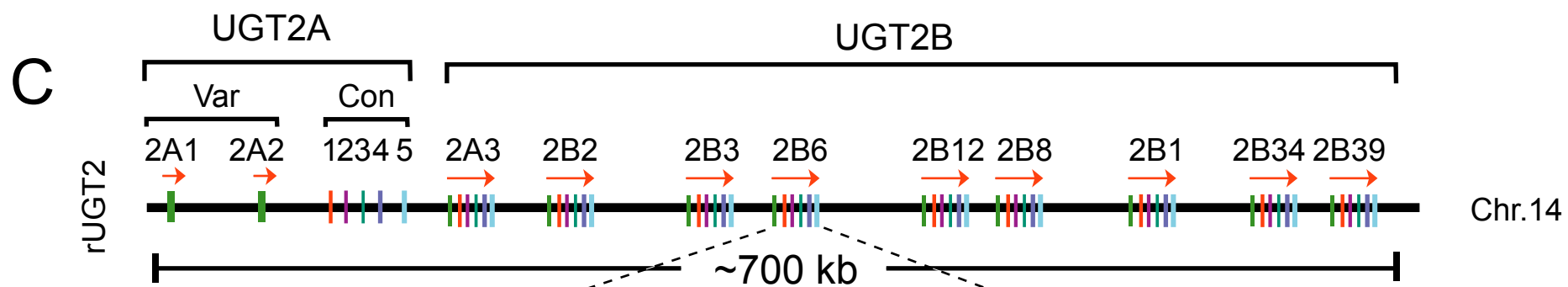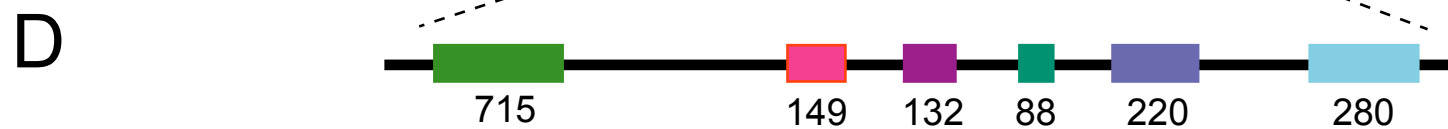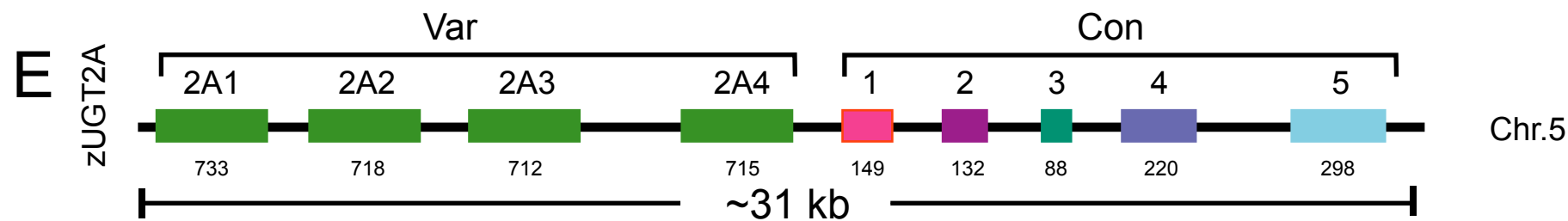

Supplement: Additional file 10 — Comparison of the human (h) (A), mouse (m) (B), and rat (r) (C) Ugt2 clusters with transcription direction marked by an arrow above each functional gene (pseudogenes are not shown), and their conserved genomic organization (D) (representing both Ugt2a and Ugt2b) with exon length indicated. (E) The organization of the zebrafish (z) Ugt2a gene cluster with exon length indicated. The exons of the Ugt2a are represented by vertical colored bars: (green) variable exon; (magenta) first constant exon; (purple) second constant exon; (teal) third constant exon; (blue) fourth constant exon; and (light blue) fifth constant exon. The corresponding six exons in individual Ugt2b genes are similarly colored. The approximate length of each cluster is shown below the corresponding panels. The chromosomal location is indicated on the right for each cluster. Var, variable; Con, constant; and kb, kilobase pairs. [file 1471-2148-7-69-S10.pdf]

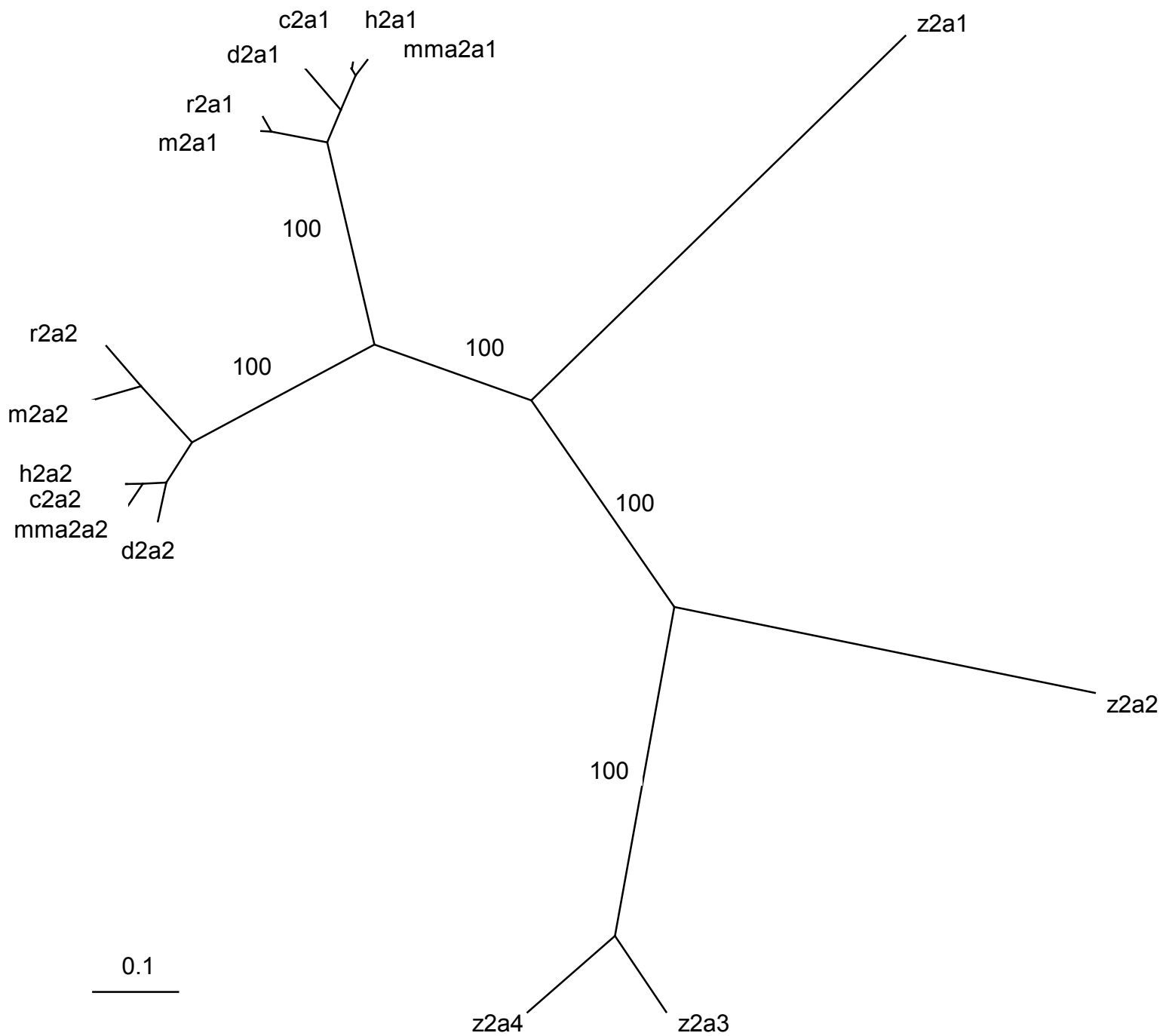

Supplement: Additional file 11 — Phylogenetic tree of the human (h), chimpanzee (c), rhesus macaque (Macaca mulatta [mma]), dog (d), mouse (m), rat (r), and zebrafish (z) Ugt2a clusters. The major branches of the tree are labeled with the percentage support for that partition based on 1,000 bootstrap replicates. Only bootstrap values of >50% on major branches are shown. The scale bar equals a distance of 0.1. [file 1471-2148-7-69-S11.pdf]

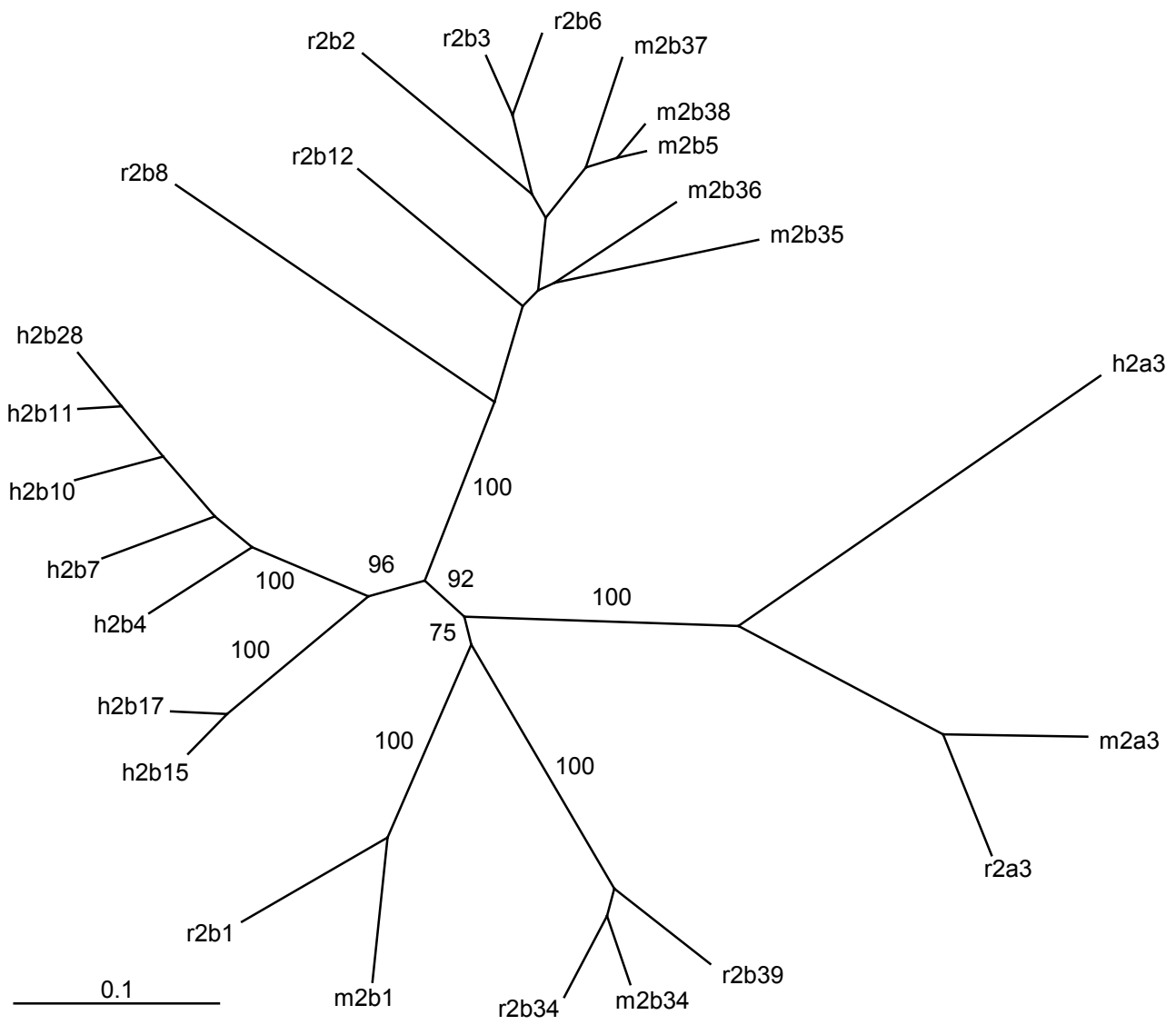

Supplement: Additional file 14 — Phylogenetic tree of the human (h), mouse (m), and rat (r) Ugt2b clusters. The major tree branches are labeled with the percentage support for that partition based on 1,000 bootstrap replicates. The scale bar equals a distance of 0.1. [file 1471-2148-7-69-S14.pdf]
