# Supplementary material for: Adaptive evolution of multiple-variable exons and structural diversity of drug-metabolizing enzymes
Source: BMC Evol Biol. 2007 May 2;7:69. doi: 10.1186/1471-2148-7-69 (PMC1885805; doi:10.1186/1471-2148-7-69)
Supplement: Additional file 9 — Alignment of the vertebrate GCNT2 constant polypeptides with conserved residues highlighted. The three identical cysteine residues are marked by asterisks below. Identical conserved residues are shown in black box shade, similar conserved residues in grew shade, and nonidentical residues are left with a white background. Abbreviations for species: HS, Homo sapiens; PT, Pan troglodytes; MMa, Macaca mulatta; CF, Canis familiaris; MMs, Mus musculus; RN, Rattus norvegicus; MD, Monodelphis domestica; GG, Gallus gallus; XT, Xenopus tropicalis; and DR, Danio rerio. [file 1471-2148-7-69-S9.pdf]

```
HS      1 VPGSM.PNASWTGNLRAIKWSDME.DRHGGCHGHYVHGICIYGNGDLKWLVNSPSLFANK
PT      1 VPGSM.PNASWTGNLRAIKWSDME.DRHGGCHGHYVHGICIYGNGDLKWLVNSPSLFANK
MMa     1 VPGSM.PNASWTGNLRAIKWSDME.DSHGGCHGHYVHGICIYGNGDLKWLVNSPSLFANK
CF      1 VPGSM.PNASWTGNLRAIKWIDME.DKHGGCHGRYVRGICIYGNGDLKWLIDSPSLFANK
MMs     1 VPGSMPPNASWTGNLRAVKWMDME.AKHGGCHGHYVHGICIYGNGDLQWLINSQSLFANK
RN      1 VPGAM.PNASWTGNLRAVKWKDME.SQNGACHGHYVHDICIYGNGDLQWLINSQSLFANK
MD      1 VPGSM.PNASWEGNLRAIKWHDME.KDHGGCHGHYIHGICIFGNGDLKWLYDSPNMFANK
GG      1 VPGSM.PNASWEGGLKAVKWIDME.DIHGGCHGHYVRGICVYGTGDLKWLFNSTCMFANK
XT      1 VPGS.APDASWEGQLRAVKWKDMK.DQE.KCHGHYVRDICIYGTGDLQWLMNSRSIFANK
DR      1 APGSNV.EGEWEGNVRAVKWSDQQGTAHQGCKGQYIRGICVYGIGDLPWLIEKESMFANK
                                        *        *

HS     59 FELNTYPLTVECLELRHRERTLNQSETAIQPSWYF~~~~~~~~~~~~~~~~~~~
PT     59 FELNTYPLTVECLELRHRERTLNQSETAIQPSWYF~~~~~~~~~~~~~~~~~~~
MMa    59 FELNTYPLTVECLELRHRERTLNQSETAIQPSWYF~~~~~~~~~~~~~~~~~~~
CF     59 FELTTYPLTVECLELRLRERTLNQSETVIQPSWYF~~~~~~~~~~~~~~~~~~~
MMs    60 FELNTYPLTVECLELRLRERTLNQSEIAIQPSWYF~~~~~~~~~~~~~~~~~~~
RN     59 FEVNTYPLTVECLELRLRERTLNQSDIALQPSWHF~~~~~~~~~~~~~~~~~~~
MD     59 FELKTYPLTLECLELKLRERALNQSETPIRSSWYL~~~~~~~~~~~~~~~~~~~
GG     59 FELRTYPLTVECLELRHRKRTLAQSEVQVEPNWYF~~~~~~~~~~~~~~~~~~~
XT     58 FEAKSYPPTVECLELKVRERTLNQSEVTVPPEWYL~~~~~~~~~~~~~~~~~~~
DR     60 FEMASFPEALDCMELWHRHKVLQQATVPIQPSWHLTTEVEVVNRTLILTPGDCV
                     *
```
