# Supplementary material for: Adaptive evolution of multiple-variable exons and structural diversity of drug-metabolizing enzymes
Source: BMC Evol Biol. 2007 May 2;7:69. doi: 10.1186/1471-2148-7-69 (PMC1885805; doi:10.1186/1471-2148-7-69)
Supplement: Additional file 12 — Alignment of the vertebrate UGT2A variable polypeptides with conserved residues highlighted. The predicted signal peptides are indicated by a line below. Identical conserved residues are shown in black box shade, similar conserved residues in grew shade, and nonidentical residues are left with a white background. Abbreviations for species: DR, Danio rerio; MMs, Mus musculus; RN, Rattus norvegicus; PT, Pan troglodytes; HS, Homo sapiens; MMa, Macaca mulatta; and CF, Canis familiaris. [file 1471-2148-7-69-S12.pdf]

```
DR2a3        1 ~~~~~~~MTSGALLVCLLLCGVDVGWSGRVLVMPGEYSHWHNMRAIIEALVDRNHSVTVL
DR2a4        1 ~~~~~~~MTSGTLLVCLLLCGVDVGWSGRVLVMPGEYSHWHNMRAIVEALVDRNHSVTVL
DR2a2        1 ~~~MMKCLLKPSPLVLIFI..FSFTHAGNVLVLPGEYSHWQNMRNVVDELLNRNHTVTVL
MMs2a1       1 ~~~~~~~MLKNILLCSLQISLLGMSLGGNVLIWPMEGSHWLNVKIIIDELLRKEHNVTVL
RN2a1        1 ~~~~~~~MLKNILLWSLQLSLLGMSLGGNVLIWPMEGSHWLNVKIIIDELLRKEHNVTVL
PT2a1        1 ~~~~~~MLN.NLLLFSLQISLIGTTLGGNVLIWPMEGSHWLNVKIIIDELIKKEHNVTVL
HS2a1        1 ~~~~~~MLN.NLLLFSLQISLIGTTLGGNVLIWPMEGSHWLNVKIIIDELIKKEHNVTVL
MMa2a1       1 ~~~~~~MLN.NLLLFSLQISLIGTTLGGNVLIWPMEGSHWLNVKIIIDELIKKEHNVTVL
CF2a1        1 ~~~~~~MLNKNILLLSLKISLLGITLGGNVLIWPMEGSHWLNIKIIIDELIKKEHNVTVL
PT2a2        1 ~~~~~~MPKKFVQMLVFNLTLTEVVLSGNVLIWPTDGSHWLNIKIILEELIQRNHNVTVL
HS2a2        1 ~~~~~~MPKKFVQMLVFNLTLTEVVLSGNVLIWPTDGSHWLNIKIILEELIQRNHNVTVL
MMa2a2       1 ~~~~~~MPKKFVQMLVFNLTLTEIALSGNVLIWPTDGSHWLNIKIILEELIQRNHNVTVL
CF2a2        1 ~~~~~~MPKKFVQLLVFNLTLTEIVLSGNVLIWPTDGSHWLNIKIILEELIQRNHNVTVL
MMs2a2       1 ~~~~~~MIKKVLQLLIFHLTLAEIVLSGNVVVWPTDGSHWLNIKILLEELVQRNHSVTVL
RN2a2        1 ~~~~~~MIKKVLQLLIFPLALTEIVLSGNVVIWPSDGSHWLNIKILLEELVQRNHSVTVL
DR2a1        1 MSPRGSTGVFLLLALLLNQNLLCVS.GGKILVWPAEFSHWLNIKVILDVLIERGHNITVV
Signal peptide ————————————————————————————

DR2a3       54 VGSSSPTVPHTQ.KARFDYHVFKVNMDKEEADAVWSDFIYLWMNE..TATKYETVSH.IF
DR2a4       54 VSSSSPTVPHTQ.KERFDYHVFKVNMDKEEAKVIWSDFTHLWMKE..TDSKLERGFL.IW
DR2a2       56 VCSASPTINFTQ.QERFQYLVFEVPLSAQELDGASEELIHIWTQH.PSPNRLQTALQ.II
MMs2a1      54 VASGALFITPSSISPSLTFEIYPVPFGKEKIESVIKDFVLTWLENRPSPSTIWTFYKEMA
RN2a1       54 VASGALFITP.SVSPSLTFEIYPVPFGKEKIESVIKDFVLTWLENRPSPSTIWTFYKEMA
PT2a1       54 VASGALFITPTS.NPSLTFEIYKVPFGKERIEGVIKDFVLTWLENRPSPSTIWRFYQEMA
HS2a1       54 VASGALFITPTS.NPSLTFEIYRVPFGKERIEGVIKDFVLTWLENRPSPSTIWRFYQEMA
MMa2a1      54 VASGALFITPTS.NSSLTFEIYKVPFGKERIEGVINDIVLTWLENRPSPSTIWRFYQKMA
CF2a1       55 VASGALFITPTF.NPSLTFEIYKVPFDKGRVEGIIKEFVLTWMENRPSPSTIWRFYQEMA
PT2a2       55 ASSATLFIN.SNPDSPVNFEVIPVSYKKSNIDSLIEHMIMLWIDHRPTPLTIWAFYKELG
HS2a2       55 ASSATLFIN.SNPDSPVNFEVIPVSYKKSNIDSLIEHMIMLWIDHRPTPLTIWAFYKELG
MMa2a2      55 ASSATLFIN.SNPDSPVNFEVIPVSYKKSNVDSLIEHMIMLWIDHRPTPLTIWAFYKELG
CF2a2       55 ASSTTLFIN.SNPDSSVNFEVIPVSFTNDNLDSLIEHMIMLWIDHRPTPLTLWSFYKELG
MMs2a2      55 APSETLFIN.SRLDAFINFEEIPVSYTKSKIDEIIEHMIALWLDHRPTPLTMWTFYKELG
RN2a2       55 AASETLFIN.SSPDAFISFEEIPVSYTRSKIDEMIEHMIALWLDHRPTPLTMWAFYKELG
DR2a1       60 THTATPSV.QTTPSAGYNAEILQVPYTKQEIVDNLERMLKYWTHDLPNDNIITASLK.IH

DR2a3      110 QVMSRFMTLTDEVCKGMF.NEDLLQMLRESHYNVLFSDPMMPCSDLMAQTLNIPLVLSLR
DR2a4      110 RVMSNFRRLAADVCRSLLQNDDLLQMLGESHYDVLFSDPMMPCSDLMAQTLNIPQVISLR
DR2a2      113 ELLGRVRVMHRAMCDSMLRNDALIGQLSALKFDVLFNDPMFMCADLLAEMLDLPLVLSVR
MMs2a1     114 KVIEEFHLVSRGICDGVLKNEKLMSKLQKEKFEVLLSDPVFPCGDIVALKLGIPFIYSLR
RN2a1      113 KVIEEFHLVSRGICDGVLKNEKLMTKLQRGKFEVLLSDPVFPCGDIVALKLGIPFIYSLR
PT2a1      113 KVIKDFHMVSQEICDGVLKNQQLMEKLKKSKFEVLVSDPVFPCGDIVALKLGIPFMYSLR
HS2a1      113 KVIKDFHMVSQEICDGVLKNQQLMAKLKKSKFEVLVSDPVFPCGDIVALKLGIPFMYSLR
MMa2a1     113 KAIKNFHIVSREICDGVLKNQQLMEKLKKSKFEVLVSDPVFPCGDIVALKLGIPFMYSLR
CF2a1      114 KVIKNFHMLSREICDGVLKNQKLMDKLKKSKFEVLISDPVFPCGDIVALKLGVPFMYSLR
PT2a2      114 KLLDTFFQINIQLCDGVLKNPKLMARLQKGGFDVLVADPVTICGDLVALKLGIPFMYTLR
HS2a2      114 KLLDTFFQINIQLCDGVLKNPKLMARLQKGGFDVLVADPVTICGDLVALKLGIPFMYTLR
MMa2a2     114 NLLDTFFQINMQICDGVLNNPKLMERLQKGGFEVLVADPVTICGDLIALKLGIPFVYTLR
CF2a2      114 KLLDTFFRINIQICDGVLSNPKLMARLQKRGFDVLVADPVTICGDLVALKLGIPFVYTLR
MMs2a2     114 NLLATFYTTNKQMCDGVLNNPTVMERLQKGGFDVLLADPVTMCGELVALKLGIPFVYTLR
RN2a2      114 KLFAAFYKINKQICDGVLKNATLMARLQKGGFDVLISDPVTMCGELVALKLGIPFVYTLR
DR2a1      118 EMITTATAQNKITCNALFSRAELLEKWKMEKFDVILADPLYICGEILAQKLDIPLIFSLR

DR2a3      169 ATFAYSFERMCGQMPAPPSYVPAASLRDYLTDRMSFMERVENMLLYFSHDVFFKLYMMFTFDRIYTEIR
DR2a4      170 LTFAYTFERMCGQMPAPPSYVPAVALTDHLTDRMSFMERVENMLLYFIHTTVFRLNTMLTVDRLYTEIR
DR2a2      173 ISPGFCLERMCGQMPAPPSFVPIT..QTVLTDRMCFMERVQNMIANIVFSVSFYIVAWISLDSYYTDVL
MMs2a1     174 FSPASTVEKHCGKVPFPPSYVPAIL..SELTDQMSFTDRVRNFISYRMQDYMFETLWK.QWDSYYTKAL
RN2a1      173 FSPASTVEKHCGKVPFPPSYVPAIL..SELTDQMSFADRVRNFISYRMQDYMFETLWK.QWDSYYSKAL
PT2a1      173 FSPASTVEKHCGKVPYPPSYVPAVL..SELTDQMSFTDRIRNFISYHLQDYMFETLWK.SWDSYYSKAL
HS2a1      173 FSPASTVEKHCGKVPYPPSYVPAVL..SELTDQMSFTDRIRNFISYHLQDYMFETLWK.SWDSYYSKAL
MMa2a1     173 FSPASTVEKHCGKVPYPPSYVPAVL..SELTDQMSFTDRIRNFISYHLQDYMFETLWK.SWDSYYSKAL
CF2a1      174 FSPASTVEKHCGKVPFPPSYVPAIL..SELTDQMSFTDRVRNFISYSLQDYMFDTLWK.SWDSYYSKAL
PT2a2      174 FSPASTVERHCGKIPAPVSYVPAAL..SELTDQMTFGERIKNTISYSLQDYIFQSYWG.EWNSYYSKIL
HS2a2      174 FSPASTVERHCGKIPAPVSYVPAAL..SELTDQMTFGERIKNTISYSLQDYIFQSYWG.EWNSYYSKIL
MMa2a2     174 FSPASTVERHCGKIPAPVSYVPAAL..SELTDQMTFGERIKNMISYSLQDYIFQSYWG.EWNSYYSKIL
CF2a2      174 FSPASTVERHCGKIPAPASYVPAAL..SELTDQMTFGERVKNTISYPLQDYIFQSYWG.EWNSYYSKVL
MMs2a2     174 FSPAFTVERHCGKIPAPISYVPAAL..SELTDQMSFGERVKNIISYSLQDYIFKTYWG.EWNSYYSKVL
RN2a2      174 FSPAFTVERHCGKIPTPVSYVPAAL..SELTDQMTFGERVKNTISYSLQDYIFKSYWG.EWNSYYSRVL
DR2a1      178 FTFGNTLERLCGQMPAPPSYVPAVA..SEKTDQMDFIDRLKNYLFYGMQDFLFYLVTKFKWDHYYSEVL
```
