# Supplementary material for: Adaptive evolution of multiple-variable exons and structural diversity of drug-metabolizing enzymes
Source: BMC Evol Biol. 2007 May 2;7:69. doi: 10.1186/1471-2148-7-69 (PMC1885805; doi:10.1186/1471-2148-7-69)
Supplement: Additional file 13 — Alignment of the vertebrate UGT2A constant polypeptides with conserved residues highlighted. The predicted 17-aa transmembrane segment across the ER is marked by a line below. Identical conserved residues are shown in black box shade, similar conserved residues in grew shade, and nonidentical residues are left with a white background. Abbreviations for species: MMs, Mus musculus; RN, Rattus norvegicus; CF, Canis familiaris; PT, Pan troglodytes; HS, Homo sapiens; MMa, Macaca mulatta; and DR, Danio rerio. [file 1471-2148-7-69-S13.pdf]

```
MMs     1 RPTTLCETMGKAEIWLMRTYWDFEFPRPYLPNFEFVGGLHCKPAKPLPKEMEEFVQTSGE
RN      1 RPTTLCETMGKAEIWLMRTYWDFEFPRPYLPNFEFVGGLHCKPAKPLPKEMEEFVQTSGE
CF      1 RPTTLCEIMGKAEIWLIRTYWDFEFPRPYLPNFEFVGGLHCKPAKPLPKEMEEFVQSSGE
PT      1 RPTTLCETMGKAEIWLIRTYWDFEFPRPYLPNFEFVGGLHCKPAKPLPKEMEEFIQSSGK
HS      1 RPTTLCETMGKAEIWLIRTYWDFEFPRPYLPNFEFVGGLHCKPAKPLPKEMEEFIQSSGK
MMa     1 RPTTLCETVGKAEIWLIRTYWDFEYPQPYQPNFEFVGGLHCKPAKPLPKEMENFVQSSGE
DR      1 KPTTMCETMGKADIWLIRTYWDFEYPRPFPPNFKFVGGLHCKPAKPLSKEMEEFVQSSGD

MMs    61 HGIVVFSLGSMVKNLTDEKANLIASALAQIPQKVLWRYKGKIPDTLGSNTRLFDWIPQND
RN     61 HGVVVFSLGSMVKNLTEEKANLIASALAQIPQKVLWRYKGKIPATLGSNTRLFDWIPQND
CF     61 DGVVVFSLGSMVKNLTDEKANLIASALAQIPQKVLWRYKGNKPATLGTNTRLYDWIPQND
PT     61 DGVVVFSLGSMVKNLTEEKANLIASALAQIPQKVLWRYKGKKPATLGNNTQLFDWIPQND
HS     61 NGVVVFSLGSMVKNLTEEKANLIASALAQIPQKVLWRYKGKKPATLGNNTQLFDWIPQND
MMa    61 DGIVVFSLGSLFQNVTEEKANIIASALAQIPQKVLWRYKGKKPSTLGTNTRLYDWIPQND
DR     61 HGVVVFSLGSMIKNLTSERANTIAAALGQIPQKVVWRYSGKTPETLAPNTKIYDWIPQND

MMs   121 LLGHPKTRAFITHGGTNGIYEAIYHGIPMVGVPMFADQPDNIAHMKAKGAAVEVNMNTMT
RN    121 LLGHPKTRAFITHGGTNGIYEAIYHGIPMVGVPMFADQPDNIAHMKAKGAAVEVNMNTMT
CF    121 LLGHPKTKAFITHGGTNGIYEAIYHGVPMVGVPMFADQPDNIAHMKAKGAAVEVNINTMT
PT    121 LLGHPKTKAFITHGGTNGIYEAIYHGVPMVGIPMFADQPDNIAHMKAKGAAVEVNLNTMT
HS    121 LLGHPKTKAFITHGGTNGIYEAIYHGVPMVGVPMFADQPDNIAHMKAKGAAVEVNLNTMT
MMa   121 LLGHPQTKAFITHGGMNGVYEAIYHGVPMVGVPIFGDQPDNIAHMKAKGAAVEINFKTMT
DR    121 LLGHPKTKAFITHGGTNGLYEAIYHGVPMVGLPLFADQPDNLLHMKTKGAAVVLDINTLE

MMs   181 SSDLLNALRTVINEPSYKENAMRLSRIHHDQPVKPLDRAVFWIEFVMRHKGAKHLRVAAH
RN    181 SADLLSAVRAVINEPFYKENAMRLSRIHHDQPVKPLDRAVFWIEFVMRHKGAKHLRVAAH
CF    181 SADLLHALRTVINEPSYKENATRLSRIHHDQPVKPLDRAVFWIEFVMRHKGAKHLRPASH
PT    181 SVDLLSALRTVINEPSYKENAMRLSRIHHDQPVKPLDRAVFWIEFVMRHKGAKHLRVAAH
HS    181 SVDLLSALRTVINEPSYKENAMRLSRIHHDQPVKPLDRAVFWIEFVMRHKGAKHLRVAAH
MMa   181 SEDLLRAVRTVITDSSYKENAMRLSRIHHDQSVKPLDRAVFWIEFVMRHKGAKHLRSAAH
DR    181 SKDLVDALKTVLNNPSYKESIMRLSRIHHDQPMKPLDQAVYWIEFVMRNKGAKHLRVQAH

MMs   241 DLSWFQYHSLDVIGFLLACVASAILLVAKCCLFIFQK.VGKTGKKKKRD~~~~~
RN    241 DLSWFQYHSLDVIGFLLACMASAILLVIKCCLFVFQK.IGKTGKKNKRD~~~~~
CF    241 DLTWFQYHSLDVIGFLLACVATAIFLVTKCCLFSCRK.FNKAGKKKKRE~~~~~
PT    241 NLTWFQYHSLDVIGFLLVCVTTAIFLVIQCCLFSCQK.FVKIGKKKKRE~~~~~
HS    241 DLTWFQYHSLDVIGFLLVCVTTAIFLVIQCCLFSCQK.FGKIGKKKKRE~~~~~
MMa   241 DLNWFQHHSIDVIAFLLACVATAIFLFTRCFLFSCQN.FNKTRKIEKRE~~~~~
DR    241 ELSWYQYHCLDVAAFLLSITALITFLWVKTCCFLFRRCVRKTHPERKTQKSKKE
Transmembrane segment ——————————————
```
