# Supplementary material for: Adaptive evolution of multiple-variable exons and structural diversity of drug-metabolizing enzymes
Source: BMC Evol Biol. 2007 May 2;7:69. doi: 10.1186/1471-2148-7-69 (PMC1885805; doi:10.1186/1471-2148-7-69)
Supplement: Additional file 15 — Log-likelihood values and parameter estimates for human, chimpanzee, rhesus monkey, baboon, dog, mouse, rat, chicken, and zebrafish Ugt1 groups, and human, mouse, and rat Ugt2 groups. Model1 Maximum-likelihood models implemented in the codeml program of the PAML package. M0, one-ratio; M1a, neutral; M2a, selection; M3, discrete; M7, β; M8, β+ω. ℓ2 Estimated log-likelihood values by the codeml program. κ3 Estimated transition/transversion rate ratio by the codeml program. Estimation of Parameters4 ω = KA/KS nonsynonymous/synonymous rate ratio; p = proportion of sites for each site class. M0: one estimated ω for all sites; M1a: estimate p0 = proportion of sites with ω0 = 0, p1 = 1 - p0, proportion of sites with ω1 = 1; M2a: estimate p0 (ω0 = 0), p1 (ω0 = 1), and ω2, p2 = 1 - p0 - p1. M3: estimate p0, p1, ω0, ω1, and ω2; p2 = 1 - p0 - p1. M7: estimates p and q (parameters of β distribution of ω between 0 and 1). M8: same as M7 except additional site class where an estimated ω is allowed. LRT(2Δℓ)5 Statistical likelihood ratio test; comparing the test statistic (2Δℓ) calculated from paired codeml models (M1a vs M2a; M0 vs M3; and M7 vs M8) with the critical value of chi-square asymptotic distribution with appropriate degrees of freedom (i.e. 2 d.f., 4 d.f., and 2 d.f., respectively). 2Δℓ and level of significance are shown for M2a, M3, and M8 models. Positively Selected Sites6 Codon positions predicted to be under positive selection with a posterior probability >0.90 by one codeml model (M2a, M3, or M8), and >0.50 by at least one other model. [file 1471-2148-7-69-S15.pdf]

Additional file 15. Log-likelihood values and parameter estimates for 13 vertebrate *UGT* groups

Human		UGT1				
**Model**[1]	ℓ[2]	κ[3]	**Estimate of Parameters**[4]		**LRT($2\Delta\ell$)**[5]	**Positively Selected Sites**[6]
M0	-4579.946207	1.8171	ω =	-4469.765726	N/A	None
M1a	-4469.765726	2.21556	p_0 =	0.4914	N/A	Not allowed
			p_1 =	0.5086		
M2a	-4467.204119	2.26076	p_0 =	0.48402	5.123214	73 N
			p_1 =	0.48961	p>0.05	85 R
			p_2 =	0.02636		173 P
			ω_2 =	4.34434		
M3	-4461.842346	2.14917	p_0 =	0.40984	236.207722	66 S 67 F 69 S 73 N 74 V 79 S
			p_1 =	0.50296	p<0.001	85 R 89 T 90 Y 96 D 103 G
			p_2 =	0.0872		121 S 173 P 202 V
			ω_0 =	0.05505		
			ω_1 =	0.58631		
			ω_2 =	2.5443		
M7	-4466.625334	2.09458	p =	0.41737	N/A	Not allowed
			q =	0.55151		
M8	-4462.183057	2.16067	p_0 =	0.93622	8.884554	66 S 73 N 74 V 79 S 85 R
			p_1 =	0.06378	p<0.05	89 T 90 Y 96 D 103 G
			p =	0.46916		173 P 202 V
			q =	0.74336		
			ω =	2.80545		

chimpanzee		UGT1				
**Model**[1]	ℓ[2]	κ[3]	**Estimate of Parameters**[4]		**LRT($2\Delta\ell$)**[5]	**Positively Selected Sites**[6]
M0	-4587.579615	1.85035	ω =	0.32954	N/A	None
M1a	-4483.262354	2.25802	p_0 =	0.50276	N/A	Not allowed
			p_1 =	0.49724		
M2a	-4474.178767	2.25802	p_0 =	0.49047	18.167174	69 S 82 F 85 R
			p_1 =	0.49063	p<0.001	90 Y 96 D 173 P
			p_2 =	0.0189		
			ω_2 =	8.44283		
M3	-4468.881555	2.17922	p_0 =	0.42923	237.39612	69 S 74 V 82 F
			p_1 =	0.5333	p<0.001	85 R 90 Y 96 D 173 P
			p_2 =	0.03746		
			ω_0 =	0.06145		
			ω_1 =	0.62704		
			ω_2 =	4.72817		
M7	-4479.23935	2.13036	p =	0.46155	N/A	Not allowed
			q =	0.66339		
M8	-4469.127367	2.19031	p_0 =	0.97269	20.223966	69 S 74 V 82 F
			p_1 =	0.02731	p<0.001	85 R 90 Y 96 D 173 P
			p =	0.49495		
			q =	0.76549		
			ω =	5.60754		

Rhesus monkey		UGT1				
**Model**[1]	ℓ[2]	κ[3]	**Estimate of Parameters**[4]		**LRT(2Δℓ)**[5]	**Positively Selected Sites**[6]
M0	-4699.455941	1.85048	ω =	0.33039	N/A	None
M1a	-4590.841918	2.29792	p_0 =	0.48876	N/A	Not allowed
			p_1 =	0.51124		
M2a	-4582.255511	2.35105	p_0 =	0.47426	17.172814	85 R
			p_1 =	0.51101	p<0.001	173 P
			p_2 =	0.01473		235 S
			ω_2 =	8.56278		
M3	-4575.518425	2.20155	p_0 =	0.38463	247.875032	73 N 79 S 85 R 89 T
			p_1 =	0.57285	p<0.001	90 Y 173 P 202 V 235 S
			p_2 =	0.04253		
			ω_0 =	0.04472		
			ω_1 =	0.58094		
			ω_2 =	3.74773		
M7	-4582.050067	2.18168	p =	0.41253	N/A	Not allowed
			q =	0.56903		
M8	-4573.367194	2.22686	p_0 =	0.98229	17.365746	73 N 79 S 85 R
			p_1 =	0.01771	p<0.001	89 T 173 P 202 V 235 S
			p =	0.4268		
			q =	0.6004		
			ω =	6.30747		

Baboon		UGT1				
**Model**[1]	ℓ[2]	κ[3]	**Estimate of Parameters**[4]		**LRT(2Δℓ)**[5]	**Positively Selected Sites**[6]
M0	-4688.688005	1.84708	ω =	0.31979	N/A	None
M1a	-4581.695245	2.33063	p_0 =	0.48991	N/A	Not allowed
			p_1 =	0.51009		
M2a	-4574.153041	2.36294	p_0 =	0.47904	15.084408	85 R
			p_1 =	0.50889	p<0.001	173 P
			p_2 =	0.01207		202 V
			ω_2 =	9.42254		
M3	-4567.186338	2.20894	p_0 =	0.39301	243.003334	44 G 73 N 79 S 82 F 85 R
			p_1 =	0.5637	p<0.001	90 Y 132 P 173 P 192 I
			p_2 =	0.04329		201 D 202 V 235 S
			ω_0 =	0.04509		
			ω_1 =	0.57327		
			ω_2 =	3.82652		
M7	-4573.069873	2.19115	p =	0.41723	N/A	Not allowed
			q =	0.59358		
M8	-4565.370368	2.22247	p_0 =	0.98331	15.39901	73 N 79 S 82 F 85 R
			p_1 =	0.01669	p<0.001	132 P 173 P 201 D 202 V
			p =	0.43098		
			q =	0.63279		
			ω =	6.54355		

	**Dog**	**UGT1**				
**Model**[1]	ℓ[2]	κ[3]	**Estimate of Parameters**[4]		**LRT($2\Delta\ell$)**[5]	**Positively Selected Sites**[6]
M0	-4524.519175	1.4809	ω =	0.34333	N/A	None
M1a	-4403.464553	1.76763	p_0 =	0.50426	N/A	Not allowed
			p_1 =	0.49574		
M2a	-4399.948839	1.81705	p_0 =	0.49389	7.031428	85 R 87 I 92 K
			p_1 =	0.47215	$p<0.05$	160 G 176 L
			p_2 =	0.03396		
			ω_2 =	5.03228		
M3	-4395.550168	1.7596	p_0 =	0.19346	257.938014	8 V 17 L 18 G 24 H 25 Q 34 A 44 A 47 A 51 L 56 V 59 R 60 R 65 A
			p_1 =	0.44128	$p<0.001$	66 T 68 T 69 S 71 G 72 R 73 G 74 V 76 E 77 N 78 V 79 P 84 R 85 R
			p_2 =	0.36527		86 V 87 I 89 T 90 Y 91 K 92 K 93 V 94 K 95 E 96 D 97 S 98 A 99 L
			ω_0 =	0		100 L 102 S 103 A 105 S 106 H 109 H 111 K 112 E 115 A 116 S 118 A
			ω_1 =	0.23533		121 S 131 L 135 P 139 L 147 F 151 A 153 P 155 S 156 L 157 D 158 F
			ω_2 =	1.21422		159 Q 160 G 167 P 173 A 175 S 176 L 177 N 180 H 184 L 188 K 190 M
						192 I 193 F 195 S 197 S 199 L 202 V 206 P 207 Y 208 E 209 P 217 K
						220 T 222 Q 225 M 227 S 228 A 235 G 239 K 258 A 259 S 262 P
M7	-4399.436303	1.66142	p =	0.38922	N/A	Not allowed
			q =	0.49974		
M8	-4393.857663	1.72149	p_0 =	0.93491	11.15728	65 A 66 T 78 V 85 R 87 I
			p_1 =	0.06509	$p<0.01$	90 Y 92 K 96 D 106 H 160 G 173 A 176 L
		p = 0.43441, q = 0.66237, ω =3.41597				

	**Mouse**	**UGT1**				
**Model**[1]	ℓ[2]	κ[3]	**Estimate of Parameters**[4]		**LRT($2\Delta\ell$)**[5]	**Positively Selected Sites**[6]
M0	-5362.461457	1.74243	ω =	0.37507	N/A	None
M1a	-5251.296323	2.06104	p_0 =	0.5134	N/A	Not allowed
			p_1 =	0.4866		
M2a	-5250.0753	2.11421	p_0 =	0.4977	2.442046	65 A
			p_1 =	0.45687	$p>0.05$	69 E
			p_2 =	0.04544		71 G
			ω_2 =	2.77215		72 R 91 M
M3	-5233.547367	2.04632	p_0 =	0.22884	257.82818	24 Q 25 Q 40 H 44 G 47 S 60 K 64 T 65 A 66 T 68 V 69 E
			p_1 =	0.53115	$p<0.001$	71 G 72 R 73 T 74 A 76 N 77 Q 78 D 79 S 82 F 83 L 84 L
			p_2 =	0.24001		85 R 86 V 88 K 89 I 90 Y 91 M 92 K 93 V 95 R 96 D 98 S
			ω_0 =	0.02385		100 L 105 S 106 H 111 A 112 E 115 A 121 H 142 T 149 L
			ω_1 =	0.35809		151 K 152 L 155 S 158 S 167 L 173 S 188 K 190 V 191 L
			ω_2 =	1.45111		192 L 195 S 201 R 202 V 209 S 227 P 258 L
M7	-5240.642609	1.94832	p =	0.51704	N/A	Not allowed
			q =	0.63706		
M8	-5235.409624	2.04022	p_0 =	0.86729	10.46597	65 A 66 T 69 E 71 G 72 R 73 T
			p_1 =	0.13271	$p<0.01$	74 A 76 N 78 D 79 S 83 L 85 R
			p =	0.65805		86 V 90 Y 91 M 92 K 93 V 96 D
			q =	1.15583		98 S 100 L 112 E 121 H 142 T
			ω =	1.80595		151 K 152 L 192 L 195 S 201 R

Rat		UGT1				
**Model**[1]	ℓ[2]	κ[3]	**Estimate of Parameters**[4]		**LRT($2\Delta\ell$)**[5]	**Positively Selected Sites**[6]
M0	-5472.965706	1.5423	ω =	0.3483	N/A	None
M1a	-5350.67835	1.72435	p_0 =	0.57007	N/A	Not allowed
			p_1 =	0.42993		
M2a	-5348.251714	1.79042	p_0 =	0.54558	4.853272	65 A 66 A 71 G
			p_1 =	0.40942	p>0.05	90 Y 91 N 92 K 100 L
			p_2 =	0.045		
			ω_2 =	3.45562		
M3	-5326.752004	1.74248	p_0 =	0.24616	292.427404	24 Q 25 Q 44 G 47 S 60 N 63 V 64 T 65 A 66 A 69 E
			p_1 =	0.54161	p<0.001	71 G 72 R 73 S 74 V 78 D 79 P 82 F 83 L 85 R 86 V
			p_2 =	0.21224		89 T 90 Y 91 N 92 K 93 V 95 R 98 S 99 M 100 L 102 S
			ω_0 =	0.02401		105 S 106 H 111 A 116 S 131 L 142 S 149 L 150 N 151 A
			ω_1 =	0.34448		155 S 156 L 158 L 173 S 177 N 192 I 195 T 202 V 203 V
			ω_2 =	1.54386		205 S 206 P 208 G 209 S 222 K 235 N 258 L
M7	-5336.803533	1.65271	p =	0.52163	N/A	Not allowed
			q =	0.73587		
M8	-5329.276411	1.73862	p_0 =	0.87595	15.054244	60 N 65 A 66 A 69 E 71 G 73 S
			p_1 =	0.12405	p<0.001	78 D 79 P 83 L 85 R 89 T 90 Y
			p =	0.67894		91 N 92 K 93 V 98 S 100 L 102 S
			q =	1.353		105 S 142 S 158 L 173 S 177 N
			ω =	1.95515		195 T 202 V 203 V 209 S 258 L

Chicken		UGT1				
**Model**[1]	ℓ[2]	κ[3]	**Estimate of Parameters**[4]		**LRT($2\Delta\ell$)**[5]	**Positively Selected Sites**[6]
M0	-6742.092626	2.07232	ω =	0.38713	N/A	None
M1a	-6580.492931	2.31284	p_0 =	0.57999	N/A	Not allowed
			p_1 =	0.42001		
M2a	-6567.258855	2.46347	p_0 =	0.54945	26.468152	69 A 71 L 84 E 87 H 92 K
			p_1 =	0.40166	p<0.001	93 V 95 R 98 D 106 W
			p_2 =	0.0489		130 V 173 A 203 I 222 I
			ω_2 =	4.74842		
M3	-6555.365391	2.31977	p_0 =	0.34388	373.45447	37 A 40 N 47 E 65 D 66 N 69 A 71 L 72 K
			p_1 =	0.49129	p<0.001	74 A 76 E 78 G 84 E 85 Q 87 H 88 R 89 L
			p_2 =	0.16483		90 Q 92 K 93 V 94 K 95 R 96 L 98 D 99 V
			ω_0 =	0.06109		102 I 103 S 106 W 109 K 121 N
			ω_1 =	0.46493		130 V 158 F 173 A 193 D 195 S 197 L
			ω_2 =	1.7753		199 L 201 D 202 F 203 I 205 K 222 I
M7	-6569.82083	2.20258	p =	0.50568	N/A	Not allowed
			q =	0.68377		
M8	-6552.635115	2.33776	p_0 =	0.9276	34.37143	40 N 69 A 71 L 84 E 87 H 90 Q
			p_1 =	0.0724	p<0.001	92 K 93 V 95 R 98 D 106 W
			p =	0.59799		130 V 173 A 202 F 203 I 222 I
			q =	0.94708		
			ω =	2.97901		

Zebrafish		UGT1A				
Model[1]	ℓ[2]	κ[3]	Estimate of Parameters[4]		LRT($2\Delta\ell$)[5]	Positively Selected Sites[6]
M0	-4318.270545	1.42374	ω =	0.28891	N/A	None
M1a	-4246.095061	1.53385	p_0 =	0.72087	N/A	Not allowed
			p_1 =	0.27913		
M2a	-4244.528178	1.56154	p_0 =	0.71681	3.133766	66 N 70 E
			p_1 =	0.26392	p>0.05	101 S
			p_2 =	0.01928		173 Y
			ω_2 =	4.0526		202 S
M3	-4238.240721	1.51694	p_0 =	0.44515	160.059648	57 S 64 A 65 E 66 N 67 L 69 K
			p_1 =	0.45953	p<0.001	70 E 72 Q 73 N 84 T 88 V 91 R
			p_2 =	0.09532		94 L 95 Q 97 L 101 S 173 Y
			ω_0 =	0.07075		197 G 202 S 206 S 258 V
			ω_1 =	0.39859		
			ω_2 =	1.79333		
M7	-4246.880684	1.48292	p =	0.60474	N/A	Not allowed
			q =	1.23665		
M8	-4238.036997	1.51677	p_0 =	0.92152	17.687374	57 S 65 E 66 N 67 L 70 E 73 N
			p_1 =	0.07848	p<0.001	84 T 88 V 91 R 94 L 97 L
			p =	0.90846		101 S 173 Y 202 S 206 S 258 V
			q =	2.62772		
			ω =	1.9508		

Zebrafish		UGT1B				
Model[1]	ℓ[2]	κ[3]	Estimate of Parameters[4]		LRT($2\Delta\ell$)[5]	Positively Selected Sites[6]
M0	-3821.397591	1.60209	ω =	0.25823	N/A	None
M1a	-3754.061545	1.92987	p_0 =	0.6209	N/A	Not allowed
			p_1 =	0.3791		
M2a	-3754.061545	1.92987	p_0 =	0.6209	0	
			p_1 =	0.23285	p>0.05	
			p_2 =	0.14626		
			ω_2 =	1		
M3	-3746.892296	1.88372	p_0 =	0.38952	149.01059	40 L 59 S 61 E 62 Q 63 L 66 S 67 L 69 K 70 G 73 Q
			p_1 =	0.40644	p<0.001	75 V 76 F 77 T 79 S 81 A 84 M 85 D 87 F 88 V 89 Q
			p_2 =	0.20404		90 L 91 E 94 L 96 F 97 T 99 S 100 Q 101 V 102 E
			ω_0 =	0.03238		104 C 153 P 159 L 176 G 182 N 190 F 194 G 198 V
			ω_1 =	0.30138		199 L 202 V 205 A 206 S 213 R 221 Y 250 V 251 F
			ω_2 =	1.15784		258 Q 260 S
M7	-3748.453462	1.83922	p =	0.44133	N/A	Not allowed
			q =	0.89796		
M8	-3747.027755	1.87853	p_0 =	0.84435	2.851414	40 L 59 S 62 Q 63 L 66 S 67 L 70 G 73 Q
			p_1 =	0.15565	p>0.05	75 V 76 F 77 T 79 S 81 A 85 D 88 V 89 Q
			p =	0.59311		90 L 91 E 94 L 99 S 100 Q 101 V 102 E
			q =	2.16612		153 P 159 L 176 G 190 F 198 V 199 L
			ω =	1.22287		202 V 205 A 206 S 213 R 221 Y 250 V 251 F

**Human**	**UGT2**					
**Model**[1]	ℓ[2]	κ[3]	**Estimate of Parameters**[4]		**LRT(2Δℓ)**[5]	**Positively Selected Sites**[6]
M0	-6378.440841	2.03159	ω =	0.3792	N/A	None
M1a	-6223.085308	2.15207	p_0 =	0.5887	N/A	Not allowed
			p_1 =	0.4113		
M2a	-6216.57934	2.27307	p_0 =	0.57797	13.011936	8 M 45 S 47 N 71 F
			p_1 =	0.38053	$p<0.01$	78 N 79 R 97 K 98 D
			p_2 =	0.0415		100 H103 S 133 V 329 N
			ω_2 =	3.58045		417 S
M3	-6214.925999	2.24208	p_0 =	0.47633	327.029684	8 M 10 G 43 P 45 S 47 N 64 I 67 V 71 F 73 L 75 W 76 L 78 N 79 R
			p_1 =	0.40777	$p<0.001$	81 S 87 R 88 F 90 Q 96 I 97 K 98 D 99 F 100 H 102 V 103 S 104 Q
			p_2 =	0.1159		112 K 118 A 128 L 133 V 145 G 153 R 156 P 158 S 160 V 197 Y 206 T
		ω_0 = 0.03973, ω_1 = 0.63079, ω_2 = 2.25067				207 L 209 W 329 N 377 A 398 N 417 S 422 A
M7	-6225.012005	2.10748	p =	0.21195	N/A	Not allowed
			q =	q= 0.29526		
M8	-6214.456244	2.24394	p_0 =	0.91863	21.111522	8 M 10 G 21 D 25 K 35 A 37 G 39 L 40 F 41 I 43 P 44 T 45 S 47 N
			p_1 =	0.08137	$p<0.001$	52 F 56 R 57 V 62 E 63 R 64 I 66 G 67 V 69 K 70 D 71 F 73 L 75 W
			p =	0.27687		76 L 77 E 78 N 79 R 80 P 81 S 82 P 87 R 88 F 90 Q 92 M 93 A 95 V
			q =	0.49562		96 I 97 K 98 D 99 F 100 H 101 M 102 V 103 S 104 Q 106 I 110 V
			ω =	2.57616		112 K 118 A 128 L 130 S 133 V 134 F 142 L 143 K 145 G 153 R 156 P
						158 S 159 T 160 V 163 H 167 V 169 Y 196 S 197 Y 206 T 207 L 209 W
						211 S 214 S 248 R 250 Y 293 V 296 L 303 L 325 A 329 N 377 A 394 E
						395 V 398 N 403 V 417 S 422 A

**Mouse**	**UGT2**					
**Model**[1]	ℓ[2]	κ[3]	**Estimate of Parameters**[4]		**LRT(2Δℓ)**[5]	**Positively Selected Sites**[6]
M0	-7758.999805	1.66263	ω =	0.27134	N/A	None
M1a	-7593.014406	1.80026	p_0 =	0.6882	N/A	Not allowed
			p_1 =	0.3118		
M2a	-7592.975076	1.80659	p_0 =	0.68775	0.07866	159 T
			p_1 =	0.31115	$p>0.5$	
			p_2 =	0.0011		
			ω_2 =	4.46072		
M3	-7584.144422	1.73597	p_0 =	0.54903	349.710766	8 M 10 G 24 L 25 R 39 L 43 P 44 S 45 S 47 S 51 T 60 G 63 K 64 I
			p_1 =	0.31668	$p<0.001$	66 S 67 V 69 K 71 F 73 L 74 T 75 W 79 R 87 T 90 K 91 E 95 V 97 E
			p_2 =	0.13429		99 F 101 L 102 V 112 K 115 K 118 S 130 S 133 V 145 G 155 S 159 T
			ω_0 =	0.06086		160 V 184 Q 196 S 197 Y 199 M 209 W 211 Q 214 S 219 A 227 C
			ω_1 =	0.4633		250 Y 394 E 398 N 458 V
			ω_2 =	1.14667		
M7	-7588.299719	1.69517	p =	0.40064	N/A	Not allowed
			q =	0.8832		
M8	-7584.514194	1.73022	p_0 =	0.81062	7.57105	47 S 69 K 71 F 73 L
			p_1 =	0.18938	$p<0.5$	74 T 97 E 99 F
		p = 0.68851, q = 3.18426, ω = 1.00774				159 T 160 V 211 Q

**Rat**	**UGT2**					
**Model**[1]	ℓ[2]	κ[3]	**Estimate of Parameters**[4]		**LRT(2$\Delta\ell$)**[5]	**Positively Selected Sites**[6]
M0	-8858.273247	1.68898	ω =	0.29892	N/A	None
M1a	-8593.840049	1.8352	p_0 =	0.63102	N/A	Not allowed
			p_1 =	0.36898		
M2a	-8593.670226	1.84778	p_0 =	0.6298	0.339646	211 Q
			p_1 =	0.36229	p>0.5	
			p_2 =	0.0079		
			ω_2 =	2.32473		
M3	-8582.004433	1.78155	p_0 =	0.43316	552.537628	8 M 10 G 16 V 21 D 24 L 25 R 34 V 35 A 38 A 39 L 42 T 43 P 45 S
			p_1 =	0.30836	p<0.001	46 V 47 S 49 S 51 T 54 I 57 V 58 P 59 F 60 G 61 K 62 E 63 K 64 I
			p_2 =	0.25848		66 S 67 V 69 K 70 D 71 F 73 L 74 T 75 W 76 L 77 E 79 R 81 S 82 P
			ω_0 =	0.03241		83 S 85 I 87 T 88 F 89 Y 90 K 91 E 92 M 93 A 94 K 95 V 96 I 97 E
			ω_1 =	0.31256		98 E 99 F 102 V 103 S 104 R 105 G 106 I 108 D 109 G 110 V 111 L
			ω_2 =	1.03414		112 K 115 K 118 T 122 R 123 G 124 K 128 L 129 L 130 S 134 F 143 K
						145 G 155 S 156 P 158 S 159 T 160 V 166 K 167 V 169 F 176 A 182 T
						184 Q 188 A 196 S 197 Y 199 M 205 E 206 T 209 W 211 Q 214 S 219 A
						226 L 227 C 231 G 250 Y 294 K 303 L 320 K 325 A 333 L 334 F 380 P
						387 K 394 E 395 V 398 N 403 A 410 R 411 A 458 V 460 A
M7	-8585.621695	1.73674	p =	0.31242	N/A	Not allowed
			q =	0.5888		
M8	-8581.130328	1.78311	p_0 =	0.85585	8.982734	47 S 69 K 71 F 74 T 90 K
			p_1 =	0.14415	p<0.5	91 E 97 E 102 V 105 G 197 Y
			p =	0.41843		206 T 209 W 211 Q 394 E
			q =	1.32916		
			ω =	1.22911		

Model[1] Maximum likelihood models implemented in the codeml program of the PAML package.

M0, one-ratio; M1a, neutral; M2a, selection; M3, discrete; M7, β; M8, β+ω.

ℓ[2] Estimated log-likelihood values by the codeml program.

κ[3] Estimated transition/transversion rate ratio by the codeml program.

Estimation of Parameters[4] ω=K_A/K_S nonsynonymous/synonymous rate ratio; p=proportion of sites for each site class. M0: one estimated ω for all sites; M1a: estimate p_0=proportion of sites with ω_0=0, p_1=1 - p_0, proportion of sites with ω_1=1; M2a: estimate p_0 (ω_0=0), p_1 (ω_0=1), and ω_2, p_2=1 - p_0 - p_1. M3: estimate p_0, p_1, ω_0, ω_1, and ω_2; p_2=1 - p_0 - p_1. M7: estimate p and q (parameters of β distribution of ω between 0 and 1). M8: same as M7 except additional site class where an estimated ω is allowed.

LRT(2$\Delta\ell$)[5] Statistical likelihood ratio test; comparing the test statistic (2$\Delta\ell$) calculated from paired codeml models (M1a vs M2a; M0 vs M3; and M7 vs M8) with the critical value of chi-square asymptotic distribution with appropriate degrees of freedom (i.e. 2 d.f., 4 d.f., and 2 d.f., respectively). 2$\Delta\ell$ and level of significance are shown for M2a, M3, and M8 models.

Positively Selected Sites[6] Codon positions predicted to be under positive selection with a posterior probability >0.90 by one codeml model (M2a, M3, or M8), and >0.50 by at least one other model.
