# Supplementary material for: Adaptive evolution of multiple-variable exons and structural diversity of drug-metabolizing enzymes
Source: BMC Evol Biol. 2007 May 2;7:69. doi: 10.1186/1471-2148-7-69 (PMC1885805; doi:10.1186/1471-2148-7-69)
Supplement: Additional file 16 — Summary information for Ugt1 and Ugt2 groups analyzed. Tree length1 Measured as the number of nt substitutions along the tree per codon by the codeml program. The ω+ sites2 Codon positions predicted to be under positive selection with a posterior probability >0.90 by one codeml model (M2a, M3, or M8), and >0.50 by at least one other model. For Ugt1 sequences, we used only the variable exons. For Ugt2 sequences, we used full-length sequences but excluded Ugt2a2 because it shares five constant exons with Ugt2a1. [file 1471-2148-7-69-S16.pdf]

Additional file 16. Summary information for 13 vertebrate *UGT* groups analyzed by the codeml

UGT1	Number of exons	ω	Tree length	Number of ω+ sites	Positively selected ω+ sites
Human	9	0.33887	6.3116	3	73 N 85 R 173 P
chimpanzee	9	0.32954	6.37188	4	85 R 90 Y 96 D 173 P
Rhesus monkey	10	0.33039	6.43642	3	85 R 173 P 235 S
Baboon	10	0.31979	6.51735	3	85 R 173 P 202 V
Dog	10	0.34333	6.26742	12	65 A 66 T 78 V 85 R 87 I 90 Y 92 K 96 D 106 H 160 G 173 A 176 L
Mouse	9	0.37507	7.23936	27	65 A 66 T 69 E 71 G 72 R 73 T 74 A 76 N 78 D 79 S 83 L 85 R 86 V 90 Y 91 M 92 K 93 V 96 D 98 S 112 E 121 H 142 T 151 K 152 L 192 L 195 S 201 R
Rat	8	0.3483	7.42187	26	60 N 65 A 66 A 69 E 71 G 73 S 78 D 79 P 85 R 89 T 90 Y 91 N 92 K 93 V 98 S 100 L 105 S 142 S 158 L 173 S 177 N 195 T 202 V 203 V 209 S 258 L
Chicken	13	0.38713	7.97652	16	40 N 69 A 71 L 84 E 87 H 90 Q 92 K 93 V 95 R 98 D 106 W 130 V 173 A 202 F 203 I 222 I
Zebrafish a	7	0.28891	5.23699	9	65 E 66 N 70 E 88 V 91 R 101 S 173 Y 202 S 206 S
Zebrafish b	6	0.25823	5.63854	9	62 Q 88 V 89 Q 91 E 99 S 101 V 205 A 206 S 250 V

UGT2	Number of exons	ω	Tree length[1]	Number of ω+ sites[2]	Positively selected ω+ sites
Human	9	0.37917	2.9105	11	8 M 45 S 47 N 78 N 79 R 97 K 98 D 100 H103 S 329 N 417 S
Mouse	9	0.27134	4.15942	10	47 S 69 K 71 F 73 L 74 T 97 E 99 F 159 T 160 V 211 Q
Rat	10	0.29892	5.02292	14	47 S 69 K 71 F 74 T 90 K 91 E 97 E 102 V 105 G 197 Y 206 T 209 W 211 Q 394 E

Tree length[1] Measured as the number of nucleotide substitutions along the tree per codon by the codeml program.

The ω+ sites[2] Codon positions predicted to be under positive selection with a posterior probability >0.90 by one codeml model (M2a, M3, or M8), and >0.50 by at least one other model.

For *Ugt1* sequences, we used only the variable exons. For *Ugt2* sequences, we used full-length sequences but excluded *Ugt2a2* because it shares five constant exons with *Ugt2a1*.
