# Supplementary material for: Adaptive evolution of multiple-variable exons and structural diversity of drug-metabolizing enzymes
Source: BMC Evol Biol. 2007 May 2;7:69. doi: 10.1186/1471-2148-7-69 (PMC1885805; doi:10.1186/1471-2148-7-69)
Supplement: Additional file 17 — Alignment of the protein sequences of UGT71G1 to those of the human (Additional file 17), chimpanzee (Additional file 18), rhesus monkey (Additional file 19), baboon (Additional file 20), dog (Additional file 21), mouse (Additional file 22), rat (Additional file 23), chicken (Additional file 24), and zebrafish clusters A (Additional file 25) and B (Additional file 26) variable UGT1 sequences, as well as the corresponding human (Additional file 27), mouse (Additional file 28), and rat (Additional file 29) UGT2 proteins. Each vertebrate UGT group was aligned by ClustalW and then aligned with UGT71G1 according to the structural alignment shown in the Figure 4A. The ω+ sites predicted to be subject to positive selection with a posterior probability >0.90 by one model and >0.5 by at least one other model are highlighted in red. [file 1471-2148-7-69-S17.pdf]

Additional file 17. Alignment of the human (h) UGT1 variable sequences with those of the UGT71G1

```
Ugt71g1    1                    MSMSDINKNSELIFIPAPGIGHLASALEFAKLLTNHDKNLY
ha8        1 MARTGWTSPIP.LCVSLLLTC..GFAEAGKLLVVP.MDGSHWFTMQSVVEKLILRGHEVV
ha9        1 MACTGWTSPLP.LCVCLLLTC..GFAEAGKLLVVP.MDGSHWFTMRSVVEKLILRGHEVV
ha10       1 MARAGWTSPVP.LCVCLLLTC..GFAEAGKLLVVP.MDGSHWFTMQSVVEKLILRGHEVV
ha7        1 MARAGWTGLLP.LYVCLLLTC..GFAKAGKLLVVP.MDGSHWFTMQSVVEKLILRGHEVV
ha3        1 MATGLQVPLPWLATGLLLLLSVQPWAESGKVLVVP.IDGSHWLSMREVLRELHARGHQAV
ha5        1 MATGLQVPLPQLATGLLLLLSVQPWAESGKVLVVP.TDGSHWLSMREALRDLHARGHQVV
ha4        1 MARGLQVPLPRLATGLLLLLSVQPWAESGKVLVVP.TDGSPWLSMREALRELHARGHQAV
ha1        1 MAVESQGGRP.LVLGLLLCVLGPVVSHAGKILLIP.VDGSHWLSMLGAIQQLQQRGHEIV
ha6        1 MACLLR.SFQRISAGVFF.LALWGMVVGDKLLVVP.QDGSHWLSMKDIVEVLSDRGHEIV

Ugt71g1   42 ITVFCIKFPGMPFADSYIKSVLASQPQIQLIDLPEVEPPPQELLKSPEFYILTFLESLIP
ha8       57 VVMPEV.....SWQLGKSLNCTV..KTYSTSYTLE.....DLDREFMDFADAQWKAQ.VR
ha9       57 VVMPEV.....SWQLGRSLNCTV..KTYSTSYTLE.....DLDREFKAFAHAQWKAQ.VR
ha10      57 VVMPEV.....SWQLERSLNCTV..KTYSTSYTLE.....DQNREFMVFAHAQWKAQ.AQ
ha7       57 VVMPEV.....SWQLGRSLNCTV..KTYSTSYTLE.....DQDREFMVFADARWTAP.LR
ha3       60 VLTPEV.....NMHIKEENFFTL..TTYAISWTQD.....EFDRHVLGHTQLYFETEHFL
ha5       60 VLTLEV.....NMYIKEENFFTL..TTYAISWTQD.....EFDRLLLGHTQSFFETEHLL
ha4       60 VLTPEV.....NMHIKEEKFFTL..TAYAVPWTQK.....EFDRVTLGYTQGFFETEHLL
ha1       59 VLAPDA.....SLYIRDGAFYTL..KTYPVPFQRE.....DVKESFVSLGHNVFENDSFL
ha6       58 VVVPEV.....NLLLKESKYYTR..KIYPVPYDQE.....ELKNRYQSFGNNHFAERSFL

Ugt71g1  102 HVKATIKTILSNK.........................VVGLVLDFFCVSMIDVGNEFGI
ha8      104 SLFSLFLSSSNGFFNLFFSHCRSLFNDRKLVEYLKESSFDAVFLDPFDACGLIVAKYFSL
ha9      104 SIYSLLMGSYNDIFDLFFSNCRSLFKDKKLVEYLKESSFDAVFLDPFDNCGLIVAKYFSL
ha10     104 SIFSLLMSSSSGFLDLFFSHCRSLFNDRKLVEYLKESSFDAVFLDPFDTCGLIVAKYFSL
ha7      104 SAFSLLTSSSNGIFDLFFSNCRSLFKDKKLVEYLKESCFDAVFLDPFDACGLIVAKYFSL
ha3      108 KKFFRSMAMLNNMSLVYHRSCVELLHNEALIRHLNATSFDVVLTDPVNLCAAVLAKYLSI
ha5      108 MKFSRRMAIMNNMSLIIHRSCVELLHNEALIRHLHATSFDVVLTDPFHLCAAVLAKYLSI
ha4      108 KRYSRSMAIMNNVSLALHRCCVELLHNEALIRHLNATSFDVVLTDPVNLCGAVLAKYLSI
ha1      107 QRVIKTYKKIKKDSAMLLSGCSHLLHNKELMASLAESSFDVMLTDPFLPCSPIVAQYLSL
ha6      106 TAPQTEYRNNMIVIGLYFINCQSLLQDRDTLNFFKESKFDALFTDPALPCGVILAEYLGL

Ugt71g1  137 PSYLFLTSNVGFLSLMLSLKNRQIEEVFDDSDRDHQLLNIPGISNQVPSNVLPDACFNKD
ha8      164 PSVVFARGIACHYLEEGAQCPAPLSYVPRILLGFSDAMTFKERVRNHIMHLEEHLFCQYF
ha9      164 PSVVFARGILCHYLEEGAQCPAPLSYVPRILLGFSDAMTFKERVRNHIMHLEEHLLCHRF
ha10     164 PSVVFTRGIFCHHLEEGAQCPAPLSYVPNDLLGFSDAMTFKERVWNHIVHLEDHLFCQYL
ha7      164 PSVVFARGIFCHYLEEGAQCPAPLSYVPRLLLGFSDAMTFKERVRNHIMHLEEHLFCPYF
ha3      168 PTVFFLRNIPCDLDFKGTQCPNPSSYIPRLLTTNSDHMTFMQRVKNMLYPLALSYICHAF
ha5      168 PAVFFLRNIPCDLDFKGTQCPNPSSYIPRLLTTNSDHMTFLQRVKNMLYPLALSYLCHAV
ha4      168 PAVFFWRYIPCDLDFKGTQCPNPSSYIPKLLTTNSDHMTFLQRVKNMLYPLALSYICHTF
ha1      167 PTVFFLHALPCSLEFEATQCPNPFSYVPRPLSSHSDHMTFLQRVKNMLIAFSQNFLCDVV
ha6      166 PSVYLFRGFPCSLEHTFSRSPDPVSYIPRCYTKFSDHMTFSQRVANFLVNLLEPYLFYCL

Ugt71g1  197 GGYIAYYKLAERFRDTKGIIVNTFSDLEQSSIDALYDHDEK.IPPIYAVGPLLDLKGQPNPK
ha8      224 SKNALEIASEILQTPVTAYDLYSHTSIWLLRTDFVLDYPKPVMPNMIFIGGINCHQGKPLPM
ha9      224 FKNALEIASEILQTPVTEYDLYSHTSIWLLRTDFVLDYPKPVMPNMIFIGGINCHQGKPLPM
ha10     224 FRNALEIASEILQTPVTAYDLYSHTSIWLLRTDFVLDYPKPVMPNMIFIGGINCHQGKPLPM
ha7      224 FKNVLEIASEILQTPVTAYDLYSHTSIWLLRTDFVLEYPKPVMPNMIFIGGINCHQGKPVPM
ha3      228 SAPYASLASELFQREVSVVDILSHASVWLFRGDFVMDYPRPIMPNMVFIGGINCANRKPLSQ
ha5      228 SAPYASLASELFQREVSVVDLVSHASVWLFRGDFVMDYPRPIMPNMVFIGGINCANGKPLSQ
ha4      228 SAPYASLASELFQREVSVVDLVSYASVWLFRGDFVMDYPRPIMPNMVFIGGINCANGKPLSQ
ha1      227 YSPYATLASEFLQREVTVQDLLSSASVWLFRSDFVKDYPRPIMPNMVFVGGINCLHQNPLSQ
ha6      226 FSKYEELASAVLKRDVDIITLYQKVSVWLLRYDFVLEYPRPVMPNMVFIGGINCKKRKDLSQ
```
