# Supplementary material for: Adaptive evolution of multiple-variable exons and structural diversity of drug-metabolizing enzymes
Source: BMC Evol Biol. 2007 May 2;7:69. doi: 10.1186/1471-2148-7-69 (PMC1885805; doi:10.1186/1471-2148-7-69)
Supplement: Additional file 18 — Alignment of the protein sequences of UGT71G1 to those of the human (Additional file 17), chimpanzee (Additional file 18), rhesus monkey (Additional file 19), baboon (Additional file 20), dog (Additional file 21), mouse (Additional file 22), rat (Additional file 23), chicken (Additional file 24), and zebrafish clusters A (Additional file 25) and B (Additional file 26) variable UGT1 sequences, as well as the corresponding human (Additional file 27), mouse (Additional file 28), and rat (Additional file 29) UGT2 proteins. Each vertebrate UGT group was aligned by ClustalW and then aligned with UGT71G1 according to the structural alignment shown in the Figure 4A. The ω+ sites predicted to be subject to positive selection with a posterior probability >0.90 by one model and >0.5 by at least one other model are highlighted in red. [file 1471-2148-7-69-S18.pdf]

Additional file 18. Alignment of the chimpanzee (c) UGT1 variable sequences with those of the UGT71G1

```
Ugt71g1    1                    MSMSDINKNSELIFIPAPGIGHLASALEFAKLLTNHDKNLY
ca8        1 MARTGWTSPLPLCVSLMLTC...GFAEAGKLLVVP.MDGSHWFTMQSVVEKLILRGHEVV
ca9        1 MACTGWTSPLPLCVCLLLTC...GFAEAGKLLVVP.MDGSHWFTMRSVVEKLILRGHEVV
ca10       1 MACAGWTSPVPLCVCLLLTC...GFAEAGKLLVVP.MDGSHWFTMQSVVEKLILRGHEVV
ca7        1 MARAGWTGLLPLYVCLLLTC...GFAKAGKLLVVP.MDGSHWFTMQSVVEKLILRGHEVV
ca2        1 MATGPQVPLLQLATGVLLLLSVQPWAESGKVLVVP.IDGSHWLSMREAVRELHARGHQAV
ca4        1 MARGLQVPLPRLATGVLLLLSVQPWAESGKVLVVP.TDGSHWLSMREALRELHARGHQAV
ca3        1 MATGLQVPLPRLATGLLLLLSVQPWAESGKVLVVP.TDGSHWLSMREALRELHARGHQAV
ca1        1 MAVESQGGRP.LVLGLLLCVLGPVVSHAGKILLIP.VDGSHWLSMLGAIQQLQQRGHEIV
ca6        1 MACLLRA.FQRISAGVFF.LALWGMVVGDKLLVVP.QDGSHWLSMKDIVEVLSDRGHEIV

Ugt71g1   42 ITVFCIKFPGMPFADSYIKSVLASQPQIQLIDLPEVEPPPQELLKSPEFYILTFLESLIP
ca8       57 VVMPEV.....SWQLGKSLNCTV..KTYSTSYTLE.....DLDREFMDFADAQWKAQ.VQ
ca9       57 VVMPEV.....SWQLGRSLNCTV..KTYSTSYTLE.....DLDREFKAFAHAQWKAQ.VR
ca10      57 VVMPEV.....SWQLERSLNCTV..KTYSTSYTLE.....DQNREFMVFAHAQWKAQ.AQ
ca7       57 VVMPEV.....SWQLGRSLNCTV..KTYSTSYTLE.....DQDREFMVFADARWTAP.LR
ca2       60 VLTPEV.....NMHIKEEKFFTL..TTYAISWTQD.....EFDRLVLGHTQLYFETEHFL
ca4       60 ALTPEV.....NMHIKEENFFTL..TTYAISWTQD.....EFDRLLLGHTQSFFETEHLL
ca3       60 VLTPEV.....NMHIKEENFFTL..TTYAISWTQD.....EFDRHVLDHTQLYFETEHFL
ca1       59 VLAPDA.....SLYIRDGAFYTL..KTYPVPFQRE.....DVKESFVSLGHNVFENDSFL
ca6       58 VVVPEV.....NLLLKESKYYTR..KIYPVPYDQE.....ELKNRYQSFGNNHFAERSSL

Ugt71g1  102 HVKATIKTILSNK.........................VVGLVLDFFCVSMIDVGNEFGI
ca8      104 SLFSLFLSSSNGFFDLFFSHCRSLFNDRKLVEYLKESSFDAVFLDPFDACGLIVAKYFSL
ca9      104 SIYSLLMGSYNDIFDLFFSNCRSLFKDKKLVEYLKESSFDAVFLDPFDNCGLIVAKYFSL
ca10     104 SIFSLLMSSSSDFLDLFFSHCRSLFNDRKLVEYLKESSFDAVFLDPFDTCGLIVAKYFSL
ca7      104 SAFSLLTSSSNGIFDLFFSNCRSLFKDKKLVEYLKESSFDAVFLDPFDACGLIVAKYFSL
ca2      108 KTFSKSMAILKNSSLVLHRSCVELLHNEALIRHLNATSFDVVLIDPIYLCGAVLAKYLSI
ca4      108 KRYSRSVAIMNNVSLVLHRSCVELLHNEALIRHLNATYFDVVLTDPFHLCGAVVAKYLSI
ca3      108 KKFFRSMAMLNNMSLVYHRSCVELLHNEALIRHLNAISFDVVLTDPVNLCAAVLAKYLSI
ca1      107 QRVIKTYKKIKKDSAMLLSGCSHLLHNKELMASLAESSFDVMLTDPFLPCSPIVAQYLSL
ca6      106 TAPQTEYRNNMIVIGLYFINCQSLLQDRDTLNFFKESKFDALFTDPALPCGVILAEYLGL

Ugt71g1  137 PSYLFLTSNVGFLSLMLSLKNRQIEEVFDDSDRDHQLLNIPGISNQVPSNVLPDACFNKD
ca8      164 PSVVFARGIACHYLEEGAQCPAPLSYVPRILLGFSDAMSFKERVRNHIMHLEEHLFCQYF
ca9      164 PSVVFTRGILCHYLEEGAQCPAPLSYVPRILLGFSDAMTFKEKVRNHIMHLEEHLLCHRF
ca10     164 PSVVFTRGIFCHHLEEGAQCPAPLSYVPNDLLGFSDAMTFKERVWNHIVHLEDHLSCQYL
ca7      164 PSVVFARGIFCHYLEEGAQCPAPLSYIPRLLLGFSDAMTFKERVWNHIMHLEEHLFCPYF
ca2      168 PAVFFLRNIPCDLDFKGTQCPNPYSYIPKLLTTNSDHMTFLQRVKNMLYPLALSYLCHAL
ca4      168 PAVFFLRNIPCDLDFKGTQCPNPYSYIPKYLTTNSDHMTFLQRVKNMLYPLALSYLCHAL
ca3      168 PTVFFLRNIPCDLDFKGTQCPNPSSYIPRLLTTNSDHMTFVQRVKNMLYPLALSYICHAF
ca1      167 PTVFFLHALPCSLEFEATQCPNPFSYVPRPLSSHSDHMTFLQRVKNMLIAFSQNFLCDVV
ca6      166 PSVYLFRGFPCSLEHTFSRSPDPVSYIPRCYTKFSDHMTFSQRVANFLVNLLEPYLFYCL

Ugt71g1  197 GGYIAYYKLAERFRDTKGIIVNTFSDLEQSSIDALYDHDEK.IPPIYAVGPLLDLKGQPNPK
ca8      224 FKNALEIASEILQTPVTAYDLYSHTSIWLLRTDFVLDYPKPVMPNMIFIGGINCHQGKPLPM
ca9      224 FKNALEIASEILQTPVTEYDLYSHTSIWLLRTDFVLDYPKPVMPNMIFIGGINCHQGKPLPM
ca10     224 FRNALEIASEILQTPVTAYDLYSHTSIWLLRTDFVLDYPKPVMPNMIFIGGINCHQGKPLPM
ca7      224 FKNVLEIASEILQTPVTAYDLYSHTSIWLLRTDFVLEYPKPVMPNMIFIGGINCHQGKPVPM
ca2      228 SAPYASLASELFQREVSVVDLLSHASVWLFRWDFVMEYPRPIMPNMVFIGGINCANRKPLSQ
ca4      228 SAPYASLASELFQREVSVVDLVSHASVWLFRRDFVMDYPRPIMPNMVFIGGINCANRKPLSQ
ca3      228 SAPYASLASELFQREVSVVDILSHASVWLFRGDFVMDYPRPIMPNMVFIGGVNCASRKPLSQ
ca1      227 YSPYATLASEFLQREVTVQDLLSSASVWLFRSDFVKDYPRPIMPNMVFVGGINCLHQNPLSQ
ca6      226 FSKYEELASAVLKRDVDIITLYQKVSVWLLRYDFVLEYPRPVMPNMVFIGGINCKKRKDLSQ
```
