# Supplementary material for: Adaptive evolution of multiple-variable exons and structural diversity of drug-metabolizing enzymes
Source: BMC Evol Biol. 2007 May 2;7:69. doi: 10.1186/1471-2148-7-69 (PMC1885805; doi:10.1186/1471-2148-7-69)
Supplement: Additional file 19 — Alignment of the protein sequences of UGT71G1 to those of the human (Additional file 17), chimpanzee (Additional file 18), rhesus monkey (Additional file 19), baboon (Additional file 20), dog (Additional file 21), mouse (Additional file 22), rat (Additional file 23), chicken (Additional file 24), and zebrafish clusters A (Additional file 25) and B (Additional file 26) variable UGT1 sequences, as well as the corresponding human (Additional file 27), mouse (Additional file 28), and rat (Additional file 29) UGT2 proteins. Each vertebrate UGT group was aligned by ClustalW and then aligned with UGT71G1 according to the structural alignment shown in the Figure 4A. The ω+ sites predicted to be subject to positive selection with a posterior probability >0.90 by one model and >0.5 by at least one other model are highlighted in red. [file 1471-2148-7-69-S19.pdf]

Additional file 19. Alignment of the rhesus monkey (*Macaca mulatta* [mma]) UGT1 variable sequences with those of the UGT71G1

```
Ugt71g1    1                    MSMSDINKNSELIFIPAPGIGHLASALEFAKLLTNHDKNLY
mmaa10     1 MAHTRCISPVPLCLCLLLTCG...FAEAGKLLVVP.MDGSHWFTMQSVVEKLILRGHEVV
mmaa8      1 MARTRWTSPVLLCVSLLLTCG...FAEAGKLLVVP.MDGSHWFTMQSVVEKLILRGHEVV
mmaa7      1 MACAGWTGLLPLSVCLLLTCG...FAEAGKLLVVP.MDGSHWFTMRSVVEKLILRGHEVV
mmaa9      1 MTRTGWSSPLPLCVCLLLACG...FAEAGKLLVVP.VDGSHWLSMRSVVEKLILRGHEVV
mmaa4a     1 MATGLQVPLTRLATGLLLLLSVQPWAESGKVLVVP.TDGSHWLSMREAVRELHARGHQAV
mmaa4b     1 MATGLQVPLPQLATGLLLLLSVQPWAESGKVLVVP.TDGSHWLSMREAVRELHARGHQAV
mmaa5      1 MATGLQVSLPQLATGLLLLLSVQPWAESGKVLVVP.TDGSHWLSMREAVRELHARGHQVV
mmaa2      1 MATGPQVPLLWLATGLLLLLSVQPWAESGKVLVVP.IDGSHWLSMREAVRELHARGHQAV
mmaa1      1 MAVESQGRHP.LVLGLLLCVLGPVLCHAGKMLLIP.VDGSHWLSMLGTIQQLQQRGHEIV
mmaa6      1 ..MAYLLRAFQRISAEVFFLALWGMVVGDKLLVIP.QDGSHWLSMKDIVEVLSDRGHDIV

Ugt71g1   42 ITVFCIKFPGMPFADSYIKSVLASQPQIQLIDLPEVEPPPQELLKSPEFYILTFLESLIP
mmaa10    57 VVMPEV.....SWQLGRSLNCTV..KTYSTSYTLE.....DLDREFMAFAQAQWKAQIQ.
mmaa8     57 VVMPEV.....SWQLGRSLNCTV..KTYSTSYTLE.....DLDREFMTFAHAQWKAQLR.
mmaa7     57 VVMPEV.....SWQLGRSLNCTV..KTYSTSYTLE.....DQDREFMVLADAHWTARLQ.
mmaa9     57 VVMPKV.....SWQLGRPLNCTV..KTYSTSYTLE.....DLDREFKAFAQAQWKAQVQ.
mmaa4a    60 VLTPEV.....KMHVKEENFFTL..TTYAIPWTQD.....EFDRFVLGHTQWFFETEHLL
mmaa4b    60 VLTPEV.....KMHIKEENFFTL..TTYAIPWTQD.....EFDRLVLGHTQWFFETEHLL
mmaa5     60 VLTPEV.....NMHIKEDNFFTL..TTYAVPWTQD.....EFDHFLLGHSQSFFETEHLL
mmaa2     60 VLTPEV.....NMHIKEENFFTL..TTYAIPWTQD.....EFDRLMLGHTQMYFETEHFL
mmaa1     59 VLAPDA.....SLYIREGAFYTL..KTYPVPFQRE.....DVKESFVSLGHNVFENDSFL
mmaa6     58 VVVPEV.....NLLLKESKYYTR..KIYPVPYDQE.....EMKNRYQLFGNNHFAERSFL

Ugt71g1  102 HVKATIKTILSNK.........................VVGLVLDFFCVSMIDVGNEFGI
mmaa10   104 SIFSLVMSSSTGFFDLLFSHCRSLFNDQKLVEYLKESSFDAVFLDPFDVCGLIVAKYFSL
mmaa8    104 SFFSLFLSSSNGFFDLFFSHCRSLFNDQKLVEYLKESSFDAVFLDPFDGCGLIVAKYFSL
mmaa7    104 SAFSLLTSSSNGIFDLFFSNCRSLFKDKKLVEYLKESSFDAVFLDPFDGCGLILAKYFSL
mmaa9    104 SIFSLIMSSYNDIFDLFFSNCRSLFKDKKLVEYLKESSFDAVFLDPFDTCGLIVAKYLSL
mmaa4a   108 KRYSRSMAIMNNMSLVFHRSCVELLHNEALIRHLNATSFDVLLTDPVNLCGAVLAKYLSI
mmaa4b   108 KRYSRSMAIMNNMSLVFHRSCVELLHNEALIRHLNATSFDVLLTDPVNLCGAVLAKYLSI
mmaa5    108 MRFSRSMAIMNNISLIIHRSCVELLHNEALIRHLNATSFDVVLTDPFHLCGAVLAKYLSI
mmaa2    108 KTFSKSMAILKNISLVFHRSCVEILHNETLMRHLNATSFDVVLIDPIYLCGVVLAKYLSI
mmaa1    107 QRVIKTYKKIKKDSAMLLSGCSHLLHNKELMASLAESSFDVMLTDPFLPCGPIVAQYLSL
mmaa6    106 TAPQTEYRNNMVVIGMYFINCQSLLQDVGTLNFLKESKFDALFTDPALPCGVILAEYLGL

Ugt71g1  137 PSYLFLTSNVGFLSLMLSLKNRQIEEVFDDSDRDHQLLNIPGISNQVPSNVLPDACFNKD
mmaa10   164 PSVVFARGIFCHYLEEGAQCPAPLSYVPRELLGFSDAMTFKERVRNHVMHLEEHLFCKYF
mmaa8    164 PSVVFARGIFCHYLEEGAQCPAPLSYVPRILLGFSDAMTFKERVWNHVMHLEEHLFCKYF
mmaa7    164 PSVVFARGTFCHYLEEGAQCPAPLSYIPRGLLGFSDAMTFKERVWNHITHLEEHLFCPYF
mmaa9    164 PSVVFARGVFCHYLEEGAQCPAPLSYVPRILLGFSDAMTFKERVRNHIMHLEERLLCHRF
mmaa4a   168 PTVFFLRNIPCDLDLKGTQCPNPYSYIPKLLTTNSDHMTFLQRVKNMLYPLALSYICDAV
mmaa4b   168 PTVFFLRNIPCDLDFKGTQCPNPYSYIPKLLTTNSDHMTFLQRVKNMLYPLALSYICHAV
mmaa5    168 PTVFFLRNILCDLDFKGTQCPNPYSYIPKFLTTNSDHMTFLQRVKNMLYPLALSYICDTV
mmaa2    168 PAVFFLRYIPCDLDFKGTQCPNPYSYIPKLLTTNSDHMTFLQRVKNILYPLALSYICDAL
mmaa1    167 PTVFFLNALPCSLESEATQCPNPFSYVPRPLSAHSDHMTFLQRVKNMLIAFSQNFLCDVV
mmaa6    166 PSVYLFRGFPCSLEHTFSRSPNPVSYIPRCYTKFSDHMTFPQRVANFLVNLLEPYLFYCL

Ugt71g1  197 GGYIAYYKLAERFRDTKGIIVNTFSDLEQSSIDALYDHDEK.IPPIYAVGPLLDLKGQPNPK
mmaa10   224 FKSALEIASEILQTPVTEYDLYNHTSIWLLRTDFVLDPPKPVMPNMVFIGGINCHQGKPVPK
mmaa8    224 FKNALEIASEILQTPVTAYDLYSHTSIWLLRTDFVLDYPKPVMPNMVFIGGINCHQGKPVPM
mmaa7    224 *KNVLEIASEILQTSVTAYDLYSHTSIWLLRTDFVLDYPKPVMPNMVFIGGINCHQGKPVPM
mmaa9    224 FKSALEIASEILQTPVTVYDLHRQISIWLLRMDFVLDYPKPVMPNMVFIGGINCHQGKPLPM
mmaa4a   228 SAPYASLASELFEREVSVVDLLSHASVWLFRGDFVMDYPRPIMPNMVFIGGINCANRKPLSQ
mmaa4b   228 SAPYASLASELFEREVSVVDLLSHASVWLFRSDFVMDYPRPIMPNMIFIGGINCANRKPLSR
mmaa5    228 SVPYASLASELFQREVSVVDLLSHASVWLFRSDFVMDYPRPIMPNMVFIGGINCANRKPLSQ
mmaa2    228 SAPYASLASELFQREVSVVDLLSHASVWLFRGDFVMDYPRPIMPNMVFIGGINCANRKPLSQ
mmaa1    227 YSPYATLASEFLQREVTVQNLLSSASVWLLRSDFVKDYPRPIMPNMAFIGGINCLHQSPLSQ
mmaa6    226 FSKYDELASAVLKRDVDVITLYQKVSVWLLRYDFVLEYPRPVMPNMVFIGGTNCKKRKDLSE
```
