# Supplementary material for: Adaptive evolution of multiple-variable exons and structural diversity of drug-metabolizing enzymes
Source: BMC Evol Biol. 2007 May 2;7:69. doi: 10.1186/1471-2148-7-69 (PMC1885805; doi:10.1186/1471-2148-7-69)
Supplement: Additional file 20 — Alignment of the protein sequences of UGT71G1 to those of the human (Additional file 17), chimpanzee (Additional file 18), rhesus monkey (Additional file 19), baboon (Additional file 20), dog (Additional file 21), mouse (Additional file 22), rat (Additional file 23), chicken (Additional file 24), and zebrafish clusters A (Additional file 25) and B (Additional file 26) variable UGT1 sequences, as well as the corresponding human (Additional file 27), mouse (Additional file 28), and rat (Additional file 29) UGT2 proteins. Each vertebrate UGT group was aligned by ClustalW and then aligned with UGT71G1 according to the structural alignment shown in the Figure 4A. The ω+ sites predicted to be subject to positive selection with a posterior probability >0.90 by one model and >0.5 by at least one other model are highlighted in red. [file 1471-2148-7-69-S20.pdf]

Additional file 20. Alignment of the baboon (b) UGT1 variable sequences with those of the UGT71G1

```
Ugt71g1    1                     MSMSDINKNSELIFIPAPGIGHLASALEFAKLLTNHDKNLY
ba10       1 MAHTRCTSPVPLCLCLLLTCG...FAEAGKLLVVP.MDGSHWFTMQSVVEKLILRGHEVV
ba8        1 MARTRWTSPVLLCVSLLLTCG...FAEAGKLLVVP.MDGSHWFTMQSVVEKLILRGHEVV
ba7        1 MACAGWTGLLPLSVCLLLTCG...FAEAGKLLVVP.MDGSHWFTMRSVVEKLILRGHEVV
ba9        1 MTRTGWSSPLPLCVCLLLACG...FAEAGKLLVVP.MDGSHWFSMRSVVEKLILRGHEVV
ba5a       1 MATGLQVSLPQLATGLLLLLSVQPWVESGKVLVVP.TDGSHWLSMQDAVRELHARGHQVV
ba5b       1 MATGLQVFLPQLATGLLLLLSVQPWAESGKVLVVP.ADGSHWLSMREAVRELHARGHQAV
ba4        1 MATGLQVSLPQLATGLLLLLSVQPWAESGKVLVVP.TDGSHWLSMREAVRELHARGHQAV
ba2        1 MATGPQVPLLWLATGLLLLLSVQPWAESGKVLVVP.IDGSHWLSMREAVRELHARGHQAV
ba1        1 MAVESQGRHP.LVLGLLLCVLGPVLCHAGKMLLIP.VDGSHWLSMLGAIQQLQQRGHEIV
ba6        1 MACLLRA.FQRISAGVFF.LALWGMVVGDKLLVIP.QDGSHWLSMKDIVEVLSDRGHDIV

Ugt71g1   42 ITVFCIKFPGMPFADSYIKSVLASQPQIQLIDLPEVEPPPQELLKSPEFYILTFLESLIP
ba10      57 VVMPEV.....SWQLGRPLNCTV..KTYSTSYTLE.....DLDREFMTFAQAQWKAQIQ.
ba8       57 VVMPEV.....SWQLGRSLNCTV..KTYSTSYTLE.....DLDREFMTFAHAQWKAQLR.
ba7       57 VVMPEV.....SWQLGRSLNCTV..KTYSTSYTLE.....DQDREFMAFAQAQWKAPLR.
ba9       57 VVIPKV.....SWQLGRSLNCTV..KTYSTSYTLE.....VLDREFKAFAQAQWKAQVQ.
ba5a      60 VLSPEV.....NMHIKEENFFTL..TTYAVPWTQD.....EFDRFLLGHSQSFFETEHLL
ba5b      60 VLTPEV.....NMHIKEENFFTL..TTYAISWTQD.....EYDHLLLGHTQSFFETEHLL
ba4       60 VLTPEV.....NMHIKEENFFTL..TTYAIPWTQD.....EFDRLVLGHTQWFFETEHLL
ba2       60 VLTPEV.....KMHIKEDNFFTL..TTYAIPWTQD.....EFDRLVLGHTQLYFETEHFL
ba1       59 VLAPDA.....SLYIREGAFYTL..KTYPVPFQRE.....DVKESFVSLGHNVFENDSFL
ba6       58 VVVPEV.....NLLLKESKYYTR..KIYPVPYDQE.....EMKNRYQLFGNNHFAERSFL

Ugt71g1  102 HVKATIKTILSNK.........................VVGLVLDFFCVSMIDVGNEFGI
ba10     104 SIFSLFLSSSSGFFDLVFSHCRSLFNDQKLVEYLKESSFDAVFLDPFDICGLIVAKYFSL
ba8      104 SFFSLFLSSSNGFFDLFFSHCRSLFNDQKLVEYLKESSFDAVFLDPFDACGLIVAKYFSL
ba7      104 SAFSLLTSSSNGIFDLFFSNCRSLFKDKKLVEYLKESSFDAVFLDPFDGCGLILAKYLSL
ba9      104 SIFSLLMSSYNDIFDLFFSNCRSLFKDKKLVEYLKESSFDAVFLDPFDTCGLIVAKYLSL
ba5a     108 MRFSRSMAIMNNISLIIHRSCVELLHNEALIRHLNATSFDVVLTDPFHLCGAVLAKYLSI
ba5b     108 MRFSRSMAVMNNMSLIIHRSCVELLHNEALIRHLNATSFDVVLTDPFHLCGAVLAKYLSI
ba4      108 KRYSRSMAIMNNMSLVFHRSCVELLHNEALIRHLNATSFDVVLTDPVNLCGVVLAKYLSI
ba2      108 KTFSKSMAILKNMSLVFHRSCVELLHNETLMRHLNATSFDVVLIDPIYLCGVVLAKYLSI
ba1      107 RRVIKTYKKIKKDSAMLLSGCSHLLHNKELMVSLAESSFDVMLTDPFLPCGPIVAQYLSL
ba6      106 TAPQTEYRNNMVVIGMYFINCQSLLQDVDTLNFLKESKFDALFTDPALPCGVILAEYLGL

Ugt71g1  137 PSYLFLTSNVGFLSLMLSLKNRQIEEVFDDSDRDHQLLNIPGISNQVPSNVLPDACFNKD
ba10     164 PSVVFARGIFCHYLEEGAQCPAPLSYVPRELLGFSDAMTFKERVWNHVIHLEEHLFCKFI
ba8      164 PSVVFARGIACHYLEEGAQCPAPLSYVPRILLGFSDAMTFKERVWNHVMHLEDHLFCKYF
ba7      164 PSVVFARGTFCHYLEEGAQCPAPLSYIPRGLLGFSDAMTFKERVWNHITHLEEHLFCPYF
ba9      164 PSVVFARGIFCHYLEEGAQCPAPLSYVPRILLGFSDAMTFKERVRNHIMHLEERLLCHRF
ba5a     168 PTVFFLRNILCDLDFKGTQCPNPYSYIPKFLTTNSDHMTFLQRVKNMLYPLALSYICDTV
ba5b     168 PAVFFLRNIPCDLDFKGTQCPNPYSYIPKFLTANSDHMTFLQRVKNMLYPVALSYICHAV
ba4      168 PAVFFLRNIPCDLDFKGTQCPNPYSYIPKLLTTNSDHMTFLQRVKNMLYPLALSYICDAV
ba2      168 PAVFFLRNIPCDLDFKGTQCPNPYSYIPKLLTTNSDHMTFLQRVKNMLYPLALSYICDAL
ba1      167 PTVFFLNALPCSLEFEATQCPNPFSYVPRPLSAHSDHMTFLQRVKNMLIAFSQNFLCDVV
ba6      166 PSVYLFRGFPCSLEHTFSRSPNPVSYIPRCYTKFSDHMTFPQRVANFLVNLLEPYLFYCL

Ugt71g1  197 GGYIAYYKLAERFRDTKGIIVNTFSDLEQSSIDALYDHDEK.IPPIYAVGPLLDLKGQPNPK
ba10     224 FKSALEIASEILQTPVTEYDLYSHTSIWLLRTDFVLDPPKPVMPNMVFIGGINCHQGKPLPK
ba8      224 FKNALEIASEILQTPVTEYDLYSHTSIWLLRTDIVLDYPKPVMPNMVFIGGINCHQGKPVPM
ba7      224 LKNVLEIASEILQTSVTAYDLYSHTSIWLLRTDFVLDYPKPVMPNMVFIGGINCHQGKPVPM
ba9      224 FKSALEIASEILQTPVTVYDLHRQISIWLLRTDFVLDYPKPVMPNMVFIGGINCHQGKPLPM
ba5a     228 SVPYASLASELFQREVSVVDLLSHASVWLFRSDFVMDYPRPIMPNMVFIGGINCANRKPLSQ
ba5b     228 SVPYASLASELFQREVSVVDLLSHASVWLFRSDFVMDYPRPIMPNMVFIGGINCANRKPLSQ
ba4      228 SVPYASLASELFQREVSVVDLLSHASVWLFRSDFVMDYPRPIMPNMVFIGGINCANRKPLSR
ba2      228 SAPYASLASELFQREVSVVDLLSHASVWLFRGDFVMDYPRPIMPNMVFIGGINCANRKPLSQ
ba1      227 YSPYATLASEFLQREVTVQNLLSSASVWLLRSDFVKDYPRPIMPNMAFIGGINCLHQSPLSQ
ba6      226 FSKYDELASAVLKRDVDVITLYQKVSVWLLRYDFVLEYPRPVMPNMVFIGGTNCKKRKDLSE
```
