# Supplementary material for: Adaptive evolution of multiple-variable exons and structural diversity of drug-metabolizing enzymes
Source: BMC Evol Biol. 2007 May 2;7:69. doi: 10.1186/1471-2148-7-69 (PMC1885805; doi:10.1186/1471-2148-7-69)
Supplement: Additional file 21 — Alignment of the protein sequences of UGT71G1 to those of the human (Additional file 17), chimpanzee (Additional file 18), rhesus monkey (Additional file 19), baboon (Additional file 20), dog (Additional file 21), mouse (Additional file 22), rat (Additional file 23), chicken (Additional file 24), and zebrafish clusters A (Additional file 25) and B (Additional file 26) variable UGT1 sequences, as well as the corresponding human (Additional file 27), mouse (Additional file 28), and rat (Additional file 29) UGT2 proteins. Each vertebrate UGT group was aligned by ClustalW and then aligned with UGT71G1 according to the structural alignment shown in the Figure 4A. The ω+ sites predicted to be subject to positive selection with a posterior probability >0.90 by one model and >0.5 by at least one other model are highlighted in red. [file 1471-2148-7-69-S21.pdf]

Additional file 21. Alignment of the dog (d) UGT1 variable sequences with those of the UGT71G1

```
Ugt71g1    1                     MSMSDINKNSELIFIPAPGIGHLASALEFAKLLTNHDKNLYI
da11       1 MAATILTGFPLLC..VCLLLTSGFAEAGKLLVVP.MDGSHWFTMRLVVEQLIQRGHELVL
da9        1 MAATILTGFPLLC..VCLLLTSGFAEAGKLLVVP.MDGSHWFTMRLVVEQLIQRGHELVL
da10       1 MAATILTGFPLLC..VCLLLTSGFAEAGKLLVVP.MDGSHWFTMRLVVEQLIQRGHELVL
da8        1 MAATILTGFPLLC..VCLLLTSGFAEAGKLLVVP.MDGSHWFTMRLVVEQLIQRGHELVL
da7        1 MAATILTGFPLLC..VCLLLTSGFAEAGKLLVVP.MDGSHWFTMRLVVEQLIQRGHELVL
da2        1 MAAGFLAPLPVLTGLLLILCDGRWVEGGRVLVVP.MDGSHWLSMKKAVQKLHARGHQMVV
da3        1 MAAGFLAPLPVLTGLLLILCVGRWVEGGKVLVVP.MDGSHWLSMKKAVQKLHARGHQMVV
da4        1 MAAGFLAPLPVLTGLLLILCVGRWVEGGKVLVVP.LDGSHWLSMKKAVQKLHARGHQMVV
da1        1 MAAEAPGPRPLVLGLLLCALSAPVSQGGKLLLIP.VDGSHWLSMLGVVKQLHQRGHEVVV
da6        1 ~~~~~MARLLHLFQKVFFLMLWGEAVGDKLLVVP.QDGSHWLSMENIVELLSEKGHDIVV

Ugt71g1   43 TVFCIKFPGMPFADSYIKSVLASQPQIQLIDLPEVEPPPQELLKSPEFYILTFLESLIPH
da11      58 IIPEV.....SWQLGKSSNFTV..KTYSTTYNLE.....ELNQKFKTFSDSQWNIRQQ.S
da9       58 IIPEV.....SWQLGKSSNFTV..KTYSTAYNLE.....ELNQMFKTFSDSHWNIRQQ.S
da10      58 IIPEV.....SWQLGKSSNFTV..KTYSTTYNLE.....ELNQMFKNFSDSHWNIRQQ.S
da8       58 IIPEV.....SWQLGKSSNFTV..KTYSTTYNLE.....ELNQMFKNFSYSQWKTQQQ.S
da7       58 IIPEV.....SWQLGKSSNFTV..KTYSTTYNLE.....ELNQMFKNFSYSQWKTQQQ.S
da2       60 VSPES.....NMHIKEEDFFTL..TTYATPYTQE.....EFDNFMLGQSYLVFQRMSFLK
da3       60 VSPES.....NMHIKEEDFFTL..TTYATPYTQE.....EFDRILLGQSYLVFERMPFLK
da4       60 VSPES.....NMHIKEEDFFTL..TTYAPPYTRE.....EYDRVLLGQRYQVFERVSFLK
da1       60 IASEA.....SVYIKGAAFYTL..KRYPVPFRRE.....DVEATFTSLGRGVFENVPLLR
da6       55 LVPEV.....NLLLKESKHYTR..QIYSVPFGQE.....ELENRYRSFGKNHFAERWLLN

Ugt71g1  103 VKATIKTILSNK........................VVGLVLDFFCVSMIDVGNEFGIP
da11     105 ILSMVLNSSSDFFEHLFLHCKNLFSDPKLVEYIKESSFDAVFLDPFDVCGLIVAKYFSLP
da9      105 LLTMFFDSSSDIFEHLFLHCKNLFRDTTLIEYIKESSFDAVFLDPFDMCGLIVANYFSLP
da10     105 LLTMFFDSSSDIFEHLFLHCKNLFSDPKLVEYIKESSFDAVFLDPFDVCGLIVAKYFSLP
da8      105 SLFLVLSPLKDSLEHLFLHCKNLFSDPKLVEYIKESSFDAVFLDPFDVCGLIVAKYFSLP
da7      105 SLFLVLSPLKDSLEHLFLHCKNLFSDPKLVEYIKESSFDAVFLDPFDVCGLIVAKYFSLP
da2      108 TFLKTMEGLKTATLIFQRSCESLMHNKNLIRHLNASSFDVLLTDPVYPCGAILARYLSLP
da3      108 TFLKTMEALKAAALIFQRSCESLMHNKNLIRHLNASSFDVLLTDPVYPCGAILARYLSLP
da4      108 RLLKTVERLKTVTSFYQRSCEGLLHNKNLIRHLNASSFDVVLTDPVHPCGAILARYLSLP
da1      108 RVIKTYKKVKEDSALLLSACSHLLHNKELMASLAESSFDAVLTDPFLPCGPIVALYLALP
da6      103 AAQMEYRNSMIVIDMYFTNCQSLLEDSATLSVLRQSKFDALFTDPALPCGVILAEYLGLP

Ugt71g1  138 SYLFLTSNVGFLSLMLSLKNRQIEEVFDDSDRDHQLLNIPGISNQVPSNVLPDACFNKDG
da11     165 SVVFTRGLFCHFLEESTQCPSPPSYVPRIFLGFPDAMSFRERVRNHISHLEEHLFCHYFL
da9      165 SVVFTRGLFCHFLEESTQCPSPPSYVPRIFLGFPDAMSFRERVWNHISHLEEHLFCHYFF
da10     165 SVGFTRRLYCHYLEEATQCPSSPSYVPRPFSILSDVMSFRERVRNHIFHLEEHLFCHYFL
da8      165 SVGFTRRLYCHYLEEATQCPSSPSYVPRPFSILSDVMSFRERVRNHIFHLEEHLFCHYFL
da7      165 SVVFTRGLFCHFFEESTQCPSPLSYVPRPFSMLSDAMSFRERVRNHIFHLEEHLFCHYFL
da2      168 SVFFLRNIPCDLESESTQCPNPSSYIPRLLTRNSDHMTFLQRVKNMLYPLALKYFCHFSF
da3      168 SVFFLRNIPCDLDSEGTQCPNPSSYIPRLLTRNSDHMTFLQRVKNMLYPLALKYICHVSF
da4      168 SVFFLRNIPCDLDSEGTQCPNPSSYIPRLLTRNSDHMTFLQRVKNMLYPLALKYICHFSF
da1      168 AVFFLHALPCSLDFQGTQCPNPPSYVPRALSLNSDHMTFLQRVKNMLIFLSESFLCNVVY
da6      163 SVYLFRGFPCSLEHTISRSPNPVSYIPRCYTQFSDKMTFPQRVGNYLVNYLETYLFYCLY

Ugt71g1  198 GYIAYYKLAERFRDTKGIIVNTFSDLEQSSIDALYDHDEK.IPPIYAVGPLLDLKGQPNPK
da11     225 KTALEVASEILQTAVTPYDLYSHTSIWLLRTDFVFDYPKPVMPNMVFIGGINCQEGKPLPK
da9      225 KTPLEVASEILQTAVTPYDLYSHTSIWLLRADFVFDYPKPVMPNMVFIGGINCQEGKPLPK
da10     225 KTALEVASEILQKAVTPYDLYSHMSIWLLRTDFVFDYPKPVMPNMVFIGGINCQEGKPLPK
da8      225 KTALEVASEILQKAVTPYDLYSHMSIWLLRTDFVFDYPKPVMPNMVFIGGINCQEGKPLPK
da7      225 KTPLEVASEILQTAVTPYDLYSHTSIWLLRADFVLDYPKPVMPNMVFIGGINCQEGKTLPK
da2      228 TPYASLASELLQREVSLEDILSSGSVWLFRGDFVLDYPRPIMPNMFFIGGINCANRKPLSQ
da3      228 TPYASLASELLQREVSLVDILSSGSVWLFRGDFVLDYPRPIMPNMFFIGGINCANRKPLSQ
da4      228 TPYASLASELLQREVSLVDILSSGSVWLFRGDFVLDYPRPIMPNMFFIGGINCANRKPLSQ
da1      228 SPYEPLASEVLQKDVTVQELMGSASIWLLKGDFVKDYSRPIMPSMVFVGGINCASKNPLSK
da6      223 SKYEDLASNILKRDVHLPTLYRNGSIWLLRYDFVFEYPRPVMPNMVFIGGTNCKMKGVLPQ
```
