# Supplementary material for: Adaptive evolution of multiple-variable exons and structural diversity of drug-metabolizing enzymes
Source: BMC Evol Biol. 2007 May 2;7:69. doi: 10.1186/1471-2148-7-69 (PMC1885805; doi:10.1186/1471-2148-7-69)
Supplement: Additional file 22 — Alignment of the protein sequences of UGT71G1 to those of the human (Additional file 17), chimpanzee (Additional file 18), rhesus monkey (Additional file 19), baboon (Additional file 20), dog (Additional file 21), mouse (Additional file 22), rat (Additional file 23), chicken (Additional file 24), and zebrafish clusters A (Additional file 25) and B (Additional file 26) variable UGT1 sequences, as well as the corresponding human (Additional file 27), mouse (Additional file 28), and rat (Additional file 29) UGT2 proteins. Each vertebrate UGT group was aligned by ClustalW and then aligned with UGT71G1 according to the structural alignment shown in the Figure 4A. The ω+ sites predicted to be subject to positive selection with a posterior probability >0.90 by one model and >0.5 by at least one other model are highlighted in red. [file 1471-2148-7-69-S22.pdf]

Additional file 22. Alignment of the mouse (m) UGT1 variable sequences with those of the UGT71G1

```
Ugt71g1    1                    MSMSDINKNSELIFIPAPGIGHLASALEFAKLLTNHDKNL
ma12       1      MAPVAFPTSFFL..CLLLASGLAQAGRLLVVP.MDGSHWFTMQMVVEKLIHRGHEV
ma13       1      MVPAAFPTSLPLCVCLLLASGLVQAGRLLVVP.MDGSHWFDMQMVVEKLIQRGHEV
ma10       1      MAPADFPASLPLCVCLLLASGLAQAGRLLVVP.MDGSHWFTMQTVVEKLLHKGHEV
ma11       1      MAPAVFPASLPLRVCLLLAFGLAQAGRLLVVP.MDGSHWFSMKMIVEKLSHRGHEV
ma7        1    MACLLPAAQTLPAGFLFLVLWASVL.GDKLLVVP.QDGSHWLSMKEIVEHLSERGHDI
ma9        1    MACLLPAAQTLPAGFLFLVLWASVL.GDKLLVVP.QDGSHWLSMKEIVEHLSERGHDI
ma2        1    MDTGLCVPLRGISGLLLLLCALPWAEGAKVLVLP.MEGSQWLSMRDVVRELHARGHQT
ma5        1      MGLRMPLQGLVG.LLLLCALPWTEGEKVLVFP.VGGSHWLSMRDVVRELHAQGHQT
ma1        1 MTVVCWSSRLLLLLPYLLLCVFGPSASHAGRLLVFP.MDGSHWLSMLGVIQQLQQKGHEV

Ugt71g1   41 YITVFCIKFPGMPFADSYIKSVLASQPQIQLIDLPEVEPPPQELLKSPEFYILTFLESLI
ma12      54 VVVIPEV.....SWQLGKSLNCTV..KTYSISHTLE.....DLDREFKYLSYTQWKTP.E
ma13      56 VVVIPEV.....SWRLGKSLNCTV..KTYSVSHTLE.....DLDREFKYFTYTQWKTP.E
ma10      56 VVVVPEV.....SWQLTKPLNFVV..KTYAVSHTQE.....DLNREFKIFIDAQWKSQQE
ma11      56 VVVIPEV.....SWHLGKSQNFTV..KTYSVSYTLE.....DLSHHFNFFAHKQWEN.RE
ma7       57 MVLVPEV.....NLLLGESKYYRR..KIFSVTYSLE.....ELQTRFRTFGNNHFLPGAS
ma9       57 VVLVPEV.....NLLLGESKYYRR..KIFSVPYSLE.....ELQTRFRTFGRNQFVPGAP
ma2       58 VVLASEV.....TVHIKGEDFFTL..KTYAFPYTKE.....EYQQEILSDIEKTFKTQHF
ma5       55 VVLAPEV.....NMRIKEEDFFTF..KVYAVPYTRQ.....EL.EEMMENLKVFFDTGNY
ma1       60 VVIAPEA.....SIHIKEGSFYTL..RKFPVPFQKE.....NVTATLVELGRTAFNQDSF

Ugt71g1  101 PHVKATIKTILSNK.........................VVGLVLDFFCVSMIDVGNEFG
ma12     101 HSIRSFLTGSARGFFELTFSHCRSLFNDKKLVEYLKQRFFDAVFLDPFDVCGLIVAKYFS
ma13     103 QSIRSFMTGSARGFFELMFSHSRGLFNDKKLVEYLKQRSFDAVFLDPFDVCGLIVAKYLS
ma10     104 GGILPLLDSPAKGFFELLFSHCRSLFNDKKLVEYLKQTSFDAVFLDPFDVCGLTVAKYFS
ma11     103 VGMFSLLKHSGKGFFQVLFSHCRSLFNDKKLVEYLKQRSFDAVFLDPFDVCGLTIAKYFS
ma7      105 LMGPLREYRNNMIVVDMFFSNCQSLLKDSATLSFLRENKFDALFTDPAMPCGVILAEYLN
ma9      105 LMGPLREYRNSMLTLEMFFSNCQSLLKDSATLSFLRENKFDALFTDPAMPCGVILAEYLN
ma2      106 VKAFFETTASIRNFFDLYSNSCIALLHNKMLIQQLNSSFFDVILTDPIFPCGAVLAKYLQ
ma5      102 MKKIFKTSEALRNMSTVLLKTCTNILHNESLLHHLNSSSFDVVFTDPVFPCGALLAKYLG
ma1      108 LLRVVKIYMKVKRDSSMLLAGCSHLLHNAEFMASLEESHFDALLTDPFLPCGSIVAQYLT

Ugt71g1  136 IPSYLFLTSNVGFLSLMLSLKNRQIEEVFDDSDRDHQLLNIPGISNQVPSNVLPDACFNK
ma12     161 LPSVIFARGVFCDYLEEGAQCPSLPSYVPRLFSKYTDTMTFKERVWNHLIYIEEHAFCSY
ma13     163 LPSVIFARLSFCYYLEEGAQCPSLLSYVPRLFSKYTDTMTFKERVWNHYMYIEDYVFCPY
ma10     164 LPSVVFSRGIFCHYLEDAAQCPSPPSYIPRMLLKFTDTMTFKERTRNLLAYMGERAFCHK
ma11     163 LPSVVFSGGIFCHYLDEGAQCPSPPSYVPRILSKFADTMTFKERVWNYFSYMKERAFCPY
ma7      165 LPSVYLFRGFPCSLEHMLGQSPSPVSYVPRFYTKFSDHMTFPQRLANFIVNILENYLYYC
ma9      165 LPSVYLFRGFPCSLEHMLGQSPSPVSYVPRFYTKFSDHMTFPQRLANFIVNILENYLYYC
ma2      166 IPAVFILRSLSCGIEYEATQCPNPSSYIPNLLTRLSDHMDFLQRVQNMLYYLVLKYICRL
ma5      162 IPAVFFLRYIPCGIEYEATQCPSPSSYIPNLFTRLSDHMDFLQRVQNMLYHLVLKYICHL
ma1      168 VPTVYFLNKLPCSLDSEATQCPVPLSYVPKSLSFNSDRMNFLQRVKNVLLAVSENFMCRV

Ugt71g1  196 DGGYIAYYKLAERFRDTKGIIVNTFSDLEQSSIDALYDHDEK.IPPIYAVGPLLDLKGQPNPK
ma12     221 FLRTAVEVASEILQTPVTMTDLFSPVSIWLLRTDFVLEFPRPVMPNMVFIGGINCLQKKSLSK
ma13     223 FFKTAVEIASEVLQTPVTMTDLFSPVSIWLLRTDFVLEFPRPVMPNMVFVGGMNCLQGKPLSK
ma10     224 FFKSAADIASEVLQTPVTMTDLFSPVSIWLLRTDFVLEFPRPVMPNVIYIGGINCHQGKPLSK
ma11     223 FFKTAIEIASEVLQTPVTMTDLFSSVSIWMFRTDFVLEFPRPVMPNMVYIGGINCHPGKPLSK
ma7      225 LYSKYEIIASDLLKRDVSLPSLHQN.SLWLLRYDFVFEYPRPVMPNMIFLGGINCKKKGKLTQ
ma9      225 LYSKYEIIVTDLLKRDVSLPSLHQN.SLWLLRYDFVFEYPRPIMPNMIFIGGINCKKKGKLTQ
ma2      226 SITPYESLASELLQREVSLVEVLSHASVWLFRGDFVLDYPRPIMPNMVFIGGINCVTKKPLSQ
ma5      222 LITPYESLASELFQREVSSVELFSYASVWLFRGDFVLDYPRPIMPNMVFIGGINCVTKKPLSQ
ma1      228 VYSPYGSLATEILQKEVTVQDLLSPASIWLMRSDFVKDYPRPIMPNMVFIGGINCLQKKPLSQ
```
