# Supplementary material for: Adaptive evolution of multiple-variable exons and structural diversity of drug-metabolizing enzymes
Source: BMC Evol Biol. 2007 May 2;7:69. doi: 10.1186/1471-2148-7-69 (PMC1885805; doi:10.1186/1471-2148-7-69)
Supplement: Additional file 23 — Alignment of the protein sequences of UGT71G1 to those of the human (Additional file 17), chimpanzee (Additional file 18), rhesus monkey (Additional file 19), baboon (Additional file 20), dog (Additional file 21), mouse (Additional file 22), rat (Additional file 23), chicken (Additional file 24), and zebrafish clusters A (Additional file 25) and B (Additional file 26) variable UGT1 sequences, as well as the corresponding human (Additional file 27), mouse (Additional file 28), and rat (Additional file 29) UGT2 proteins. Each vertebrate UGT group was aligned by ClustalW and then aligned with UGT71G1 according to the structural alignment shown in the Figure 4A. The ω+ sites predicted to be subject to positive selection with a posterior probability >0.90 by one model and >0.5 by at least one other model are highlighted in red. [file 1471-2148-7-69-S23.pdf]

Additional file 23. Alignment of the rat (r) UGT1 variable sequences with those of the UGT71G1

```
Ugt71g1    1                     MSMSDINKNSELIFIPAPGIGHLASALEFAKLLTNHDKNL
ra8        1 ~~~~MAPADIPASLPLGLCLLLASGFGHAGKLLVVP.MDGSHWFTMQMVVEKLLPKGHEV
ra9        1 ~~~~MAPSGCPPSLPLCVCLFLASGFAQAGRLLVVP.MDGSHWFTMQMIVEKLSHRGHEV
ra11       1 ~~~~MAPSGCPSSLPLCVCLFLASGFAQAGRLLVVP.MDGSHWFTRQMTVEKLLQRGHEV
ra1        1 MSVVCRSSCSLLLLPCLLLCVLGPSASHAGKLLVIP.IDGSHWLSMLGVIQQLQQKGHEV
ra2        1 ~~MDTGLCAPLRGLSGLLLLLCALPWAEGGKVLVFP.MEGSHWLSMRDVVRELHARGHQA
ra6        1 ~~~~MGLHVTLQGLAGLLLLLYALPWAEGGKVLVFP.MEGSHWLSMRDVVRELHARGHQA
ra4        1 ~~~~MGIQGFLQKLSGLLLLLCALPWAEGGKVLVFP.MEGSHWLSMRDVVRELHARGHQA
ra7        1 ~~~~MACLLPAARLPAGFLFLVLWGSVLGDKLLVVP.QDGSHWLSMKEIVEHLSERGHDI

Ugt71g1   41 YITVFCIKFPGMPFADSYIKSVLASQPQIQLIDLPEVEPPPQELLKSPEFYILTFLESLI
ra8       56 VVVVPEV.....SWQLGKPLNFTV..KTYSVSHTQE.....DLNREFKFFIDSQWKTQQE
ra9       56 VVVIPEV.....SWHMGKSLNFTV..KTYSVSYTLE.....DLNYHFKFFAHNQWKT.QE
ra11      56 GVVIPEE.....SWQLGKLVNCTV..KTYSIPYTVE.....DMDREFKHFSYTQWKTP.E
ra1       60 VVIAPEA.....SIHIKEGSFYTM..RKYPVPFQNE.....NVTAAFVELGRSVFDQDPF
ra2       58 VVLAPEV.....TVHMKGEDFFTL..QTYAFPYTKE.....EYQREILGNAKKGFEPQHF
ra6       56 VVLAPEV.....TVHIKEEDFFTL..QTYPVPYTRQ.....GFRQQMMRNIKVVFETGNY
ra4       56 VVLAPEV.....TVHIKEEDFFTL..QTYPVPYTKE.....EYRHHLLGHLQNFFETEFS
ra7       56 VVLVPEV.....NLLLGESKYYRR..KSFPVPYNLE.....ELRTRYRSFGNNHFAASSP

Ugt71g1  101 PHVKATIKTILSNK........................VVGLVLDFFCVSMIDVGNEFG
ra8      104 GGVLPLLTSPAQGFFELLFSHCRSLFKDKKLVEYLKQSSFDAVFLDPFDVCGLTVAKYFS
ra9      103 VGMFSLLKHSGKGFFELLFSHCRSLFKDKKLVEYLKQSSFDAVFLDPFDVCGLILAKYFS
ra11     103 QSMYSLITGSVKDFLEITFSHCRSLFKDKKLVEYLKQSSFDAVFLDPFHVCGLTVAKYLS
ra1      108 LLRVVKTYNKVKRDSSMLLSGCSHLLHNAEFMASLEQSHFDALLTDPFLPCGSIVAQYLS
ra2      106 VKTFFETMASIKKFFDLYANSCAALLHNKTLIQQLNSSSFDVVLTDPVFPCGALLAKYLQ
ra6      104 VKTFLETSEILKNISTVLLRSCMNLLHNGSLLQHLNSSSFDMVLTDPVIPCGAVLAKYLG
ra4      104 LKLVLQRMAAVNNVSTFYVRSCRGLLHNTALIQSLNSSSFDVVLTDPFFPCGAVLAMYLR
ra7      104 LMAPLREYRNNMIVIDMCFFSCQSLLKDSATLSFLRENQFDALFTDPAMPCGVILAEYLK

Ugt71g1  136 IPSYLFLTSNVGFLSLMLSLKNRQIEEVFDDSDRDHQLLNIPGISNQVPSNVLPDACFNK
ra8      164 LPSVVFSRGIFCHYLEEGSQCPSPPSYVPRGILKLTDTMTFKERVWNLLSYMGEHAFCPS
ra9      163 LPSVVFSGGIFCHYLDEGAQCPSPPSYVPRILSKFTDTMTFKERVWNHLSYMKERAFCPY
ra11     163 LPSIIFAKDIFCDNLKEGAQCPSLPSYVPRLFSKSADTMSFKERLWNHLIYFEERAFCSY
ra1      168 LPAVYFLNALPCSLDLEATQCPAPLSYVPKSLSSNTDRMNFLQRVKNMIIALTENFLCRV
ra2      166 IPAVFFLRSVPCGIDYEATQCPKPSSYIPNLLTMLSDHMTFLQRVKNMLYPLTLKYICHL
ra6      164 IPTVFFLRYIPCGIDSEATQCPKPSSYIPNLLTMLSDHMTFLQRVKNMLYPLALKYICHF
ra4      164 VPAVFFLQSMLCELEFEATNSPNPSSYIPRLLTLNSDHMSFLDRVKNMLYPVPWMYLCHV
ra7      164 LPSVYLFRGFPCSLEHMLGQSPSPVSYVPRFYTKFSDHMTFPQRLANFIANILENYLYHC

Ugt71g1  196 DGGYIAYYKLAERFRDTKGIIVNTFSDLEQSSIDALYDHDEK.IPPIYAVGPLLDLKGQPNPK
ra8      224 FFKTATDIASEVLQTPVTMTDLFSPVSVWLLRTDFTLELPRPVMPNVIHIGGINCHQRKPLSK
ra9      223 FFKTAVEIASEVLQTPVTMRDLFSPVSIWMFRTDFVLEFPRPVMPNMVYIGGINCHQGKPLSK
ra11     223 YLKTAIEIASEVLQTPVTLTDLYSPVSIWLLRTDFVFNFPRPVMPNMVFVGGINCFQRKPLSK
ra1      228 VYSPYGSLATEILQKEVTVKDLLSPASIWLMRNDFVKDYPRPIMPNMVFIGGINCLQKKALSQ
ra2      226 SITPYESLASELLQREMSLVEVLSHASVWLFRGDFVFDYPRPIMPNMVFIGGINCVIKKPLSQ
ra6      224 SFTRYESLASELLQREVSLVEVLSHASVWLFRGDFVFDYPRPVMPNMVFIGGINCVIKKPLSQ
ra4      224 NYGSLARLASDLLQREVSVVEILRHASVWLLRKDFVFYYPRPFMPNMVFIGGINCANRKPLSQ
ra7      224 LYSKYEILASDLLKRDVSLPALHQN.SLWLLRYDFVFEYPRPVMPNMIFIGGTNCKKKGNLSQ
```
