# Supplementary material for: Adaptive evolution of multiple-variable exons and structural diversity of drug-metabolizing enzymes
Source: BMC Evol Biol. 2007 May 2;7:69. doi: 10.1186/1471-2148-7-69 (PMC1885805; doi:10.1186/1471-2148-7-69)
Supplement: Additional file 24 — Alignment of the protein sequences of UGT71G1 to those of the human (Additional file 17), chimpanzee (Additional file 18), rhesus monkey (Additional file 19), baboon (Additional file 20), dog (Additional file 21), mouse (Additional file 22), rat (Additional file 23), chicken (Additional file 24), and zebrafish clusters A (Additional file 25) and B (Additional file 26) variable UGT1 sequences, as well as the corresponding human (Additional file 27), mouse (Additional file 28), and rat (Additional file 29) UGT2 proteins. Each vertebrate UGT group was aligned by ClustalW and then aligned with UGT71G1 according to the structural alignment shown in the Figure 4A. The ω+ sites predicted to be subject to positive selection with a posterior probability >0.90 by one model and >0.5 by at least one other model are highlighted in red. [file 1471-2148-7-69-S24.pdf]

Additional file 24. Alignment of the chicken (*Gallus gallus* [gg]) UGT1 variable sequences with those of the UGT71G1

```
Ugt71g1    1                    MSMSDINKNSELIFIPAPGIGHLASALEFAKLLTNHDKNLY
gga10      1 MTLRL.CYPLAAWIFILLL...PGLSEGGKLLVVP.MIGSHWLSMKEVVQKLAEKGHEVV
gga14      1 MTLRL.CCPLAAWIFILLV...PGLSEGGKLLVVP.MIGSHWLSMKEVVQKLAEKGHEVV
gga11      1 MTLRL.CYPLAAWIFILLL...PGLSEGGKLLVVP.MVGSHWLSMKEVVQKLAERGHEVV
gga13      1 MTLRL.CYPLAAWIFILLV...PGLSEGGKLLVVP.VVGSHWLSMKEVVQKLAERGHEVV
gga12      1 MTLQL.CYPLAAWIFILLV...PGLSEGGKLLVVP.MVGSHWLSMKEVVQKLAERGHEVV
gga5       1 MALVLPSHPQVS.VSLLLLLSVLGLAAGGKLLVVP.IDGSHWLSMREVLDELRQKGHEIV
gga6       1 MALVLPSHPQVL.VSLLLLLSVLGLAAGGKLLVVP.VDGSHWLSMREVLDGLRQKGHEIV
gga1       1 MALVLPSHPQVS.VSLLLLLSVLSLAAGGKLLVVS.VDGSPWFSVLEMLEVLKQKGHEIV
gga7       1 MALVLPSHPQVS.VSLLLLLSVLSLAAGGKLLVVP.VGGSHWLSMQEVLDGLRQKGHEIV
gga3       1 MALVLPSHPQVS.VSLLLLLSVLSLAAGGKLLVVS.VDGSPWFSVLEMLEVLKQKGHEIV
gga4       1 ~MASSHPGFHFLASWAVILLLLRIFSEGGKLLVIP.IDGSHWLSMRLVVEQLRHRGHEIV
gga8       1 ~~MDSFSPCNMLMAVLLPFLCYLNTAAAGKLLVIP.MDGSHWLSMKEVLAELSSRGHEIV
gga9       1 .MALLFLCFYPRTGIFFLLLSSLIFAECGKILVIP.QDGSHWLSMQPVVGKLQQNGHDVV

Ugt71g1   42 ITVFCIKFPGMPFADSYIKSVLASQPQIQLIDLPEVEPPPQELLKSPEFYILTFLESLIP
gga10     56 VLKPEV.....SWNTGDKHNHAYTVKTYPVSTKLE.....ELDNAFKVYVATHLKGLPFP
gga14     56 VLKPEV.....SWNTGDKHNHAYTVKTYPVSTKLE.....DLDNAFKVYLATHLKSMPFP
gga11     56 VLKPEV.....SWQMGETQKHAYTVKTYPVSTKLE.....ELDNAFKAYVATHLKSLPFP
gga13     56 VLMPEV.....SWQMGETQKHAYTVKTYPVSTKLE.....ELDNAFKVYLATHLKSMPFP
gga12     56 VLKPEV.....SWQMGETQKHAYTVKTYPVSTKLE.....ELDNAFKLFLDPFLKDLPFP
gga5      59 VVAPEV.....SLYIKPSENFIM..KMYPVPFTQE.....EVDGMFRRTGQDAFMEGSFL
gga6      59 VVAPEV.....NVHIKPTQNFVM..KMYPVPFTQE.....EMDSSFREFSQDVFAEGSFL
gga1      59 VVAPEA.....NLNVKPSESFIL..KTYPASFTQE.....EMDDNFQAFLKDAFEEGSFL
gga7      59 IVSPEF.....NLFVKPSNNFTM..KLYQVPVTEE.....EINGDFQAFLKEAFEEGSFV
gga3      59 VVAPEA.....SLNVKPSESFIL..KTYPASSKQK.....GMAGNLQLFLQEALEEGSFL
gga4      59 VVAPEI.....NLRIGASMHYSM..KTYSVSYTRE.....FVEAEFKKLGYKSFTPQTFL
gga8      58 VVAPDS.....SILIDSSGNYTM..KTYPVPYKQE.....EMEELLHTIGTTSFSEFPLL
gga9      59 VVVPSS.....SLFMKSKEHQNYTVKVYRIPYEEE.....HLHLVLKSFVNDHFTEQSLL

Ugt71g1  102 HVKATIKTILSNK.........................VVGLVLDFFCVSMIDVGNEFGI
gga10    106 LYALALYNISTYLFESFYAQCKELFSSTETLQYLNQSSFDAVLTDPLFMCGATVANYLSL
gga14    106 KNVIALHNIFTYLFESFYAQCKELFSSTETLQYLNQSGFDAVLTDPFFMCGATVANYLSL
gga11    106 LNVLALYNISSNAFSTFFLQCKELFSSTETLQYLNQSGFDAVLTDPIFMCGATLANYFSL
gga13    106 LNVLALYNVSVGVFHTFFLQCKELFSSTETLQYLNQSGFDAILTDPVLMCGATVANYLSL
gga12    106 ENIIAPYKNGIHLFKTFYLECRELFSSTETLHYLNQSSFDAILTDPFFMCGATVANYLSL
gga5     107 ERFIKAYQGVKNSSAMFLSTCEQLLYNKELIRYLQENKFDAVFTDPILPCGQILAEHLSV
gga6     107 EKLATIRQRLQRMSAIFLSTCEHLLYNKELIRYLQEYEFDALFTDPFFPCGQILAEHLSI
gga1     107 EQFHRLQEKVKRLFDVGFISCAWLLKNKELIRYLEESNFDALFTDPVFPCGAILAEHLSI
gga7     107 ERFLRVRERVKKLYELGFIACEQLLKNKELIRYLEESNFDALFTDPVLPCGAIVAEHLSI
gga3     107 GRFFRVRENMKNLSASAVIDCENLLYNKELIKYLEESKFDALLTDPILSCGAILAEYLSI
gga4     107 EKF....SKITNITTMFFDSCKRLLSNKELMKYLEESMFDGVLMDPFFPCGQIVAEHLSI
gga8     106 TRLLDTWEHFRKTSAMFQAACSSLLYKEELMKFMEESKFDAVFTDPLTPCGQIVALHLSI
gga9     109 NIIITMYQNIIEISRFFFTTCESLLHNKEMMQYLRKSKFDVVFTDPILMCGPILADYLSV

Ugt71g1  137 PSYLFLTSNVGFLSLMLSLKNRQIEEVFDDSDRDHQLLNIPGISNQVPSNVLPDACFNKD
gga10    166 PFVFFMRGLPCNLHFEAAQCPSPLSYVPRTFSFSSDHMTFFQRVENTLISFLELFYCYGY
gga14    166 PFVFFMRGLPCNLHFEAAQCPSPLSYVPRTFSFNSDHMTFFQRVENTLISFLELFYCNAI
gga11    166 PFVFFMRGLPCNLHFEAAQCPSPLSYVPRTFSFSSDHMTFFQRVENTLISFLELFYCNGY
gga13    166 PFVIFMRGFPCNLHYEATQCPSPLSYVPRLFSFNSDHMTFFQRVENALISFLELFYCDDS
gga12    166 PFVFFMRGFPCNLHYEAAQCPSPLSYIPRATSLNSDHMTFFQRVLNTLISLLEQFHCNAI
gga5     167 PSVFYLQQIPCGLEFEATQCPNPPSYIPRIFTDNTDHMNFLQRVENVIFSISNFFLCDVV
gga6     167 PSVFLLQQIPCGLDIDATQCPNPPSYVPRIFSGNSDHMNFLQRVKNIIFDIPHSILCHLL
gga1     167 PSVYFMRGMPCGLDFEATQCPNPPSYIPRAFTDHTDHMNFLQRVKNVIFDTSNLFLCDFI
gga7     167 PSVYLLRGIPCGLHLEATQCPSPPSYIPRLFTDHTDHMNFLQRVKNVIFDTSNLFLCDFL
gga3     167 PSVYFMRLIPCGFDSEASQCPSPPSYIPRAFSDLTDHMNFLQRVKNVIFDTSNLFLCDFL
gga4     163 PSVYLVRGLPCSLDFHATLCPNPPSYIPRFFTHYTDRMGFFQRLGNLLVSLSSTLTCSFL
gga8     166 PTIFFLRGVPCSIDIYAAQSPSPPSYVPRLFSYSTDHMTFPQRVKNLLISISEYFTCSTI
gga9     169 PSVYFLRGFPCGMDSKATQCPSPPSYVPRLFLNNSDSMTFAQRMKNMLVSILELVYCQPL

Ugt71g1  197 GGYIAYYKLAERFRDTKGIIVNTFSDLEQSSIDALYDHDEK.IPPIYAVGPLLDLKGQPNPK
gga10    226 YQAAIQLSSEVLQRDVTPLDLLNSASISIMRFDFVFEYPRPVMPNMVFVGGMNCAEKKPLPK
gga14    226 YQDAIKFSSEVLQRDVSLIDLLNSASISIMRFDFVFEYPRPVMPNMVFVGGMNCAEKKPLPK
gga11    226 YRAAIQLSSEVLQRDVSLIDLLNSAAVSIMRFDFVFEYPRPVMPNMVFVGGINCAQKKPLPK
gga13    226 YRAAIQLSSEVLQRDVSLIDLLNSASIWLMRYDFVFEYPRPVMPNMVFIGGINCAQKKPLSK
gga12    226 YQDAIKFSSEVLQRDVSLLDLLNSASIWLLRYDFVFEYPRPVMPNMVFIGGMNCAQKKPLSK
gga5     227 FQPYAKLASEFLQHDVTVPGLLSKASIWLIKLDFVLHYPRPLMPNMIMVSGVNCAHKK.LTQ
gga6     227 FQPYTKLASEFLQRDVTVLDLLRKASIWLVRFDFVFHYPKPLMPNMIIIGGITCTHKK.LSQ
gga1     227 FKPYEKLASEFLQRDVTVIDLLRKASVWLLRYDFVLDYPRPLMPNIIVVGGINCAHKQ.LPQ
gga7     227 FKPYEKLASEFLQRDVTVLDLLRKASIWLLRYDFVLDYPGPLMPNIIVVGGVNCAHKQ.LPQ
gga3     227 FKPYDKLASEFLQRDVTLLDVFHKASIWLLRYDFVLDYPRPLMPNMIVVGGVNCAHKQ.LPQ
gga4     223 YSPYDHLIKEFLQQEATLLELLSHASIWLMKYDFVFEYPRPVMPNMVLIGGITCTQEKQISK
gga8     226 FSPFERLASDFLQQPMTLTQLLSHGSIWLKRTDFAFDYPMPVMPNMVFIGGINCGRKKLLAQ
gga9     229 FTQFEELAYEILQKKVTATDLLSHGSVWLMRYDFVFEFPRPTMPNMVFIGGINCAQKNKLSQ
```
