# Supplementary material for: Adaptive evolution of multiple-variable exons and structural diversity of drug-metabolizing enzymes
Source: BMC Evol Biol. 2007 May 2;7:69. doi: 10.1186/1471-2148-7-69 (PMC1885805; doi:10.1186/1471-2148-7-69)
Supplement: Additional file 25 — Alignment of the protein sequences of UGT71G1 to those of the human (Additional file 17), chimpanzee (Additional file 18), rhesus monkey (Additional file 19), baboon (Additional file 20), dog (Additional file 21), mouse (Additional file 22), rat (Additional file 23), chicken (Additional file 24), and zebrafish clusters A (Additional file 25) and B (Additional file 26) variable UGT1 sequences, as well as the corresponding human (Additional file 27), mouse (Additional file 28), and rat (Additional file 29) UGT2 proteins. Each vertebrate UGT group was aligned by ClustalW and then aligned with UGT71G1 according to the structural alignment shown in the Figure 4A. The ω+ sites predicted to be subject to positive selection with a posterior probability >0.90 by one model and >0.5 by at least one other model are highlighted in red. [file 1471-2148-7-69-S25.pdf]

Additional file 25. Alignment of the zebrafish UGT1 cluster a (z1a) variable sequences with those of the UGT71G1

```
Ugt71g1    1                   MSMSDINKNSELIFIPAPGIGHLASALEFAKLLTNHDKNLYIT
z1a2       1 ~~~~~~~~MACLWLLGSLLLLGSAEAGKLLVIP.TDGSHWLGLKPIVEELGRRGNQVVVV
z1a4       1 ~~~~~~~~MACLWLLVGLFLLGSAEAGKLLVIP.SDGSHWLGMKPVVEELGRRGNQVVVV
z1a5       1 ~~~~~~~~~MASVLLFCLFCLASAKAGKILVVP.SDGSHWMGIKPLVEELGRRGNQVVVV
z1a6       1 ~~~~~~~~MAAFVLLLYLFCLATAEAGNILVVP.SDGSHWTGMKPLVEELGRRGNQVVVV
z1a7       1 ~MNDRIVRMAASALVLCLFCLVSAEAGNLLVIP.ALGSHWTGMRPLVEELGRRGNRVVVV
z1a1       1 ~MRTLPVPAQGLLALLCLFSFESVQAGKVLVLP.VDGSHWLSMKILVEELSNRGHEMVVL
z1a3       1 MKRTLFVPALGLFAFLCLFSSESVQAGKVLVMP.VDGSHWLSMKILVEEMSSRGHEMVVL

Ugt71g1   44 VFCIKFPGMPFADSYIKSVLASQPQIQLIDLPEVEPPPQELLKSPEFYILTFLE..SLIP
z1a2      52 IPEA.....SLSMGPSEKTTT..LTYPVNYTKV.....ELEAVLASELNALLSIDVSTDL
z1a4      52 IPEA.....SLSMGPSEKTTT..LTYPVNYTKA.....ELHMVLEGNLTEILSTDFSTEV
z1a5      51 IPEA.....SLSMGPSQHTTT..LTYPVPYTKA.....QIQDHISASVTNLISTHVSTDL
z1a6      52 IPEA.....SLSMGPSQHTTT..LSYPVSYTKA.....QIQEIVKAGVTTLISTYVSTDL
z1a7      59 FPEE.....NVNMVPAKHTTT..LTYPVPYTKA.....QIQKGTDAAISKLFSADVSSDV
z1a1      59 VPET.....SILIKKSGKYST..KTYPVSFTHD.....DLAENL.KEIQNSALEKAPK.L
z1a3      60 VPET.....SILIGKSGNFTT..KSFRVPYSFD.....ELNAHV.DHIRKTAIEKAPR.F

Ugt71g1  102 HVKATIKTILSN.........................KVVGLVLDFFCVSMIDVGNEFGI
z1a2     100 AKFQSFFFTLNVLQTFILRNAEGLLFNKDLMKKLQDYNFDAILTDPFEPVGAIAGEYLSI
z1a4     100 SKFFVFFLQLKVLQNFIVRNAEGLLFNEDLMKKLQDCRFDAILTDPFEPVGVIASEYLSI
z1a5      99 ARFQSFINTMDLLSNLITRNVEGLLLNKDLMKKLQDYNFDVILTDPFETVGVIAAEYLSI
z1a6     100 PRFQSFINRMNSLSNIIIRSAEGFLSNKDLIKKLQDYNFDVILTDPFELVGVIAAEYLSV
z1a7     107 GRFQNFFTTMDMLKVIISRNAEGLLLNKDLLKKLKDYNFDAILTDPFETVGVIASEYLSI
z1a1     105 TDIVVNFRNLLQFLTMQSKTCEGLLYNEPLMKSLREMGFDAMLTDPFLPCGTIIADSFSI
z1a3     106 IDIVGALGNLIQFTNMQVKACEGLLYDEPLMKSLRDMKFDALLTDPFLPCGSVIADYFSI

Ugt71g1  137 PSYLFLTSNVGFLSLMLSLKNRQIEEVFDDSDRDHQLLNIPGISNQVPSNVLPDACFNKD
z1a2     160 PAIYMQINHPCGVDTLASQCPAPPSYVPQTFTHFTDRMNLWQRSINFVRALLQPKACKLI
z1a4     160 PAIYMQVNLPCGVDSLASQCPSPPSYVPQRNTHFTDQMNIWQRCINLVRTLLQHMACRYM
z1a5     159 PTIYIQTSHPCGADTLASQCPSPPSYVPQRLTHFSDRMNLWQRSVNFVRTLIQPMACRRM
z1a6     160 PAIYIQTSHPCNVDTLASQCPSPPSYVPHILTHFSDRMNLWQRSVNFIRTLIQPMACRRM
z1a7     167 PAIYMQTSHPCGADVLASQCPAPPSYVPKGLTHFTDRMNLWQRSVNFVRTLVQPVACSRM
z1a1     165 PAVYFLRLIPCRLDEAAAQCPSPPSFMPRYSSGFTDKMTFPQRLVNTLLTVVEGFLCRSM
z1a3     166 PAVYFLRGIPCRLDEAAAQCPSPPSFIPRFFTGYTDKMTFPQRMINTFMTVFEKYLCHQL

Ugt71g1  197 GGYIAYYKLAERFRDTKGIIVNTFSDLEQSSIDALYDHDEK.IPPIYAVGPLLDLKGQPNPK
z1a2     220 FARADEIASHLLQRKTSMVEIFSRAALWFLRFNFALEFPRPVMPNMVMIGATVNQKPKPLTH
z1a4     220 YAEADEIASRFLQRRASIVEIMNRATLWLMRFDFAFEFPRPLMPNMVMIGGMATKKPKPLSK
z1a5     219 FTRADEIASRVLQRKTSIMDIMGRAALWFVHSDFALEFPHPLMPNMIIVGGMDNRKAEPLSQ
z1a6     220 FTRADEIASRVLQRKTSIMDIMSHAALWFVHSDFALEFPHPLMPNMIIVGGMDNTKAEPLSQ
z1a7     227 FAHADEIASQVLQKKTSVMEIMSRAALWFMHFDFAFEFPRPVMPNMVVIGGVDTKKPEPLSQ
z1a1     225 YESADELASKYLQKETTYAELLGHGAVWLLRYDFAFEFPRPQMPNMVQIGGINCVKRAPLTK
z1a3     226 FASFDELATRYLKKDTSYAELLGHGAVWLLRYDFSFEYPKPQMPNMVQIGGINCAKRAPLTK
```
