# Supplementary material for: Adaptive evolution of multiple-variable exons and structural diversity of drug-metabolizing enzymes
Source: BMC Evol Biol. 2007 May 2;7:69. doi: 10.1186/1471-2148-7-69 (PMC1885805; doi:10.1186/1471-2148-7-69)
Supplement: Additional file 26 — Alignment of the protein sequences of UGT71G1 to those of the human (Additional file 17), chimpanzee (Additional file 18), rhesus monkey (Additional file 19), baboon (Additional file 20), dog (Additional file 21), mouse (Additional file 22), rat (Additional file 23), chicken (Additional file 24), and zebrafish clusters A (Additional file 25) and B (Additional file 26) variable UGT1 sequences, as well as the corresponding human (Additional file 27), mouse (Additional file 28), and rat (Additional file 29) UGT2 proteins. Each vertebrate UGT group was aligned by ClustalW and then aligned with UGT71G1 according to the structural alignment shown in the Figure 4A. The ω+ sites predicted to be subject to positive selection with a posterior probability >0.90 by one model and >0.5 by at least one other model are highlighted in red. [file 1471-2148-7-69-S26.pdf]

Additional file 26. Alignment of the zebrafish UGT1 cluster b (z1b) variable sequences with those of the UGT71G1

```
Ugt71g1    1                             MSMSDINKNSELIFIPAPGIGHLASALEFAKLLTNHDK
z1b4       1 MHLGVI.ICLVVGVVCSAVVDQKLSRNSSWTGKLLVVP.MDGSHWTGVKAVAEEMGRRGH
z1b5       1 MH.GVI.FNLVVGVVCSAAMNQKPSRNSSWTGKLLVVP.MDGSHWTGVKAVAEEMGRRGH
z1b2       1 MRLGVI.FILVVGVVCSAAVDQKPSRKSSWTGKLLVVP.MDGSHWTGVKAVAEEMGRRGH
z1b3       1 MHLGVI.FCLVVGVVCSAAVDQKLSRKSSWTGKLLVVP.MDGSHWTGVKAVAEEMGRRGH
z1b7       1 ~~MGLVAFIWCIVLTFASAGEAVKSKEASWTGSLLVVP.MDGSHWTGVKALAEEMGRRGH
z1b1       1 ~MLKKGWLWGLGSGLLLLWLSAGLRPV..QGGRVLVMP.VEGSHWLSMKVLATELARRGH

Ugt71g1   39 NLYITVFCIKFPGMPFADSYIKSVLASQPQIQLIDLPEVEPPPQELLKSPEFYILTFLES
z1b4      59 TVIVVIPEI.....SMRLGPGKHYIT..KKFPVQYKQD.....LINQIMVEHVNEVAASE
z1b5      58 TVIVVIPEI.....SMRLGPGKHYIT..KKFPVKYKPD.....VLNQMIAKLVNTLSAPE
z1b2      59 TVIVVIPEI.....SVLLGPGQHYIT..KMFSVKYDQK.....SLNKVLNERVLEVTNPG
z1b3      59 TVIVVIPEI.....SMRLGPGKHYIT..KKFPVKYDQK.....LFNEVLTEHVHEVTNPG
z1b7      58 KVTVVIPEV.....SVLLGPGKHYVT..RTFPVLYGKQ.....QLDELQARNAQVMESKQ
z1b1      57 DVLVLVPEK.....NILIQSSELFRT..ETFPVKISKE.....QLSKSLKGFQQGVFTRS

Ugt71g1   99 .LIPHVKATIKTILSN.........................KVVGLVLDFFCVSMIDVGN
z1b4     107 QSLLKTVTSIMYRFGKIIDHFISTSESLFQEQELIEFLRDQNFDAVLTNPAMPMGAILAY
z1b5     106 QSLLKRVTSNIYNFGKVVDYFISTTESLFQEQELIEFLRDQNFDAVLTDPAMPMGAILAY
z1b2     107 NSFLSTVIRIVSNLRRMFNTMAATSESLFQDKELIEFLRNENFDAVLTDPVLPMGAILAY
z1b3     107 HSRLKTVTSTMANLLKMFNMMASTSESLFQDKELIKFLRDENFDAILTDPALPMGAVLAY
z1b7     106 LPLMEKISTRFSNMRKFVNLQRATAESLLLNQELVDFLRKQNFDAVLTSPAVPTGAILAY
z1b1     105 .PALMDVFVQLERLLNFTGSQVEGCESLLYNEPLMRKLKEQNFELMLTDPFLPCGPIIAT

Ugt71g1  133 EFGIPSYLFLTSNVGFLSLMLSLKNRQIEEVFDDSDRDHQLLNIPGISNQVPSNVLPDAC
z1b4     167 NLSVPAVYMLRGIMC..ESKATACPNPPSYIPRFFTQISDRMTFSERVINTLVSLLEPLM
z1b5     166 NLSVPAVYMLRGLMC..ESKATACPDPPSYIPRLFTQNSDHMNFGERLMNTLASLMEPLM
z1b2     167 NLSVPAVYLLRGMACGLDATATTCPNPSSYIPRFHTRNTDRMSFGERVMNMLMSILEQTV
z1b3     167 NFSVPAVYMLRGMPCALDATATACPNTPSYIPRFHTGNTDRMSFGERVMNVLMSILEQVA
z1b7     166 NLSLPAVYMLRGLPCGLDSVATACPNPPSYIPRFFTHSSDRMSFGQRVLNVLVSMLEPLL
z1b1     164 ALGVPAVYFLRGMPCGIDLLASQCPSPPSYVPRFHSGTSDKMNFVERIRNFFMSGFELVL

Ugt71g1  193 FNKDGGYIAYYKLAERFRDTKGIIVNTFSDLEQSSIDALYDHDEK.IPPIYAVGPLLDLKGQPNPK
z1b4     225 CRLMLGSFEEMTSKFLHRDVSITEILSTAAVWLMRYDFTLEFPKPLMPNMITIGGINCEVKNPLTK
z1b5     224 CKLKFSAFEEVTSKFLHRDVSMTEILSTGAVWLMRYDFTLEFPKPLMPNIINIGGINCEVNNPLTK
z1b2     227 CKFMYRSFEEMIFNFLQRDVSMTEILRTGAVWLMRYDFTLEFPKPLMPNMQFIGGINCGVRNPLTK
z1b3     227 CKVMYWSFEEVTSNFLQRDVSLTEILSTGAVWLMRYDFTLEFPKPLMPNMQFIGGINCGVKNPLMK
z1b7     226 CRLIYWTTEDVASRFMQRDVSVTEVLSSGALWLLRYDFTLEFPKPLMPNMVLIGGINCAIRHPLTK
z1b1     224 CKVMYASFDELAARYLKKDVTYKEIIGRGALWLHRYDFTFEYPRPIMPNMVFIGGINCQKSAEISA
```
