# Supplementary material for: Adaptive evolution of multiple-variable exons and structural diversity of drug-metabolizing enzymes
Source: BMC Evol Biol. 2007 May 2;7:69. doi: 10.1186/1471-2148-7-69 (PMC1885805; doi:10.1186/1471-2148-7-69)
Supplement: Additional file 27 — Alignment of the protein sequences of UGT71G1 to those of the human (Additional file 17), chimpanzee (Additional file 18), rhesus monkey (Additional file 19), baboon (Additional file 20), dog (Additional file 21), mouse (Additional file 22), rat (Additional file 23), chicken (Additional file 24), and zebrafish clusters A (Additional file 25) and B (Additional file 26) variable UGT1 sequences, as well as the corresponding human (Additional file 27), mouse (Additional file 28), and rat (Additional file 29) UGT2 proteins. Each vertebrate UGT group was aligned by ClustalW and then aligned with UGT71G1 according to the structural alignment shown in the Figure 4A. The ω+ sites predicted to be subject to positive selection with a posterior probability >0.90 by one model and >0.5 by at least one other model are highlighted in red. [file 1471-2148-7-69-S27.pdf]

Additional file 27. Alignment of the human (h) UGT2 sequences with those of the UGT71G1

```
Ugt71g1    1               MSMSDINKNSELIFIPAPGIGHLASALEFAKLLTNHDKNLYITVFC
h2a1       1 ~~~MLNNLLLFSLQISLIGTTLGGNVLIWP.MEGSHWLNVKIIIDELIKKEHNVTVLVAS
h2b15      1 MSLKWTSVFLLIQLSCYFSSGSCGKVLVWP.TEYSHWINMKTILEELVQRGHEVTVLTSS
h2b17      1 MSLKWMSVFLLMQLSCYFSSGSCGKVLVWP.TEYSHWINMKTILEELVQRGHEVIVLTSS
h2b11      1 MTLKWTSVLLLIHLSCYFSSGSCGKVLVWA.AEYSHWMNMKTILKELVQRGHEVTVLASS
h2b28      1 MALKWTSVLLLIHLGCYFSSGSCGKVLVWT.GEYSHWMNMKTILKELVQRGHEVTVLASS
h2b10      1 MALKWTTV.LLIQLSFYFSSGSCGKVLVWA.AEYSLWMNMKTILKELVQRGHEVTVLASS
h2b7       1 MSVKWTSVILLIQLSFCFSSGNCGKVLVWA.AEYSHWMNIKTILDELIQRGHEVTVLASS
h2b4       1 MSMKWTSALLLIQLSCYFSSGSCGKVLVWP.TEFSHWMNIKTILDELVQRGHEVTVLASS
h2a3       1 MRSDKSALVFLLLQLFCVGCGFCGKVLVWP.CDMSHWLNVKVILEELIVRGHEVTVLTHS

Ugt71g1   47 IKFPGMPFADSYIKSVLASQPQIQLIDLPEVEPPPQELLKSPEFYILTFLESLIPHVKAT
h2a1      57 G.....ALFITPTSNPSLTFEIYRVPFGKE.....RIEGVIKDFVLTWLENRPSPSTIWR
h2b15     60 A.....STLVNASKSSAIKLEVYPTSLTKN.....DLEDSLLKILDRWIYGVSKN.TFWS
h2b17     60 A.....SILVNASKSSAIKLEVYPTSLTKN.....DLEDFFMKMFDRWTYSISKN.TFWS
h2b11     60 A.....SILFDPNDASTLKFEVYPTSLTKT.....EFENIIMQQVKRWS.DIRKD.SFWL
h2b28     60 A.....SILFDPNDAFTLKLEVYPTSLTKT.....EFENIIMQQVKRWS.DIQKD.SFWL
h2b10     59 A.....SILFDPNDSSTLKLEVYPTSLTKT.....EFENIIMQLVKRLS.EIQKD.TFWL
h2b7      60 A.....SILFDPNNSSALKIEIYPTSLTKT.....ELENFIMQQIKRWS.DLPKD.TFWL
h2b4      60 A.....SISFDPNSPSTLKFEVYPVSLTKT.....EFEDIIKQLVKRWA.ELPKD.TFWS
h2a3      60 K.....PSLIDYRKPSALKFEVVHMPQDRT.....EENEIFVDLALNVLPGL....STWQ

Ugt71g1  107 IKTILSN.............................KVVGLVLDFFCVSMIDVGNEFGIP
h2a1     107 FYQEMAKVIKDFHMVSQEICDGVLKNQQLMAKLKKSKFEVLVSDPVFPCGDIVALKLGIP
h2b15    109 YFSQLQELCWEYYDYSNKLCKDAVLNKKLMMKLQESKFDVILADALNPCGELLAELFNIP
h2b17    109 YFSQLQELCWEYSDYNIKLCEDAVLNKKLMRKLQESKFDVLLADAVNPCGELLAELLNIP
h2b11    108 YFSQEQEILWELYDIFRNFCKDVVSNKKVMKKLQESRFDIVFADAVFPCGELLAALLNIR
h2b28    108 YFSQEQEILWEFHDIFRNFCKDVVSNKKVMKKLQESRFDIIFADAFFPCGELLAALLNIP
h2b10    107 PFSQEQEILWAINDIIRNFCKDVVSNKKLMKKLQESRFDIVFADAYLPCGELLAELFNIP
h2b7     108 YFSQVQEIMSIFGDITRKFCKDVVSNKKFMKKVQESRFDVIFADAIFPCSELLAELFNIP
h2b4     108 YFSQVQEIMWTFNDILRKFCKDIVSNKKLMKKLQESRFDVVLADAVFPFGELLAELLKIP
h2a3     106 SVIKLNDFFVEIRGTLKMMCESFIYNQTLMKKLQETNYDVMLIDPVIPCGDLMAELLAVP

Ugt71g1  138 SYLFLTSNVGFLSLMLSLK.NRQIEEVFDDSDRDHQLLNIPGISNQVPSNVLPDACFNKD
h2a1     167 FMYSLRFSPASTVEKHCGKVPYPPSYVPAVLSELTDQMSFTDRIRNFISYHLQDYMFETL
h2b15    169 FLYSLRFSVGYTFEKNGGGFLFPPSYVPVVMSELSDQMIFMERIKNMIHMLYFDFWFQIY
h2b17    169 FLYSLRFSVGYTVEKNGGGFLFPPSYVPVVMSELSDQMIFMERIKNMIYMLYFDFWFQAY
h2b11    168 FVYSLRFTPGYTIERHSGGLIFPPSYIPIVMSKLSDQMTFMERVKNMIYVLYFDFWFQMS
h2b28    168 FVYSLCFTPGYTIERHSGGLIFPPSYIPVVMSKLSDQMTFMERVKNMIYVLYFDFWFQMC
h2b10    167 FVYSHSFSPGYSFERHSGGFIFPPSYVPVVMSKLSDQMTFMERVKNMLYVLYFDFWFQIF
h2b7     168 FVYSLSFSPGYTFEKHSGGFIFPPSYVPVVMSELTDQMTFMERVKNMIYVLYFDFWFEIF
h2b4     168 FVYSLRFSPGYAIEKHSGGLLFPPSYVPVVMSELSDQMTFIERVKNMIYVLYFEFWFQIF
h2a3     166 FVLTLRISVGGNMERSCGKLPAPLSYVPVPMTGLTDRMTFLERVKNSMLSVLFHFWIQDY

Ugt71g1  197 .GGYIAYYKLAERFRDTKGIIVNTFSDLEQSSIDALYDHDEK.IPPIYAVGPLLDLKGQPNPK
h2a1     227 .WKSWDSYYSKALGRPTTLCETMGKAEIWLIRTYWDFEFPRPYLPNFEFVGGLHCKPAKPLPK
h2b15    229 DLKKWDQFYSEVLGRPTTLFETMGKAEMWLIRTYWDFEFPRPFLPNVDFVGGLHCKPAKPLPK
h2b17    229 DLKKWDQFYSEVLGRPTTLFETMGKAEMWLIRTYWDFEFPRPFLPNVDFVGGLHCKPAKPLPK
h2b11    228 DMKKWDQFYSEVLGRPTTLFETMGKADIWLMRNSWSFQFPHPFLPNVDFVGGFHCKPAKPLPK
h2b28    228 DMKKWDQFYSEVLGRPTTLFETMGKADIWLMRNSWSFQFPHPFLPNIDFVGGLHCKPAKPLPK
h2b10    227 NMKKWDQFYSEVLGRPTTLSETMRKADIWLMRNSWNFKFPHPFLPNVDFVGGLHCKPAKPLPK
h2b7     228 DMKKWDQFYSEVLGRPTTLSETMGKADVWLIRNSWNFQFPYPLLPNVDFVGGLHCKPAKPLPK
h2b4     228 DMKKWDQFYSEVLGRPTTLSETMAKADIWLIRNYWDFQFPHPLLPNVEFVGGLHCKPAKPLPK
h2a3     226 DYHFWEEFYSKALGRPTTLCETVGKAEIWLIRTYWDFEFPQPYQPNFEFVGGLHCKPAKALPK
```
