# Supplementary material for: Adaptive evolution of multiple-variable exons and structural diversity of drug-metabolizing enzymes
Source: BMC Evol Biol. 2007 May 2;7:69. doi: 10.1186/1471-2148-7-69 (PMC1885805; doi:10.1186/1471-2148-7-69)
Supplement: Additional file 28 — Alignment of the protein sequences of UGT71G1 to those of the human (Additional file 17), chimpanzee (Additional file 18), rhesus monkey (Additional file 19), baboon (Additional file 20), dog (Additional file 21), mouse (Additional file 22), rat (Additional file 23), chicken (Additional file 24), and zebrafish clusters A (Additional file 25) and B (Additional file 26) variable UGT1 sequences, as well as the corresponding human (Additional file 27), mouse (Additional file 28), and rat (Additional file 29) UGT2 proteins. Each vertebrate UGT group was aligned by ClustalW and then aligned with UGT71G1 according to the structural alignment shown in the Figure 4A. The ω+ sites predicted to be subject to positive selection with a posterior probability >0.90 by one model and >0.5 by at least one other model are highlighted in red. [file 1471-2148-7-69-S28.pdf]

Additional file 28. Alignment of the mouse (m) UGT2 sequences with those of the UGT71G1

```
Ugt71g1    1                 MSMSDINKNSELIFIPAPGIGHLASALEFAKLLTNHDKNLYITV
m2a1       1 ~~~MLKNIL.LCSLQIS.LLGMSLGGNVLIWP.MEGSHWLNVKIIIDELLRKEHNVTVLV
m2b38      1 ~~MPGKWISALLLLQISCCFRSVKCGKVLVWP.MEFSHWMNIKIILDELVQRGHEVTVLR
m2b5       1 ~~MPGKWISALLLLQISCCFRSVKCGKVLVWP.MEFSHWMNIKIILDELVQRGHEVTVLR
m2b37      1 ~~MPGKWISALLLLQISCCFRSVKCGKVLVWP.MEFSHWMNIKIILDELVQRGHEVTVLR
m2b35      1 ~~MPVKWISALLLLQMSCCFRSTSCGKVLVWP.LEFSHWMNLKIILDELVQRGHEVTVLR
m2b36      1 ~~MLWKWISALLLLQISCCFRSAKCGKVLVWP.VDYSHWMNIKIILDELKQKGHEVTVLR
m2b1       1 ~~MSMKQASVFLLIQFICYIRPGACGKVLVWP.TEYSHWINMKIILDELVQRGHDVTVLI
m2b34      1 MPVKMTAALLLLLLQLSGFFGSGTGGKVLVWP.MEFSHWLNLKTILDELLKKGHEVMVLR
m2a3       1 ~MVSEKCVAAFFLLQL.CWAGCGFCSKVLVWP.CDMSHWLNLKTILEELGARGHEVTVLK

Ugt71g1   45 FCIKFPGMPFADSYIK.SVLASQPQIQLIDLPEVEPPPQELLKSPEFYILTFLESLIPHV
m2a1      55 ASG.....ALFITPSSISPSLTFEIYPVPFGKE.....KIESVIKDFVLTWLENRPSPST
m2b38     58 PSA.....YYVLDPKK.SPGLKFETFPTSVSKD.....NLENFFIKFVDVWTYEMPR.DT
m2b5      58 PSA.....YYVLDPKK.SPGLKFETFPTSVSKD.....NLENFFIKFVDVWTYEMPR.DT
m2b37     58 PSA.....YYVLDPKK.SPGLKFETFPTSVTKD.....DLENFFIQLLNVWTYELSR.DT
m2b35     58 PSA.....SIFVDPKY.SPGLKFETFPTAFSKD.....YLETFLTKLVDEWTFEVPR.DT
m2b36     58 PST.....SIFLDPKK.SPGLKFETFPTSFSND.....VMEIIFAKAVERWTYEVPR.DT
m2b1      58 SSA.....SILIGPSN.ESSINFEIYSAPLSKD.....DLEYAFEKWVGNWTYELKK.LP
m2b34     60 PSA.....SLSYEVDN.TSAIEFETYPTSYSLS.....ELEEIFWESLKKYIYELPK.QS
m2a3      58 .YP.....SIIIDQSK.RIPLHFENIPLLYEIE.....TAENRLNEIANLAVNVIPN.LS

Ugt71g1  104 KATIKTILSN.............................KVVGLVLDFFCVSMIDVGNEF
m2a1     105 IWTFYKEMAKVIEEFHLVSRGICDGVLKNEKLMSKLQKEKFEVLLSDPVFPCGDIVALKL
m2b38    106 CLSYSPLLQNMIDEFSDYFLSLCKDVVSNKELMTKLQESKFDVLLSDPVASCGELIAELL
m2b5     106 CLSYSPLLQNMIDEFSDYFLSLCKDVVSNKELMTKLQESKFDVLLSDPVASCGELIAELL
m2b37    106 CLSYSPLMQNMFDELSGYYLSLCKDVVSNRQLMTKLQESKFDVLLSDPVAFGGELIAELL
m2b35    106 CLSYS.LLQTLFGKLSDYSLSLCKEAVSNKQLMTKFQESKFDVLILDAMASCGELIAQLL
m2b36    106 CLSYSPLLQNIFDEYSDYCLTLCKDTVSNKQLMAKLQESKFDVILSDAIGPCGELIAELL
m2b1     106 FWTSYSKLQKISSEYSDMIESFCKAVVWNKSLMKKLQGSKFDVVLADALVPCGELLSELL
m2b34    108 FWGYFLMLQEMVWVDSKYFESLCKDVVFNKELMTKLQKSRFDVILADPFIPCGDLLAEVL
m2a3     105 LWEAAKTLQDFFLQVTGDFESICRSVLYNQKFMDKLRDAQYDVVVIDPVVPCGELVAEVL

Ugt71g1  135 GIPSYLFLTSNVGFLSLMLSLK.NRQIEEVFDDSDRDHQLLNIPGISNQVPSNVLPDACF
m2a1     165 GIPFIYSLRFSPASTVEKHCGKVPFPPSYVPAILSELTDQMSFTDRVRNFISYRMQDYMF
m2b38    166 QIPFLYSIRFSPGYQIEKSSGRFLLPPSYVPVILSGLGGQMTFIERVKNMICRLYFDFWF
m2b5     166 QIPFLYSIRFSPGYQIEKSSGRFLLPPSYVPVILSGLGGQMTFIERIKNMICMLYFDFWF
m2b37    166 HIPFLYSLRFTAGYRIEKSSGRFLLPPSYVPVILSGLGGQMTFIERVKNMICMLYFDFWF
m2b35    165 QIPFLYSLRFSPGYQVEKNSGGFVLPPSYVPVILSGLGGQMTFTERVKNMICMLYFDFWF
m2b36    166 QIPFLYSLRFSPGYYLEKYSGGLPLPPSYVPVILSGLSGQMTFKERVKNMICMLYFDFWF
m2b1     166 KTPLVYSLRFCPGYKCEKYSGGLPLPPSYVPVVLSELSDHMTFAERVKNMLQVLLFDFWF
m2b34    168 KIPLVYSLRFFPGSTYEKYSGGLPLPPSYVPVVMSELSDRMTFMERVRNVIYMLCFDFWF
m2a3     165 QIPFVYTLRFSMGYYMEKHCGQLPIPLSYVPVVMSELTDNMTFTERVKNMMFSLLFEYWL

Ugt71g1  194 NKD.GGYIAYYKLAERFRDTKGIIVNTFSDLEQSSIDALYDHDEK.IPPIYAVGPLLDLKGQPNPK
m2a1     225 ETL.WKQWDSYYTKALGRPTTLCETMGKAEIWLMRTYWDFEFPRPYLPNFEFVGGLHCKPAKPLPK
m2b38    226 QMFNDKKWDSFYSEYLGRPTTLAETMGKAEMWLIRSNWDLEFPHPTLPNVDYVGGLHCKPAKPLPK
m2b5     226 QMFNDKKWDSFYSEYLGRPTTLVETMGQAEMWLIRSNWDLEFPHPTLPNVDYVGGLHCKPAKPLPK
m2b37    226 QMPNDKKWDSFYTEYLGRPTTLAETMGQAEMWLIRSNWDLEFPHPTLPNVDYVGGLHCKPAKPLPK
m2b35    225 QTFTEKEWDQFYSETLGRPTTLIETMGKAEMWFIRSYWDLEFPHPTLPNVEYVGGLHCKPAKPLPK
m2b36    226 QTFREKKWDQFYSETLGRPTTLIETMGKAEMWLIRSYWDLEFPHPTLPNVYYVGGVHCKPAKPLPK
m2b1     226 QTFNEKSWNQFYSDVLGRPTTLTEMMGKADIWLVRTFWDLKFPHPFLPNFDFVGGLHCKPAKPLPK
m2b34    228 QTFNEKNWNQLYTEVLGRPTTLSETMAKADIWLIRTYWDLEFPHPVLPNFDFIGGLHCRPAKPLPK
m2a3     225 QQYDFAFWDQFYSETLGRPTTFCKTVGKADIWLIRTYWDVEFPRPYLPNFEFVGGLHCKPAKPLPK
```
