# Supplementary material for: Adaptive evolution of multiple-variable exons and structural diversity of drug-metabolizing enzymes
Source: BMC Evol Biol. 2007 May 2;7:69. doi: 10.1186/1471-2148-7-69 (PMC1885805; doi:10.1186/1471-2148-7-69)
Supplement: Additional file 29 — Alignment of the protein sequences of UGT71G1 to those of the human (Additional file 17), chimpanzee (Additional file 18), rhesus monkey (Additional file 19), baboon (Additional file 20), dog (Additional file 21), mouse (Additional file 22), rat (Additional file 23), chicken (Additional file 24), and zebrafish clusters A (Additional file 25) and B (Additional file 26) variable UGT1 sequences, as well as the corresponding human (Additional file 27), mouse (Additional file 28), and rat (Additional file 29) UGT2 proteins. Each vertebrate UGT group was aligned by ClustalW and then aligned with UGT71G1 according to the structural alignment shown in the Figure 4A. The ω+ sites predicted to be subject to positive selection with a posterior probability >0.90 by one model and >0.5 by at least one other model are highlighted in red. [file 1471-2148-7-69-S29.pdf]

Additional file 29. Alignment of the rat (r) UGT2 sequences with those of the UGT71G1

```
Ugt71g1    1                  MSMSDINKNSELIFIPAPGIGHLASALEFAKLLTNHDKNLYITV
r2a1       1 ~~~MLKNIL.LWSLQLSL.LGMSLGGNVLIWP.MEGSHWLNVKIIIDELLRKEHNVTVLV
r2b3       1 ~~MPGKWISALLLLQISCCFQSGNCGKVLVWP.MEFSHWMNIKTILDELVQRGHEVTVLK
r2b6       1 ~~MPGKWIFALLLLQISFCLRSAKCGKVLVWP.MEFSHWMNIKTILDELVQRGHEVTVLK
r2b2       1 ~~MPRKWISALFLLQISYCFKSGHCGKVLVWP.MDFSHWMNIKIILDELVQRGHEVTVLK
r2b12      1 ~~MPGKWISALLLLQISFCFKSGNCGKVLVWP.MEYSHWMNIKIILEELVQKGHEVTVLR
r2b8       1 ~~MPQKWISALLLLQISFCFRSGNCGKVLVWP.LEYSHWMNLKIILDELVQRGHEVTVLR
r2b34      1 MSVKRTASLLLLLLQLSDFFGSGTGGKVLVWP.MEFSHWLNLRVILDELLKKGHEVTVLR
r2b39      1 MSVKMTAA..LLLLHLTIFFGSGSGGKVLVWP.VEFSHWLNLKIILSELVKKGHEVVVLK
r2b1       1 MSMKQTS..VFLLIQLICYFRPGACGKVLVWP.TEYSHWINIKIILNELAQRGHEVTVLV
r2a3       1 ~MVSVKWAVAIFLLQL.CWSGCGICSKVLVWP.CDMSHWLNLKTILEELAARGHEVTVLK

Ugt71g1   45 FCIKFPGMPFADSYIKSVLASQPQIQLIDLPEVEPPPQELLKSPEFYILTFLESLIPHVK
r2a1      55 ASG.....ALFITPSVSPSLTFEIYPVPFGKE.....KIESVIKDFVLTWLENRPSPSTI
r2b3      58 PSA.....YYVLDPKKSPDLKFETFPTSVSKD.....ELENYFIKLVDVWTYELQR.DTC
r2b6      58 PSA.....YYVLDPKKSPDLKFETFPTSVSKD.....ELEKYFIKLVDAWTYELQR.DTC
r2b2      58 PSA.....YFFLDPKKSSDLKFEIFSTSISKD.....ELQNHFIKLLDVWTYELPR.DTC
r2b12     58 PSA.....SVFLDPKETSDLKFVTFPTSFSSH.....DLENFFTRFVNVWTYELPR.DTC
r2b8      58 PSS.....SVFLDPKKASGLVYETFPTTSNND.....EVEKFFTQWVNTWTYDVPK.YTC
r2b34     60 PSA.....SLSYEVDNTSAIEFETYPTSYSLT.....EVDEFFWESLRKYIYELPK.QSF
r2b39     58 PSV.....SFSYEVDKTSAIEFESFPSSYSIA.....DVEVIFMDCVNESIYELPK.QSF
r2b1      58 SSA.....SILIEPTKESSINFEIYSVPLSKS.....DLEYSFAKWIDEWTRDFET.LSI
r2a3      58 .YP.....SIIIDQSKLTPLQFENIPVLYETE.....VAENHLNEIVNLAVNVIPN.LSL

Ugt71g1  105 ATIKTILSNK.............................VVGLVLDFFCVSMIDVGNEFG
r2a1     105 WTFYKEMAKVIEEFHLVSRGICDGVLKNEKLMTKLQRGKFEVLLSDPVFPCGDIVALKLG
r2b3     107 LSYSPLLQNMIDGFSDYYLSLCKDTVSNKQLMAKLQESKFDVLLSDPVAACGELIAEVLH
r2b6     107 LSFSPLLQNMMDEFSDYYLSVCKDAVSNKQLMAKLQESKFDVLLSDPVAACGELIAEVLH
r2b2     107 LSYSPILQNLVYEFSYFYLSICKDAVSNKQLMTKLQESKFDVLFADPVASCGDLIAELLH
r2b12    107 LSYFLYLQDTIDEYSDYCLTVCKEAVSNKQFMTKLQESKFDVVFSDAIGPCGELIAELLQ
r2b8     107 LRYYPSLNKMFGQFSDLWLQLCREVVSNKHLMAKLKESQFDVVLSDAVGPCGELIAEILQ
r2b34    109 WGYFLMFQELVWVDSDYFESLCKDVVFNKELMTKLQNSGFDVILADPFIPCGDLLAEILK
r2b39    107 WGYFLMLQKLFERTSDYSERLCRDAAFNKELMTKLQNSGFDVILADPFTPCGDLLAEILK
r2b1     107 WTYYSKMQKVFNEYSDVVENLCKALIWNKSLMKKLQGSQFDVILADAVGPCGELLAELLK
r2a3     106 WEAARTLQDFFLQLTGNFEDLCRSTLYNQTLMNKLRDAKYDVMVLDPVIPCGELVAEVLQ

Ugt71g1  136 IPSYLFLTSNVGFLSLMLSLK.NRQIEEVFDDSDRDHQLLNIPGISNQVPSNVLPDACFN
r2a1     165 IPFIYSLRFSPASTVEKHCGKVPFPPSYVPAILSELTDQMSFADRVRNFISYRMQDYMFE
r2b3     167 IPFLYSLRFSPGYKIEKSSGRFILPPSYVPVILSGMGGPMTFIDRVKNMICTLYFDFWFH
r2b6     167 IPFLYSLRASPGHKIEKSSGRFILPPSYVPVILSGLGGQMTFIDRVKNMICMLYFDFWFH
r2b2     167 IPFLYSLSFSPGHKLEKSIGKFILPPSYVPVILSGLAGKMTFIDRVKNMICMLYFDFWFE
r2b12    167 IPFLYSLRFSPGYTIEKYIGGVLFPPSYVPMIFSGLAGQMTFIERVHNMICMLYFDFWFQ
r2b8     167 LPFVYSLRFGIGYGIEKYSAGQPFPPSYVPIILSGLSGQMTFMERVENMLCLLYFDFWFE
r2b34    169 IPLVYSLRFFPGSTYEKYSGGLPMPPSYVPIAMSELSDRMTFVERMKHMIYVLCFDFWFQ
r2b39    167 IPLVYSLRFFPGSTYEKYSGGLPMPPSYVPIAMSELSDRMTFVERVKHMIYVLCFDFWFQ
r2b1     167 TPLVYSLRFCPGYRCEKFSGGLPLPPSYVPVVLSELSDRMTFVERVKNMLQMLYFDFWFQ
r2a3     166 IPFVNTLRFSMGYSMEKYCGQLPVPLSYVPVVMGELTDHMTFTERVKNMMLSLFFEFWLQ

Ugt71g1  195 KD.GGYIAYYKLAERFRDTKGIIVNTFSDLEQSSIDALYDHDEK.IPPIYAVGPLLDLKGQPNPK
r2a1     225 TL.WKQWDSYYSKALGRPTTLCETMGKAEIWLMRTYWDFEFPRPYLPNFEFVGGLHCKPAKPLPK
r2b3     227 MFNAKKWDPFYSEILGRPTTLAETMGKAEMWLIRSYWDLEFPHPTLPNVDYIGGLQCRPPKPLPK
r2b6     227 MFNAKNWDPFYTEILGRPTTLAETMGKAEMWLIRSYWDLEFPHPTLPNVDYIGGLQCKPAKPLPK
r2b2     227 ILRHKEWDTFYSEILGRPTTVDETMSKVEIWLIRSYWDLKFPHPTLPNVDYIGGLHCKPAKPLPK
r2b12    227 TFREKKWDPFYSKTLGRPTTLAEIMGKAEMWLIRSYWDLEFPHPISPNVDYIGGLHCKPAKPLPK
r2b8     227 SFPAKDWDPFFSEILGRPTTMVDTMKKAEIWLIRSYWDLEFPRPSLPNIEFVGGLHCQPAKPLPK
r2b34    229 AFNEKKWNELYTEVLGRPTTLSETMAKADIWLIRTYWDLEFPHPVLPNFDFVGGLHCRPAKPLPK
r2b39    227 VFDEKKWNELYTEVLGRPTTLSETMAKADIWLIRTYWDLEFPHPVLPNFDFVGGLHCRPAKPLPK
r2b1     227 PFKEKSWSQFYSDVLGRPTTLTEMMGKADIWLIRTFWDLEFPHPFLPNFDFVGGLHCKPAKPLPR
r2a3     226 QYDFAFWDQFYSKTLGRPTTFCKTVGKAEIWLIRTYWDIEFPRPYLPNFEFVGGLHCKPAKPLPK
```
